# Global, regional, and national burden and trends of depressive disorders among women of childbearing age from 1990 to 2021: insights from GBD 2021

**DOI:** 10.3389/fpsyg.2025.1594430

**Published:** 2025-08-13

**Authors:** Mingjie Tang, Yinghong Li, Jun Shao, Shiwei Li, Jiqin Tang, Zhaoming Chen

**Affiliations:** ^1^Nanjing Hospital of Chinese Medicine Affiliated to Nanjing University of Chinese Medicine, Nanjing, China; ^2^Graduate School of Nanjing University of Chinese Medicine, Nanjing, China; ^3^Xiangya School of Medicine, Central South University, Changsha, China; ^4^College of Rehabilitation Medicine, Shandong University of Traditional Chinese Medicine, Jinan, China

**Keywords:** depressive disorders, global burden of disease, GBD 2021, DALYs, women of childbearing age

## Abstract

**Background:**

Depressive disorders are highly prevalent and disabling mental illnesses among women of childbearing age (WCBA), especially with the surge in prevalence following the COVID-19 pandemic, but evidence on their burden remains insufficient. This study provides a thorough analysis of the burden of depressive disorders among WCBA at global, regional and national levels.

**Methods:**

This study utilized data from Global burden of disease (GBD) 2021 to evaluate the trends in incidence, prevalence, Disability-Adjusted Life Years (DALYs) of depressive disorders among WCBA from 1990 to 2021. Data was stratified by time, age, region, and socio-demographic index (SDI). Moreover, this study utilized a range of analytical methods, including estimated annual percentage change (EAPC), Joinpoint regression, decomposition analysis and predictive modeling (Nordpred method).

**Results:**

Depressive disorders in WCBA constitute a growing global health crisis, marked by a notable rise in absolute cases, accompanied by a slight downward age-standardized incidence rate (ASIR), age-standardized prevalence rate (ASPR), and age-standardized DALYs rate (ASDALYR). In 2021, there were 133.25 million incident cases, 121.24 million prevalent cases, and 21.04 million DALYs. In 2021, the global ASIR, ASPR, and ASDALYR were 6,808.01 (95% uncertainty interval [UI]: 5,049.99 to 9,106.66) per 100,000, 6173.45 (95% UI: 4,883.27 to 7,781.73) per 100,000, and 1,073.50 (95% UI: 686.73 to 1,562.48) per 100,000. High SDI region had the highest ASIR, ASPR, and ASDALYR. Furthermore, High-income North America exhibited the highest ASIR, ASPR and ASDALYR. Joinpoint regression analysis yielded a pronounced increase in ASIR subsequent to 2019. Nordpred predictive model demonstrated an upward trend in the ASIR, ASPR and ASDALYR of depressive disorders among WCBA by 2046.

**Conclusion:**

Depressive disorders among WCBA show a globally rising burden, particularly in high SDI region, with a notable post-2019 increase. This underscores an urgent need for tailored public health action alleviate the growing mental health burden, particularly among WCBA.

## 1 Introduction

Depressive disorders, defined by enduring low mood, diminished interest, and pervasive hopelessness, have emerged as a significant global public health issue (Barnett, 2019; WHO, 2023). The incidence of depressive disorders has increased in recent decades, impacting approximately 332 million individuals worldwide (IHME, 2024). The Global Burden of Disease (GBD) 2021 study estimates over 300 million cases of depressive disorders worldwide, with a prevalence rate of approximately 4,006.82 per 100,000 individuals, thereby placing a substantial segment of the global population at immediate risk of developing these disorders (Rong et al., 2025). This imposes significant pressure on healthcare systems and society at large. From 2010 to 2021, the age-standardized disability-adjusted life years (DALY) for depressive disorders rose by 16.4% (95% uncertainty interval [UI]: 11.9–21.3), marking the second most significant increase among the 25 primary tertiary causes (GBD 2021 Diseases and Injuries Collaborators, 2024). Depressive disorders are more common in women, particularly those of childbearing age (Sassarini, 2016; Gao et al., 2024). A previous study noted that the age-standardized DALY rate (ASDALYR) among middle-aged and older adults in China rose by 3.99%, with women consistently showing a higher ASDALYR than men (Xiao et al., 2025). The global incidence of depressive disorders among women has risen over the past 30 years, with the 15–49 age demographic representing the highest percentage of new cases (Gao et al., 2024). Research indicates that the prevalence of depressive disorders among women of childbearing age (WCBA) in rural China is 30.7 percent (Cao et al., 2015). A meta-analysis indicated that the prevalence of perinatal depression in low- and middle-income countries was 13.1%, highlighting a significant burden (Woody et al., 2017).

The probability of developing depressive disorders is nearly twice as high in women as in men, a disparity that persists throughout the reproductive years (Sassarini, 2016). Women experience distinct life stages, including menstruation, pregnancy, childbirth, and menopause, marked by substantial hormonal variations that increase their susceptibility to depressive disorders (Zeng et al., 2019). Depressive disorders in WCBA may manifest at various phases of the reproductive cycle, including premenstrual anxiety, maternal depression, and menopausal depression (Noble, 2005). Reproductive health events, such as oral contraceptive use, miscarriage, infertility, and hormone replacement therapy, have been shown to impact the mental health of WCBA and to induce depression (Noble, 2005; Montagnoli et al., 2020). Previous research indicated that depression during childbearing years is associated with adverse pregnancy outcomes, such as preterm birth, and low birth weight, as well as increased risk of severe maternal health complications and adverse maternal mortality (Miller et al., 2022; Masters et al., 2019; Nisar et al., 2020a). Moreover, economic disparities (such as economic discrimination and employment inequality) and the necessity for WCBA to undertake primary responsibilities for both household and childcare can result in role conflict and role overload, adversely impacting mental health (Piccinelli and Wilkinson, 2000). The social distancing restrictions and other psychosocial factors that intensified during the 2019 coronavirus disease pandemic contributed to a 27.6% global rise in depressive disorders (COVID-19 Mental Disorders Collaborators, 2021; Zhu et al., 2023). Therefore, it is essential to synthesize epidemiological indicators of depressive disorders among WCBA globally to inform targeted intervention strategies on an international level.

Multiple studies have investigated the incidence and trends of depressive disorders among Chinese women (Bao et al., 2024; Xiyuan and Chao, 2024; He et al., 2021), and a thorough evaluation of the global burden of depression in WCBA remains largely ambiguous or unreported. Considering that WCBA represent a key population group for family planning and maternal health, it is essential to conduct an in-depth analysis of the trends of depressive disorders among WCBA. Employing the latest data from GBD 2021, we performed an extensive analysis of the incidence, prevalence, and DALYs of depressive disorders among WCBA from 1990 to 2021, with projections extending to 2046.

## 2 Methods

### 2.1 Data source and disease definition

GBD 2021 offers the most recent epidemiological data analyzed for 371 diseases and injuries (GBD 2021 Diseases and Injuries Collaborators, 2024; GBD 2021 Risk Factors Collaborators, 2024), with all requisite data available for download from the Global Health Data Exchange (GHDx) query tool (https://vizhub.healthdata.org/gbd-results/). The dataset includes information on gender, age, incidence, prevalence, and DALY related to depressive disorders. Prior studies (Gao et al., 2024; Zhu et al., 2025; Feng et al., 2025) has delineated the pertinent statistical methodologies and principles, and this data resource is extensively utilized in the domain of epidemiology. Depressive disorders were classified into two categories based on the ICD-10 or criteria DSM-IV-TR (First, 2013; WHO, 1992), including major depressive disorders (ICD-10: F32.0–9, F33.0–9; DSM-IV-TR: 296.21–24, 296.31–34) and dysthymia (ICD-10: F34.1; DSM-IV-TR: 300.4). WHO defines WCBA as those aged 15 to 49 years. In this study, this demographic was categorized into seven age groups: 15–19, 20–24, 25–29, 30–34, 35–39, 40–44, and 45–49 years.

### 2.2 Socio-demographic index

The Socio-Demographic Index (SDI) is a composite metric of economic development, based on individual fertility rates, educational attainment, and per capita income (GBD 2021 Risk Factors Collaborators, 2024). The SDI ranges from 0 to 1, with higher values indicating increased economic levels. In GBD 2021, all countries were categorized into five classifications based on the SDI values: high SDI (>0.81), high-middle SDI (0.70–0.81), middle SDI (0.61–0.69), low-middle SDI (0.46–0.60), and low SDI (<0.46).

### 2.3 Statistical analysis

The disease burden of depressive disorders was primarily assessed via incidence, prevalence, and DALYs. DALYs are calculated as the aggregate of Years Lived with Disability (YLDs) and Years of Life Lost (YLLs) (GBD 2021 Diseases and Injuries Collaborators, 2024). This study calculated the age-standardized rate (ASR) of depressive disorders in WCBA, standardized per 100,000 WCBA patients, utilizing the following formula (Li et al., 2024a):


$$\begin{array}{l} \frac{\overset{N}{\underset{i=1}{\sum }}\alpha_{i}W_{i}}{\overset{N}{\underset{i=1}{\sum }}W_{i}} \end{array}$$

(α_*i*_: the age-specific rate for the *i*th age group, w: the number of individuals in the standard population corresponding to the *i*th age group, and *N*: the total number of people in the *i*th age group).

The definitions of age-standardized incidence rates (ASIRs), prevalence rates (ASPRs), and ASDALYRs remain aligned with methodologies utilized in prior GBD studies (Han et al., 2024; Jin et al., 2025). Consequently, we utilize the commonly employed estimated annual percentage change (EAPC) analysis to illustrate the trend of ASR variations over a designated timeframe. The formula is as follows (Yang et al., 2021):


$$\begin{array}{l} \;y=\alpha +\beta x+\varepsilon \;\\ EAPC=100\times \left(\exp\left(\beta \right)-1\right)\; \end{array}$$

(*x*: year, *y*: the natural logarithm of rates, α: the intercept, β: the slope, ε: the random error).

An EAPC and its 95% confidence interval (CI) above zero signify an increasing trend in ASR over time. Conversely, an EAPC and its 95% CI below zero indicate a decreasing trend in ASR. If the 95% CI encompasses zero, the change in ASR is deemed statistically insignificant. Furthermore, we employed percentage changes to illustrate variations in incidence, prevalence, and DALYs in 2021 relative to 1990.


$$\begin{array}{l} percentage\;change=\left(2021cases-1990cases\right)/1990cases \end{array}$$

Joinpoint regression is frequently employed to detect alterations in temporal trends. It accomplishes this by segmenting the regression, partitioning the longitudinal change into segments, identifying statistically significant segment trends, locating statistically significant inflection points, and computing the trend between them by fitting a linear model (Kim et al., 2000). For each sub-sector, we compute the annual percentage change (APC) to assess the extent and direction of variation. The average annual percentage change (AAPC) within the specified intervals was calculated by weighting the individual APCs according to their respective time frames, adhering to the established methodology (Cao et al., 2024). An upward trend is indicated when both the APC/AAPC estimates and the lower limit of their confidence intervals exceed 0; conversely, a decreasing trend is observed when both the APC/AAPC estimates and the upper limit of their confidence intervals fall below 0. In all other cases, the estimates are likely to stabilize (Qiu et al., 2021). We calculated the global ASIR, ASPR, and ASDALYR for the period 1990–2021 and assessed significance using Monte Carlo substitution.

Decomposition analysis is a statistical technique employed to investigate the temporal interactions of health-influencing factors (Das Gupta, 1994). This method is especially advantageous in epidemiology and public health, as it enables the measurement of how demographic, socioeconomic, and epidemiological factors influence changes in disease burden, thereby clarifying their underlying mechanisms (GBD 2019 Dementia Forecasting Collaborators, 2022). This study employed decomposition analysis to assess the influence of population growth, aging, and epidemiological changes on incidence, prevalence, and DALYs, thereby identifying the primary factors contributing to the alterations in the burden of depressive disorders from 1990 to 2021. We used total population size and annual growth rate for population growth, age-specific incidence/prevalence rates for aging, and ASRs over time for epidemiological changes.

To ascertain trends of depressive disorders among WCBA by 2046, the number of cases, ASIR, ASPR, and ASDALYR for depressive disorders among WCBA were forecasted utilizing the Nordpred package in R. The Nordred method is a predictive instrument grounded in the Age-Period-Cohort model, which integrates the robust functionalities of the generalized linear model (GLM) (Xu and Hertzberg, 2013). It can model data using the GLM framework to precisely assess the influence of age, period, and cohort on epidemiological metrics (Li et al., 2024b). This approach provides a robust framework for estimating health scenarios based on historical patterns and demographic changes. All analyses were performed using R software (version 4.4.1).

## 3 Results

### 3.1 Global level

**Figure 1:**
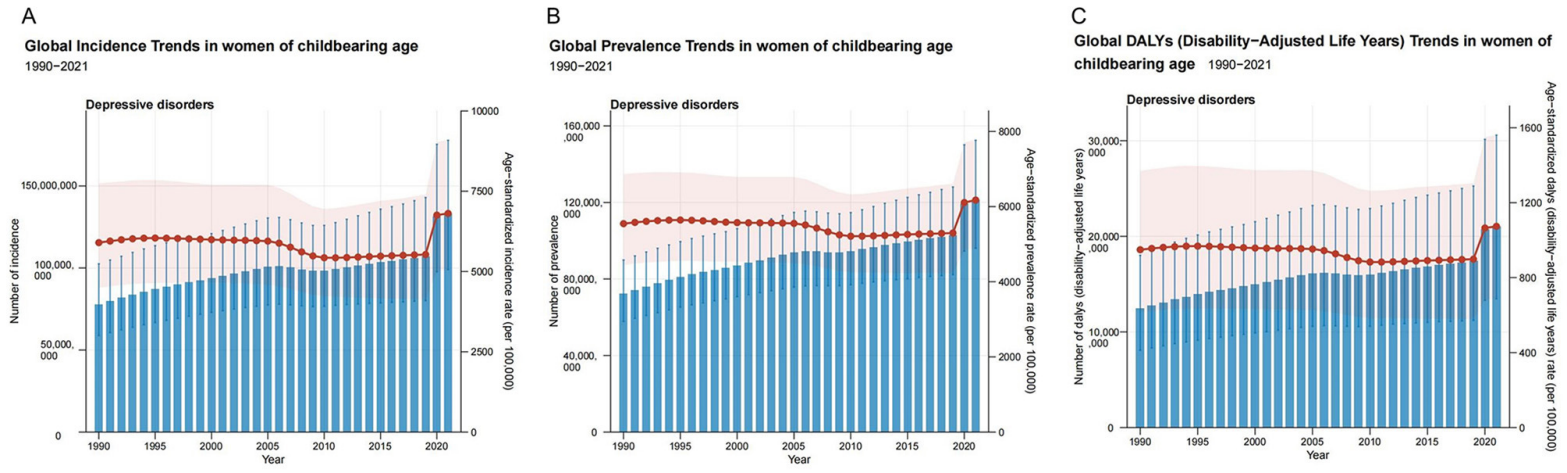
The trends in numbers and rates of incidence **(A)**, prevalence **(B)**, and DALYs **(C)** for depressive disorders among WCBA in global from 1990 to 2021; DALYs, disability-adjusted life years; WCBA, women of childbearing age. Blue bars represent the absolute number and 95% uncertainty interval (UI) for incidence, prevalence, and DALYs; Red points represent the age-standardized rate per 100,000 for each year; Light red shaded area represents the 95% UI for the age-standardized rate.

In 2021, the global incidence of depressive disorders in WCBA increased from 77.72 (95% UI: 58.86 to 102.54) million in 1990 to 133.25 (95% UI: 99.03 to 177.88) million, representing a 71% increase (Table 1, Figure 1A, and Supplementary Tables S1, S2). The prevalence number of WCBA increased from 72.35 (95% UI: 57.79 to 89.93) million in 1990 to (95% UI: 96.05 to 152.52) 121.24 million in 2021, representing an increase of 68% (Table 1, Figure 1B, and Supplementary Tables S1, S2). Concurrently, DALY increased from 12.45 (95% UI: 8.08 to 18.02) million in 1990 to 21.04 (95% UI: 13.47 to 30.59) million in 2021, representing an increase of 69% (Table 1, Figure 1C, and Supplementary Tables S1, S2). Furthermore, the global ASIR, ASPR and ASDALYR in 2021 were 6,808.01 (95% UI: 5,049.99 to 9,106.66) per 100,000, 6,173.45 (95% UI: 4,883.27 to 7,781.73) per 100,000 and 1,073.50 (95% UI: 686.73 to 1,562.48) per 100,000 (Table 1).

Table 1The incidence, prevalence, and DALYs of depressive disorders cases and rates among WCBA in 2021, with EAPC from 1990 to 2021.

**Table:** 

**Location**	**Incidence Cases, million (95% UI)**	**ASIR, per 100,000 (95% UI)**	**EAPC (95% CI)**	**Prevalence Cases, million (95% UI)**	**ASPR, per 100,000 (95% UI)**	**EAPC (95% CI)**
Global	133.25 (99.03 to 177.88)	6,808.01 (5,049.99 to 9,106.66)	−0.18 (−0.39 to 0.04)	121.24 (96.05 to 152.52)	6,173.45 (4,883.27 to 7,781.73)	−0.12 (−0.27 to 0.03)
Low SDI	198.01 (138.9 to 274.43)	7,509.35 (5,325.8 to 10,306.14)	−0.36 (−0.53 to −0.19)	17.91 (13.61 to 23.07)	6,848.6 (5,241.12 to 8,747.39)	−0.25 (−0.37 to −0.13)
Low-middle SDI	37.75 (27.47 to 51.29)	7,551.4 (5,510.78 to 10,222.82)	−0.60 (−0.86 to −0.34)	33.06 (25.81 to 42.32)	6,628.44 (5,185.01 to 8,458.5)	−0.42 (−0.6 to −0.23)
Middle SDI	36.08 (26.89 to 47.66)	5,767.56 (4,281.65 to 7,652.92)	−0.25 (−0.48 to −0.01)	34.25 (27.29 to 42.58)	5,421.62 (4,305.2 to 6,772.18)	−0.19 (−0.35 to −0.03)
High-middle SDI	17.86 (13.12 to 23.82)	5,778.86 (4,201.28 to 7,786.22)	−0.33 (−0.57 to −0.09)	17.38 (13.80 to 21.68)	5,475.98 (4,303.49 to 6,906.98)	−0.24 (−0.41 to −0.07)
High SDI	21.66 (16.62 to 28.11)	9,099.46 (6,930.43 to 11,859.63)	0.54 (0.31 to 0.77)	18.55 (14.88 to 22.98)	7,677.86 (6,120.77 to 9,579.04)	0.36 (0.18 to 0.53)
Andean Latin America	1.11 (0.75 to 1.59)	6,320.57 (4,292.05 to 9,087.82)	0.06 (−0.33 to 0.45)	0.94 (0.69 to 1.26)	5,361.92 (3,933.81 to 7,199.44)	0.05 (−0.24 to 0.34)
Australasia	0.72 (0.50 to 1.01)	10,269.64 (7,057.12 to 14,369.31)	0.24 (0.08 to 0.41)	0.60 (0.45 to 0.80)	8,462.14 (6,257.29 to 11,300.83)	0.20 (0.07 to 0.33)
Caribbean	0.97 (0.66 to 1.39)	8,029.7 (5,444.45 to 11,483.95)	−0.50 (−0.76 to −0.24)	0.79 (0.57 to 1.07)	6,502.68 (4,706.8 to 8,863.19)	−0.42 (−0.63 to −0.21)
Central Asia	1.37 (0.95 to 1.92)	5,623.36 (3,891.23 to 7,918.02)	0.10 (−0.09 to 0.3)	1.28 (0.97 to 1.68)	5,231.16 (3,920.92 to 6,874.97)	0.07 (−0.06 to 0.21)
Central Europe	1.27 (0.91 to 1.73)	4,795.85 (3,393.82 to 6,608.56)	−0.47 (−0.8 to −0.15)	1.27 (0.99 to 1.62)	4,677.25 (3,608.8 to 6,022.43)	−0.30 (−0.50 to −0.09)
Central Latin America	5.40 (3.92 to 7.30)	7,882.55 (5,720.17 to 10,666.28)	1.02 (0.80 to 1.24)	4.31 (3.29 to 5.58)	6,293.78 (4,790.95 to 8,147.72)	0.80 (0.62 to 0.98)
Central Sub-Saharan Africa	3.58 (2.37 to 5.21)	11,270.02 (7,523.17 to 16,274.56)	−0.02 (−0.13 to 0.1)	2.95 (2.12 to 4.04)	9,386.69 (6,800.91 to 12,768.75)	−0.01 (−0.1 to 0.07)
East Asia	10.40 (7.91 to 13.37)	2,956.12 (2,227.57 to 3,840.52)	−1.40 (−1.65 to −1.16)	13.47 (10.98 to 16.34)	3,700 (2,994.89 to 4,515.91)	−0.93 (−1.08 to −0.78)
Eastern Europe	3.50 (2.50 to 4.76)	6,990.81 (4,956.63 to 9,586.01)	−0.13 (−0.41 to 0.15)	3.12 (2.44 to 3.95)	6,116.21 (4,742.5 to 7,829.81)	−0.08 (−0.28 to 0.12)
Eastern Sub-Saharan Africa	8.19 (5.71 to 11.44)	7,985.77 (5,630.58 to 11,024.96)	−0.27 (−0.43 to −0.12)	7.58 (5.75 to 9.82)	7,444.18 (5,696.51 to 9,554.62)	−0.21 (−0.31 to −0.10)
High-income Asia Pacific	1.83 (1.39 to 2.37)	5,010.7 (3,763.15 to 6,511.04)	0.47 (0.27 to 0.66)	1.61 (1.29 to 1.98)	4,293.05 (3,428.65 to 5,331.89)	0.27 (0.11 to 0.44)
High-income North America	10.48 (8.14 to 13.30)	12,689.95 (9,828.58 to 16,139.08)	0.82 (0.47 to 1.16)	8.70 (7.05 to 10.66)	10,443.59 (8,434.35 to 12,835.72)	0.49 (0.26 to 0.71)
North Africa and Middle East	16.02 (10.98 to 22.83)	10,032.79 (6,876.71 to 14,305.05)	0.20 (0.04 to 0.36)	13.30 (9.81 to 17.76)	8,318.57 (6,135.59 to 11,121.61)	0.17 (0.05 to 0.30)
Oceania	0.14 (0.09 to 0.21)	4,125.32 (2,738.85 to 6,006.85)	−0.13 (−0.19 to −0.06)	0.15 (0.11 to 0.21)	4,495.59 (3,337.06 to 5,970.94)	−0.08 (−0.12 to −0.04)
South Asia	35.58 (26.42 to 47.28)	7,283.76 (5,421.29 to 9,654.43)	−0.99 (−1.31 to −0.66)	31.20 (24.62 to 39.27)	6,390.39 (5,049.98 to 8,025.78)	−0.70 (−0.93 to −0.46)
Southeast Asia	7.02 (5.01 to 9.56)	3,829.96 (2,729.29 to 5,227.5)	−0.04 (−0.26 to 0.17)	7.96 (6.24 to 10.10)	4,294.28 (3,358.87 to 5,457.74)	−0.02 (−0.13 to 0.1)
Southern Latin America	1.40 (1.01 to 1.90)	8,133.97 (5,867.86 to 11,029.02)	−0.17 (−0.43 to 0.08)	1.13 (0.85 to 1.47)	6,509.67 (4,896.55 to 8,513.81)	−0.22 (−0.45 to 0.01)
Southern Sub-Saharan Africa	1.89 (1.40 to 2.51)	8,722.61 (6,487.11 to 11,549.81)	0.37 (0.09 to 0.64)	1.66 (1.31 to 2.09)	7,672.13 (6,067.56 to 9,620.79)	0.26 (0.07 to 0.46)
Tropical Latin America	6.00 (4.49 to 7.86)	9,756.89 (7,276.88 to 12,821.9)	−0.44 (−0.82 to −0.07)	4.73 (3.69 to 5.95)	7,633.97 (5,948.15 to 9,648.14)	−0.37 (−0.68 to −0.05)
Western Europe	9.42 (6.93 to 12.71)	10,094.71 (7,338.27 to 13,758.87)	0.20 (−0.01 to 0.42)	7.76 (6.00 to 9.98)	8,254.89 (6,326.11 to 10,733.02)	0.16 (−0.01 to 0.33)
Western Sub-Saharan Africa	6.97 (4.93 to 9.75)	6,114.54 (4,369.47 to 8,446.05)	−0.33 (−0.44 to −0.21)	6.74 (5.17 to 8.68)	5,969.48 (4,614.72 to 7,618.2)	−0.21 (−0.29 to −0.14)

**Table:** 

**Location**	**DALYs, million (95%UI)**	**ASDALYR, per 100,000 (95% UI)**	**EAPC (95% CI)**
Global	21.04 (13.47 to 30.59)	1,073.50 (686.73 to 1,562.48)	−0.14 (−0.32 to 0.04)
Low SDI	3.10 (1.94 to 4.60)	1,179.58 (740.73 to 1,739.54)	−0.27 (−0.41 to −0.13)
Low-middle SDI	5.82 (3.68 to 8.53)	1,164.37 (735.95 to 1,704.52)	−0.49 (−0.71 to −0.27)
Middle SDI	5.82 (3.72 to 8.43)	926.08 (591.77 to 1,344.73)	−0.22 (−0.42 to −0.03)
High-middle SDI	2.94 (1.88 to 4.28)	939.53 (596.4 to 1,375.97)	−0.28 (−0.48 to −0.08)
High SDI	3.35 (2.21 to 4.82)	1,399.31 (921.2 to 2,022.31)	0.44 (0.25 to 0.64)
Andean Latin America	0.17 (0.10 to 0.26)	966.63 (579.65 to 1,490.12)	0.06 (−0.28 to 0.39)
Australasia	0.11 (0.07 to 0.17)	1,563.09 (962.3 to 2,373.22)	0.24 (0.09 to 0.38)
Caribbean	0.15 (0.09 to 0.22)	1,195.95 (719.21 to 1,842.41)	−0.48 (−0.72 to −0.25)
Central Asia	0.22 (0.14 to 0.33)	903.45 (561.45 to 1,363.66)	0.10 (−0.06 to 0.26)
Central Europe	0.21 (0.13 to 0.31)	788.38 (492.87 to 1,163.38)	−0.38 (−0.64 to −0.12)
Central Latin America	0.80 (0.50 to 1.19)	1,168.22 (724.13 to 1,736.5)	0.91 (0.71 to 1.11)
Central Sub-Saharan Africa	0.54 (0.31 to 0.83)	1,695.2 (994.94 to 2,610.73)	0.02 (−0.07 to 0.12)
East Asia	2.01 (1.32 to 2.85)	560.51 (365.8 to 799.17)	−1.15 (−1.34 to −0.95)
Eastern Europe	0.55 (0.35 to 0.81)	1,083.11 (684.77 to 1,608.23)	−0.09 (−0.33 to 0.15)
Eastern Sub-Saharan Africa	1.30 (0.80 to 1.95)	1,273.15 (789.45 to 1,889.91)	−0.21 (−0.33 to −0.08)
High-income Asia Pacific	0.29 (0.19 to 0.42)	783.79 (508.07 to 1,134.18)	0.38 (0.20 to 0.56)
High-income North America	1.60 (1.06 to 2.29)	1,929.21 (1,284.02 to 2,772.95)	0.64 (0.36 to 0.92)
North Africa and Middle East	2.42 (1.47 to 3.67)	1,515.15 (916.91 to 2,301.13)	0.19 (0.05 to 0.34)
Oceania	0.03 (0.02 to 0.04)	720.59 (430.31 to 1,107.37)	−0.09 (−0.14 to −0.04)
South Asia	5.46 (3.48 to 7.95)	1,118.19 (713.21 to 1,625.21)	−0.82 (−1.10 to −0.54)
Southeast Asia	1.26 (0.80 to 1.86)	683.77 (434.95 to 1,007.98)	−0.01 (−0.17 to 0.15)
Southern Latin America	0.21 (0.13 to 0.31)	1,221.46 (760.65 to 1,814.09)	−0.20 (−0.44 to 0.05)
Southern Sub-Saharan Africa	0.29 (0.19 to 0.42)	1,329.3 (856.5 to 1,943.43)	0.28 (0.04 to 0.52)
Tropical Latin America	0.88 (0.56 to 1.28)	1,419.51 (900.1 to 2,073.83)	−0.41 (−0.75 to −0.06)
Western Europe	1.43 (0.91 to 2.11)	1,527.48 (969.93 to 2,272.7)	0.18 (−0.02 to 0.37)
Western Sub-Saharan Africa	1.13 (0.71 to 1.68)	996.09 (626.15 to 1,467.43)	−0.24 (−0.33 to −0.14)

ASIR, age-standardized incidence rate; ASPR, age-standardized prevalence rate; ASDALYR, age-standardized disability-adjusted life years rate; DALYs, age-standardized disability-adjusted life years; EAPC, estimated annual percentage change; WCBA, women of childbearing age.

### 3.2 SDI regional level

**Figure 2:**
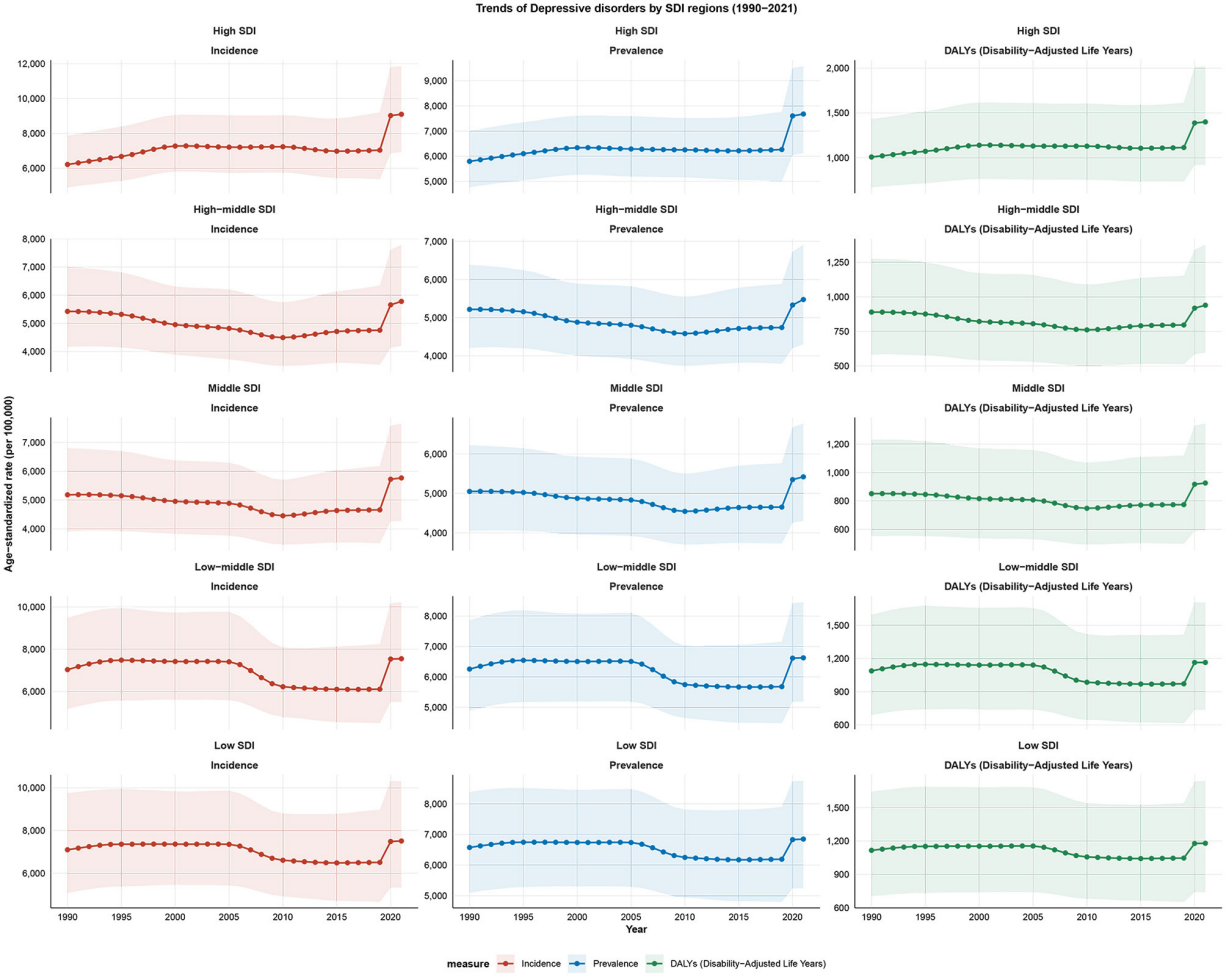
Trends of depressive disorders among WCBA by SDI regions from 1990 to 2021; SDI, socio-demographic index; WCBA, women of childbearing age.

In 2021, the number of incident cases was highest in the low-middle SDI regions at 37.75 million. In the same year, the numbers of prevalent cases and DALYs were highest in the Middle SDI regions at 34.25 million and 5.82 million, respectively. In 2021, the highest ASIR, ASPR and ASDALYR were observed in the High SDI regions, at 9,099.46 per 100,000, 7,677.86 per 100,000 and 1,399.31 per 100,000 (Table 1). The percentage change in incidence, prevalence and DALY was highest in low SDI region, at ~162%, while the percentage change was lowest in High-middle SDI, at approximately 20% (Supplementary Table S2). From 1990 to 2021, High SDI regions were the only ones showing an upward trend in ASIR, ASPR, and ASDALYR, with EAPCs of 0.54 (95% CI: 0.31 to 0.77), 0.36 (95% CI: 0.18 to 0.53), and 0.44 (95% CI: 0.25 to 0.64), respectively (Table 1, Figure 2, Supplementary Figure S1).

### 3.3 GBD regional level

In 2021, South Asia accounted for the highest number of incident cases, prevalent cases, and DALYs among the 21 GBD regions, with 35.58 million, 31.20 million, and 5.46 million, respectively. In contrast, High-income North America exhibited the highest ASIR, ASPR and ASDALYR of 12,689.95 per 100,000, 10,443.59 per 100,000, and 1,929.21 per 100,000 (Table 1, Supplementary Figure S2). The incidence numbers exhibited an upward trend in all regions except East Asia, which demonstrated a negative change of −29%. The most significant increase was observed in Central Sub-Saharan Africa, reaching ~181%. Central Europe and East Asia demonstrated a negative change in both prevalence and DALY numbers, while the other regions exhibited an upward trend in both (Supplementary Table S2). It is noteworthy that Central Sub-Saharan Africa also experienced the most significant increase in both prevalence and DALY numbers, with increases of 178 and 182%, respectively (Table 1, Supplementary Figure S2, and Supplementary Table S2).

### 3.4 National level

**Figure 3:**
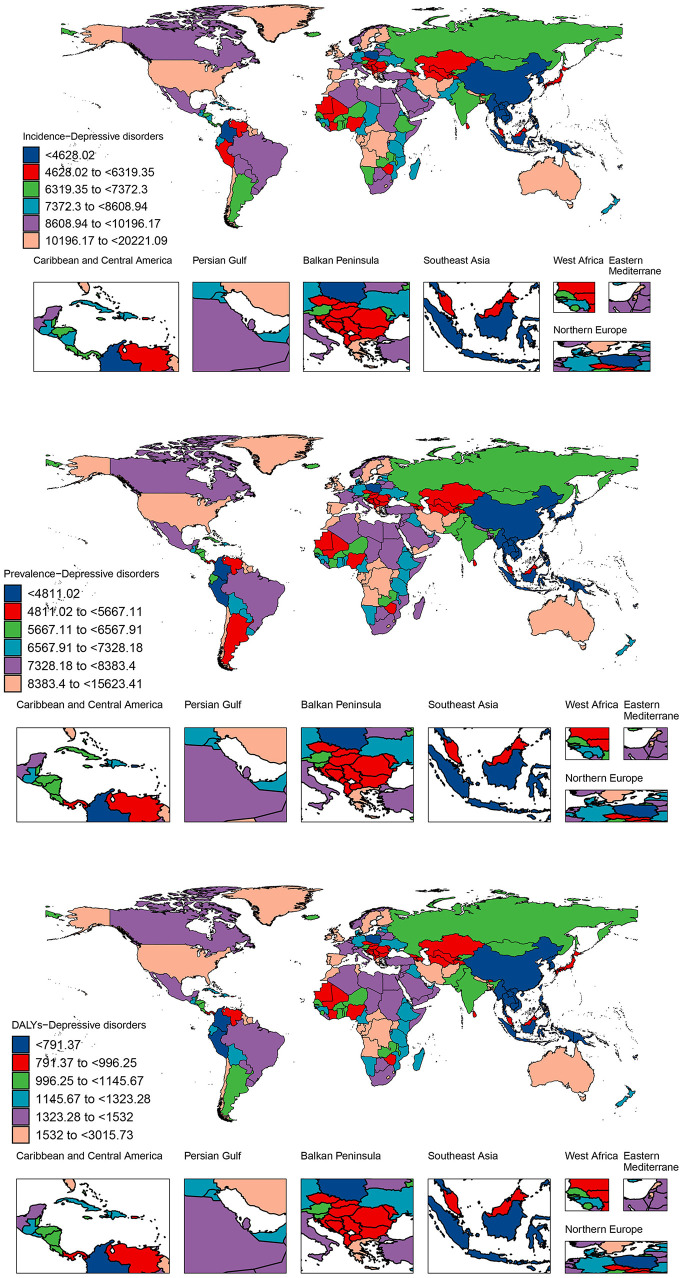
Incidence, prevalence, and DALYs for depressive disorders among WCBA across 204 countries and territories in 2021; DALYs, disability-adjusted life years; WCBA, women of childbearing age.

From 1990 to 2021, ~88% of countries showed an increasing trend in the cases of incidence, prevalence, and DALYs of depressive disorders among WCBA. Qatar, United Arab Emirates, and Jordan had the highest percentage changes, ranging from 299 to 634%. In addition, only 12% of countries showed a reduction in cases of indicators, such as Bosnia and Herzegovina and Cuba (Supplementary Table S3). At the national level, the number of incident cases in India increased from 13.37 million in 1990 to 25.59 million in 2021, making it the country with the highest number of cases. It was followed by China with 9.99 million cases and the USA with 9.74 million cases in 2021. In 2021, Greenland had the highest ASIR at 20,221.09 per 100,000, followed by Lesotho and Greece with 13,845.53 per 100,000 and 13,841.31 per 100,000 respectively (Figure 3, Supplementary Table S4). About one third of countries showed an increasing trend in the incidence of WCBA depression, with the largest increase in Mexico, which had an EAPC of 1.86 (95% CI: 1.56 to 2.17). The largest declining in incidence occurred in Singapore, with an EAPC of −2.20 (−2.57 to −1.83) (Supplementary Table S4).

In addition, the top three countries with the highest numbers of prevalence and DALYs of depression among WCBA were the same as the top three countries with the highest numbers of incidence. Specifically, India had the highest number of prevalent cases and DALYs, with 22.84 (95% UI: 18.11 to 28.49) million and 3.96 (95% UI: 2.54 to 5.76) million, respectively. Subsequently, China had prevalence numbers of 12.98 (95% UI: 10.60 to 15.72) million and DALYs of 1.94 (95% UI: 1.27 to 2.74) million. In third place was the United States, with prevalence numbers of 8.04 (95% UI: 6.53 to 9.81) million and DALYs of 1.48 (95% UI: 0.99 to 2.12) million (Figure 3, Supplementary Tables S5, S6). Greenland had the highest ASPR and ASDALYR at 15,623.41 and 3,015.73 per 100 000, respectively. In ASPR, Lesotho ranked second with 11,151.88 per 100,000, followed by Greece with 10,839 per 100,000. For ASDALYR, on the other hand, Greece came second with 2,062.84 per 100,000 and Lesotho third with 2,030.26 per 100,000. From 1990 to 2021, Mexico had the fastest growing ASPR and ASDALYR with EAPCs of 1.49 (1.23 to 1.74) and 1.69 (1.41 to 1.96), while Singapore had the fastest declining ASPR and ASDALYR with EAPCs of −1.85 (−2.16 to −1.54) and −2.05 (−2.40 to −1.71) (Figure 3, Supplementary Tables S5, S6).

### 3.5 Joinpoint regression analysis

**Figure 4:**
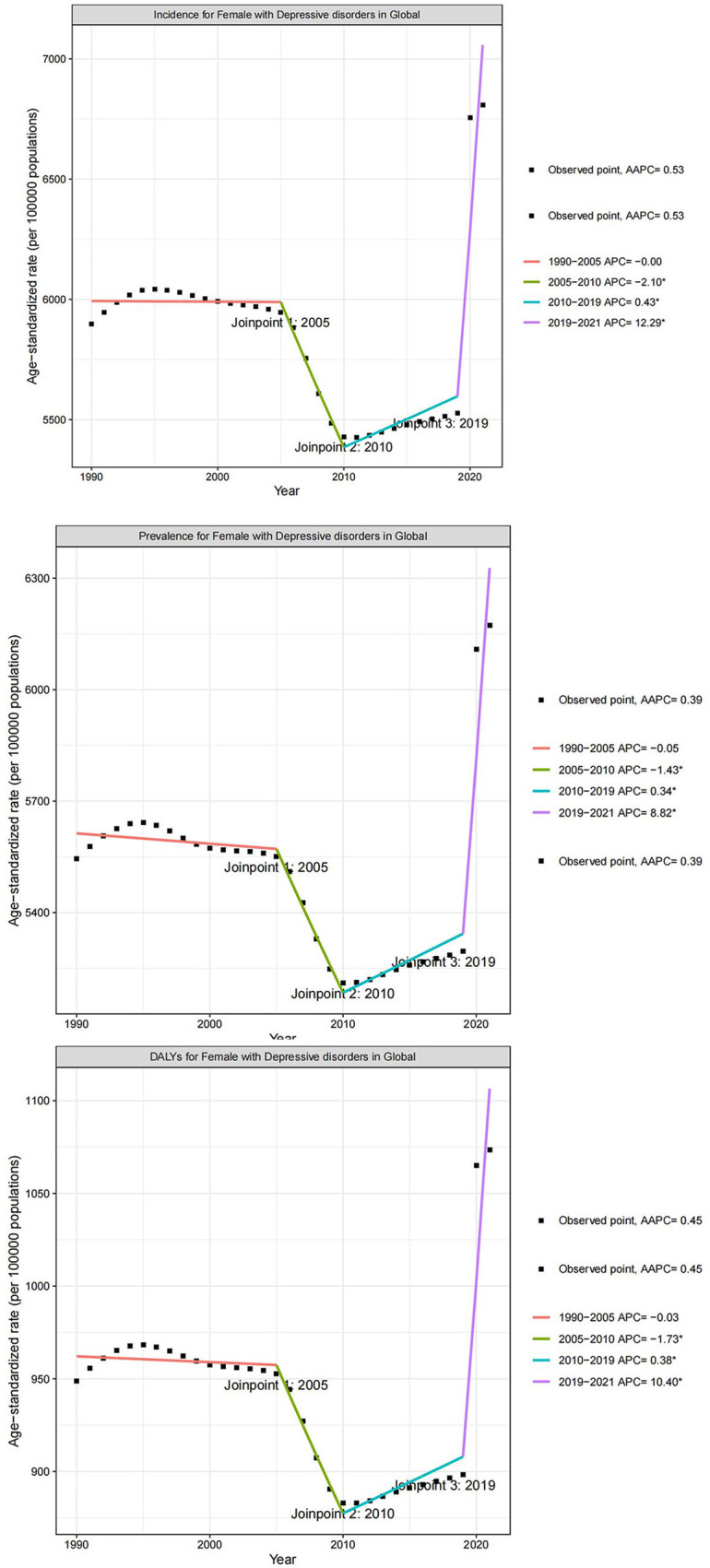
Joinpoint regression analysis of the temporal trends of depressive disorders among WCBA from 1990 to 2021; WCBA, women of childbearing age.

Joinpoint regression analyses showed a general increase in ASIR (AAPC: 0.53; 95% CI: 0.40 to 0.71), ASPR (AAPC: 0.39; 95% CI: 0.31 to 0.51), and ASDALYR (AAPC: 0.45; 95% CI: 0.35 to 0.61) for depressive disorders among WCBA. We found that ASIR showed a decreasing trend from 2005–2009 (APC: −2.10; 95% CI: −3.73 to −1.11), but increased overall since 2010. Specifically, ASIR increased slowly from 2010–2018 (APC: 0.43; 95% CI: 0.06 to 1.00) and sharply after 2019 (APC: 12.29; 95% CI: 7.97 to 15.65), and these changes were statistically significant (*p* < 0.001). Similarly, ASPR decreased significantly from 2005 to 2009 (APC: −1.43; 95% CI: −2.52 to −0.75), increased slightly after 2010 (APC: 0.34; 95% CI: 0.09 to 0.70), and the rate of increase accelerated after 2019 (APC: 8.82; 95% CI: 5.77 to 11.03) and was statistically significant (*p* < 0.001). ASDALYR only showed a decreasing trend from 2005–2009 (APC: −1.73; 95% CI: −3.07 to −0.90), a small increase from 2010–2018 (APC: 0.38; 95% CI: 0.07 to 0.85) and a significant increase after 2019 (APC: 10.40; 95% CI: 6.75 to 13.18) and was statistically significant (*p* < 0.001) (Figure 4).

### 3.6 Decomposition analysis

**Figure 5:**
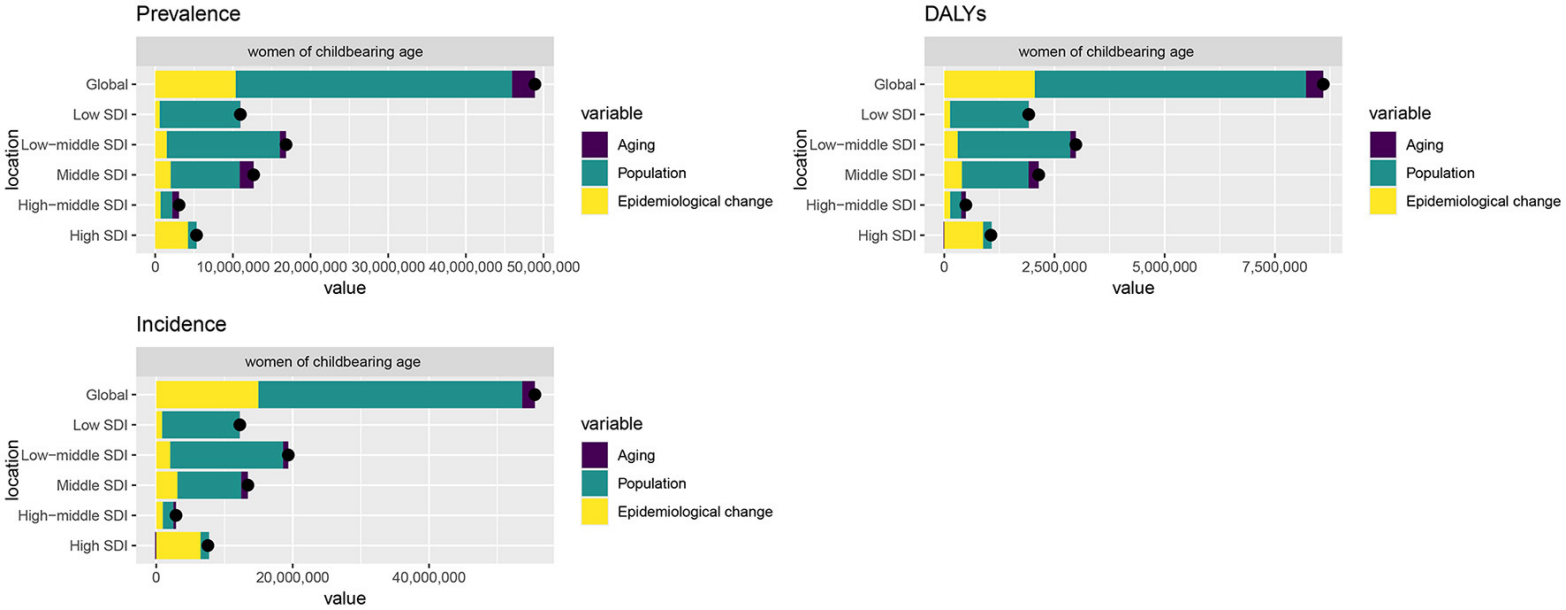
Decomposition analysis of incidence, prevalence, and DALYs change in depressive disorders among WCBA from 1990 to 2021 at the global level and by SDI; DALYs, disability-adjusted life years; WCBA, women of childbearing age.

The contribution of population growth, epidemiological changes and aging to the incidence, prevalence and DALYs of depression in WCBA varied globally from 1990 to 2021. For incidence, population growth contributed the most (69.67%), followed by epidemiological changes (26.94%) and aging (3.39%). For prevalence, population growth contributed 72.76%, epidemiological changes 21.17% and aging 6.07%. For DALYs, the contributions of population growth, epidemiological changes and aging were 71.53, 23.89 and 4.59%, respectively. From the regional perspective of SDI, the main driver of prevalence in high SDI areas was epidemiological change (85.77%), whereas the main driver of growth in High-middle SDI (53.33%), Middle SDI (69.53%), Low-middle SDI (85.58%) and Low SDI (92.83%) was population growth. The pattern of prevalence and DALYs was highly consistent with this (Figure 5).

### 3.7 Age patterns

In 2021, the number of incident cases of depressive disorders among WCBA peaked at 20.44 million in the 35–39 age group. This was closely followed by the 30–34 and 20–24 age groups, with 19.96 million and 19.52 million cases, respectively. Globally, the number of prevalent cases also peaked in the 35–39 age group at 19.28 million, followed by the 30–34 age group with 18.74 million cases and the 40–44 age group with 18.42 million cases. Globally, the cases of DALYs due to depressive disorders was highest in the 35–39 age group. In 2021, the global incidence rate of WCBA depression started to increase from the 15–19 age group and peaked at 7,742.02 in the 40–44 age group. The prevalence rate increased with age and peaked in the 45–49 age group at 7,529.49. The rate of DALYs was highest in the 45–49 age group at 1,248.37 (Supplementary Figures S3, S4).

### 3.8 Predictions of burden of depressive disorders among WCBA for 2046

**Figure 6:**
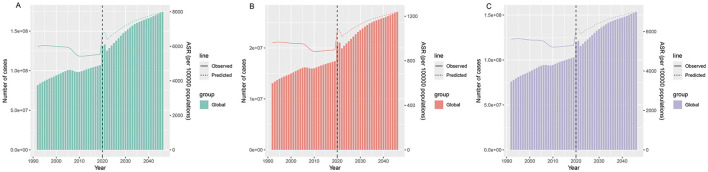
Prediction of incidence **(A)**, DALYs **(B)**, and prevalence **(C)** for depressive disorders among WCBA in global by 2046; DALYs, disability-adjusted life years; WCBA, women of childbearing age.

We used Nordpred model to predict the burden of depressive disorders in WCBA from 2021 to 2046, and the results showed that the ASIR, ASPR and ASDALYR of depressive disorders in WCBA showed an increasing trend. The global ASIR for the WCBA population is projected to increase from 6,808.01 per 100,000 in 2021 to 7,991.13 per 100,000 in 2046. The ASPR will peak in 2046. It is projected to increase to 6,995.70 per 100 000 in 2046. ASDLYR is projected to increase from 1,073.5 per 100 000 in 2021 to 1,238.43 per 100 000 in 2046 (Figure 6).

## 4 Discussion

In this study, to the best of our knowledge, we reported the incidence, prevalence, and DALY burden of depression in WCBA at global, regional, and national levels using the GBD 2021 and projected the burden of depression in this population in 2046. Key findings include: first, the global prevalent cases of depressive disorders in WCBA showed an increasing trend since 1990. Second, there was significant regional variation in depressive disorders among WCBA. At the SDI level, high SDI region had the highest ASIR, ASPR, and ASDALYR. Regarding 21 GBD regions, most regions showed an upward trend in ASIR. Among them, high-income North America had the highest ASIR, ASPR, and ASDALYR. At the country level, the country with the highest ASIR, ASPR, and ASDALYR was Greenland. Third, joinpoint regression analysis showed a sharp increase in ASIR for WCBA depression after 2019. Finally, we found that globally, the highest incidence of WCBA was found in the 40–44 age group, which may be related to hormonal fluctuations, as this is when women enter perimenopause and menopause (Gyllstrom et al., 2007).

The current study indicated that the global ASIR for depressive disorders among WCBA has risen over the past three decades, underscoring the necessity for increased focus on age-specific populations affected by depression. Research indicates that adolescent depression impacts fewer than half as many males as females (Bouma et al., 2008). Sex hormones are crucial in their function. The dysregulation of the HPA axis, a principal stress management system in the body, indicates vulnerability to depression (Burke et al., 2005). Prior research has demonstrated that the HPA axis is markedly affected by female sex hormones, particularly estrogen and progesterone (Bangasser et al., 2010). Conversely, sex hormones induce alterations in body shape in girls during puberty, and heightened self-awareness amplified by modern social contexts such as social media comparisons renders them more susceptible to adverse assessments of these changes, thereby elevating the risk of depression (Woods and Scott, 2016; Ivie et al., 2020). Cyranowski et al.'s model posits that adolescent girls exhibit heightened susceptibility to interpersonal stress. As the demand for gendered role conformity (e.g., subordination) escalates during puberty, their sensitivity to interpersonal dynamics, such as evaluative scrutiny from peers, intensifies (Cyranowski et al., 2000). This susceptibility is further shaped by structural factors: Kuehner noted that macro-level gender inequity, including limited access to resources and societal gender discrimination, exacerbates women's exposure to stressors and their vulnerability to depression (Kuehner, 2017).

Depressive disorders is a significant occurrence for women, particularly during pregnancy. A study revealed that the prevalence of maternal depression was 26.3% (Worku et al., 2025), with its elevated occurrence linked to both general psychosocial factors (e.g., domestic violence, unemployment, life stress) and pregnancy-specific factors. Research indicated that exposure to spousal violence during pregnancy increases the risk of maternal depression threefold (Howard et al., 2013). A study involving nearly 200 pregnant women revealed that smoking and the consumption of sleeping pills, analgesics, and antiemetics were significantly associated with an increased risk of depression during pregnancy (Newport et al., 2012). Additionally, several physical and physiological determinants specific to pregnancy are associated with this risk. A systematic review of 20 studies examined the correlation between depression during pregnancy and maternal sleep deprivation, dysregulated HPA function, and disturbances in the immune-inflammatory response (Palagini et al., 2014), which warrants attention from researchers. Cortisol, a hormone linked to stress, is significantly correlated with stress levels. A study of blood samples from 105 pregnant women revealed a negative correlation between depressive symptoms and pro-inflammatory cytokines (e.g., IL-1β, TNF, and IL-7), as well as a negative association between cortisol and these inflammatory factors (Field, 2017). This indicates that an immune system imbalance may, to a degree, be associated with an increased risk of depression in maternal women.

WCBA depressive disorders were more prevalent in higher-economic regions, with the highest ASIR in High SDI regions and it is the only SDI region with an upward trend. This pattern can be explained through a bio-social interaction framework that connects social, structural, and biological factors specific to high-SDI contexts. In high-SDI regions, WCBA face elevated career expectations driven by advances in education and gender equality awareness (Aulia, 2024), yet they still bear disproportionate caregiving burdens; this mismatch between heightened role demands and persistent traditional responsibilities creates social role strain, a key source of chronic stress. Additionally, the abundant medical resources (Jorm et al., 2017; Richardson et al., 2024) and rapid spread of mental health information high-SDI regions (Fan et al., 2025a) enhance detection and diagnosis of depressive symptoms. Moreover, documented heightened reactivity of WCBA to stress interacts with these contextual pressures: in high-SDI settings where self-esteem is closely tied to role fulfillment (Kim and Moore, 2019; Wang et al., 2025), stress from unmet expectations triggers self-critical responses that further increase depression risk (Hyde and Mezulis, 2020). Our findings also indicated that the percentage change in depression cases is the largest in low SDI regions, which may be associated with limited mental health services (Lopes et al., 2016) and economic instability. Studies have found that women are more vulnerable to the pandemic than men due to lower wages, job loss and caregiving burdens (Arcand et al., 2023), which may contribute to a higher incidence of depression. This contrast, which involves highest ASIR in high SDI regions alongside the steepest case growth in low SDI regions, highlights a critical dynamic in global mental health disparities. This disparity stems from unequal resource access: low SDI regions, with underfunded and fragmented mental health systems, cannot keep pace with rising burdens, while high SDI regions leverage stronger infrastructure to mitigate risks. Such gaps, often widened by crises like the pandemic, entrench health inequities by leaving the most affected populations with the least support (De Sousa et al., 2020; Abas et al., 2021).

Our research indicated that, among the 21 GBD regions, High-income North America exhibited the highest ASIR burden of WCBA depressive disorders. This may pertain to socioeconomic status and life stressors. Greenland holds the top position in the ASIR at the national level. The nation is sparsely populated, and the WCBA demographic has limited opportunities for social interaction, resulting in a markedly elevated risk of depressive disorders among WCBA (Ingemann et al., 2021). Moreover, the island's severe working conditions, occupational uniformity, and limited labor force are substantial risk factors for depression among the WCBA population (Christiansen et al., 2021). The severe living conditions and perpetual darkness significantly intensify the psychological distress of WCBA, which indirectly leads to an increase in ASIR (Timlin et al., 2021).

Our Joinpoint regression analyses revealed a significant reversal and marked increase in the ASIR for depressive disorders among WCBA post-2019. Prior research indicates that the worldwide incidence of depressive disorders significantly rose during the initial year of the COVID-19 pandemic (Hawes et al., 2022). The increased ASIR for depression in the WCBA resulted from a multifactorial combination of elements. The COVID-19 pandemic resulted in a deterioration of socioeconomic performance, with women of childbearing age experiencing higher unemployment rates, and unstable household income exacerbating psychological distress (Montenovo et al., 2022). Conversely, marital and familial status constituted a significant factor: numerous WCBA experienced the loss of relatives or spouses during the epidemic, intensifying their distress. Moreover, stringent social distancing measures during the epidemic diminished the social networks of WCBA, heightening feelings of isolation and the likelihood of depressive disorders. Health apprehensions regarding the novel coronavirus and anxieties surrounding vaccine safety were significant contributors to the increased ASIR for depression among WCBA (Jiang et al., 2025). In the decomposition analysis, swift population growth significantly impacts WCBA, resulting in a rise in the number of WCBA populations, which subsequently elevates the absolute counts of prevalence and DALYs. However, this does not indicate worsening epidemiology, as the ASRs showed a slight decline. The epidemiological proportion ranks second highest, signifying considerable potential for enhancement in public health. In the future, to effectively manage the incidence, prevalence, and DALYs of depressive disorders among WCBA, it is essential to strategically allocate public health resources and regulate epidemiological trends within a large population. However, decomposition analysis has its limitations. It isolates factors, missing interactive effects like how urbanization-linked population surges might strain mental health resources. Aggregated data may conceal subgroup differences. Unmeasured confounders, such as regional mental health service disparities, could also affect results. Critically, focusing on population growth doesn't negate the importance of addressing epidemiological drivers like social stressors. Future strategies should scale mental health services and target specific risk factors.

Maternal depression has become a critical global public health issue, with the WHO emphasizing its priority in the Global Action Plan for Mental Health 2013–2030 and promoting interventions like the Thinking Healthy Manual to address it (Nisar et al., 2020b). To effectively tackle this challenge, the government should incorporate the prevention and treatment of depressive disorders in WCBA into the public health policy framework and develop targeted interventions. In high-SDI regions, more refined mental health services should be provided, with a particular focus on the impact of social and environmental stressors on WCBA (Fan et al., 2025b). For example, a dedicated mental health service platform for WCBA could be established to offer convenient psychological support. Regarding low-SDI regions, priority should be given to strengthening the development of basic mental health services. For example, the coverage of mental health service points should be increased, especially in remote areas. In addition, anti-stigma campaigns should be promoted to address workplace inequalities, and digital health monitoring systems and regional registration systems should be implemented to enhance surveillance (Howard et al., 2025). At the same time, treatment subsidy initiatives should be introduced for WCBA in low-income regions, while integrating routine mental health screening into prenatal and postnatal care. To effectively manage depression among WCBA, it is essential to implement stratified interventions based on age groups. For adolescents, mental health education and early screening should be conducted to disseminate knowledge and provide psychological counseling services. For maternal women, enhanced psychological interventions should be provided, focusing on depression during maternal period, along with emotional support (Żyrek et al., 2024). For women in perimenopause, hormone replacement therapy and psychological support should be offered to help them transition smoothly.

This research possesses the subsequent limitations: initially, employing GBD 2021 to assess the global burden of disease for depressive disorders among WCBA may reveal discrepancies in data quality attributable to varying data collection methodologies across countries, particularly in remote and impoverished regions where data gathering poses significant challenges, thereby indirectly influencing the reliability of the data. Secondly, the use of different diagnostic criteria (ICD-10 and DSM-IV-TR) and the transition to DSM-5 could introduce further inconsistencies, especially in settings where diagnostic standards are not uniformly applied. Moreover, in low-resource settings, underreporting and misdiagnosis are likely due to limited access to mental health services and inadequate diagnostic infrastructure. Future work should focus on harmonizing diagnostic criteria and improving data collection in low-resource settings using digital health technologies and community-based surveys. Third, the GBD may have neglected potential influences, including genetic and environmental factors, in forecasting disease burden, thereby compromising the accuracy of the findings. Fourth, population groups across regions and nations may differ in age structure, which could affect the comparability of depressive disorder burden estimates. While ASRs were calculated to mitigate this by aligning with a standard age distribution, residual impacts of age structure disparities may still exist. Fifth, the Nordpred model relies on the assumption that pre-existing epidemiological trends continue unchanged. This assumption may be challenged by unexpected events such as the COVID-19 pandemic, which has disrupted established trends in some health outcomes, and by unforeseen future factors including policy changes (e.g., new mental health intervention policies), both of which could potentially affect the predictive validity of Nordpred model. Ultimately, while we analyzed the incidence, prevalence, and DALYs of depressive disorders among WCBA in the 15–49 age demographic, we did not further delineate its risk factors, which may constrain a thorough comprehension of WCBA depressive disorders.

## 5 Conclusion

This study illustrated the rising trends in ASIR, ASPR, and ASDALYR among WCBA globally from 1990 to 2021, emphasizing the significance of geographical and age-related factors. Governments must urgently implement targeted interventions to alleviate the burden of depressive disorders among WCBA globally.

## Data Availability

Publicly available datasets were analyzed in this study. This data can be found at: the data is sourced from a public database, accessible via the following link: https://vizhub.healthdata.org/gbd-results/.
