# Supplementary material for: Global, regional, and national burden and trends of depressive disorders among women of childbearing age from 1990 to 2021: insights from GBD 2021
Source: Front Psychol. 2025 Aug 13;16:1594430. doi: 10.3389/fpsyg.2025.1594430 (PMC12382448; doi:10.3389/fpsyg.2025.1594430)

**Global, regional, and national burden and trends of depressive disorders among women of childbearing age from 1990 to 2021: insights from GBD 2021**

**Supplementary materials**

| Items | Page | Content |
| --- | --- | --- |
| Table S1 | 2 | The incidence, prevalence, and DALYs of depressive disorders cases and rates among WCBA in 1990. |
| Table S2 | 4 | The percentage changes of depressive disorders cases among WCBA in global and regions from 1990 to 2021. |
| Table S3 | 7 | The percentage changes of depressive disorders cases among WCBA in 204 countries and territories from 1990 to 2021. |
| Table S4 | 16 | The incidence of depressive disorders cases and rates among WCBA in 204 countries and territories in 1990 and 2021, and the trends from 1990 to 2021. |
| Table S5 | 34 | The prevalence of depressive disorders cases and rates among WCBA in 204 countries and territories in 1990 and 2021, and the trends from 1990 to 2021. |
| Table S6 | 52 | The DALYs of depressive disorders cases and rates among WCBA in 204 countries and territories in 1990 and 2021, and the trends from 1990 to 2021. |
| Figure S1 | 68 | The numbers and rates of incidence (A), prevalence (B), and DALYs (C) for depressive disorders among WCBA by SDI regions in 1990 and 2021. |
| Figure S2 | 69 | The numbers and rates of incidence, prevalence, and DALYs for depressive disorders among WCBA by 21 regions in 1990 and 2021 |
| Figure S3 | 70 | The numbers and rates of incidence, prevalence, and DALYs for depressive disorders among WCBA in different age-groups in 1990 and 2021. |
| Figure S4 | 71 | The trends of incidence, prevalence, and DALYs for depressive disorders among WCBA in different age-groups from 1990 to 2021. |

**Supplementary Table S1** The incidence, prevalence, and DALYs of depressive disorders cases and rates among WCBA in 1990.

| Location | Incidence Cases×10^5^  (95% UI) | ASIR, per 100,000 (95% UI) | Prevalence Cases×10^5^  (95% UI) | ASPR, per 100,000 (95% UI) | DALYs×10^5^ (95%UI) | ASDALYR, per 100,000 (95% UI) |
| --- | --- | --- | --- | --- | --- | --- |
| Global | 777.23 (588.6 to 1025.39) | 5898.5 (4486.52 to 7740.66) | 723.46 (577.92 to 899.33) | 5545.28 (4447.47 to 6858.89) | 124.45 (80.84 to 180.23) | 948.86 (617.06 to 1369.93) |
| High-middle SDI | 149.67 (114.69 to 193.95) | 5424.7 (4166.97 to 7008.76) | 143.09 (115.27 to 175.41) | 5218.03 (4212.45 to 6381.87) | 24.49 (16.01 to 35.18) | 889.89 (582.12 to 1275.93) |
| High SDI | 140.96 (111.72 to 178.14) | 6219.44 (4915.78 to 7869.11) | 132.33 (109.17 to 159.5) | 5796.66 (4770.5 to 7004.28) | 22.92 (15.26 to 32.52) | 1007.25 (670.08 to 1431.55) |
| Low-middle SDI | 183.92 (134.57 to 250.13) | 7035.36 (5182.88 to 9485.43) | 162.17 (126.28 to 205.22) | 6257.67 (4900.48 to 7860.3) | 28.32 (17.99 to 41.76) | 1088.11 (691.58 to 1595.85) |
| Low SDI | 75.54 (53.54 to 104.72) | 7097.66 (5079.82 to 9744.65) | 69.47 (53.56 to 89.41) | 6575.38 (5096.98 to 8389.51) | 11.84 (7.47 to 17.52) | 1116.33 (706.66 to 1643.03) |
| Middle SDI | 226.49 (169.98 to 299.22) | 5183.69 (3915.67 to 6798.65) | 215.77 (171.81 to 267.83) | 5051.06 (4048.65 to 6224.12) | 36.77 (23.85 to 53.45) | 850.96 (552.69 to 1231.79) |
| Andean Latin America | 4.39 (3.04 to 6.17) | 4737 (3296.37 to 6625.44) | 3.95 (2.96 to 5.23) | 4327.74 (3255.28 to 5700.01) | 0.69 (0.42 to 1.05) | 751.79 (460.41 to 1140.63) |
| Australasia | 4.8 (3.69 to 6.16) | 8977.64 (6898.68 to 11536.06) | 4.08 (3.25 to 5.03) | 7599.59 (6055.54 to 9386.46) | 0.74 (0.48 to 1.07) | 1380.17 (896.99 to 1991.52) |
| Caribbean | 7.08 (5.08 to 9.7) | 7697.77 (5552.01 to 10488.7) | 5.77 (4.35 to 7.52) | 6320.15 (4798.37 to 8195.97) | 1.06 (0.67 to 1.61) | 1161.78 (729.68 to 1756.61) |
| Central Asia | 7.63 (5.44 to 10.49) | 4676.28 (3367.02 to 6381.95) | 7.45 (5.71 to 9.64) | 4607.34 (3546.52 to 5930.09) | 1.26 (0.79 to 1.85) | 772.01 (488.55 to 1132.2) |
| Central Europe | 12.73 (9.43 to 16.98) | 4115.36 (3043.59 to 5505.79) | 13.2 (10.39 to 16.59) | 4241.09 (3332.5 to 5343.85) | 2.16 (1.37 to 3.13) | 695.87 (442.07 to 1011.41) |
| Central Latin America | 20.56 (14.74 to 28.31) | 5119.69 (3689.12 to 6999.46) | 17.75 (13.66 to 23.01) | 4465.04 (3447.45 to 5739.42) | 3.16 (2 to 4.72) | 791.2 (499.51 to 1175.67) |
| Central Sub-Saharan Africa | 12.77 (8.8 to 18.21) | 10640.71 (7408.07 to 15062.94) | 10.62 (7.81 to 14.23) | 8977.33 (6650.3 to 11919.06) | 1.9 (1.17 to 2.91) | 1597.25 (989.08 to 2430.71) |
| East Asia | 145.74 (110.64 to 190.51) | 4412.52 (3367.36 to 5732.14) | 150.22 (121.22 to 184.01) | 4689.83 (3804.73 to 5717.47) | 24.91 (16.24 to 36.12) | 766.37 (500.81 to 1106.86) |
| Eastern Europe | 30.23 (21.88 to 40.91) | 5392.25 (3903.27 to 7297.88) | 28.57 (22.52 to 36.06) | 5063.54 (3985.84 to 6407.4) | 4.86 (3.09 to 7.13) | 864.27 (549.26 to 1266.81) |
| Eastern Sub-Saharan Africa | 29.93 (21.33 to 41.33) | 7375.7 (5319.95 to 10067.85) | 28.62 (22.15 to 36.71) | 7087.75 (5520.1 to 9011.64) | 4.81 (3.03 to 7.08) | 1188.39 (751.52 to 1736.98) |
| High-income Asia Pacific | 17.42 (13.55 to 22.41) | 3838.31 (2978.91 to 4945.44) | 15.97 (13.08 to 19.39) | 3508.02 (2868.29 to 4262.88) | 2.81 (1.84 to 4.02) | 618.37 (403.62 to 885.93) |
| High-income North America | 52.75 (41.02 to 67.58) | 7145.82 (5538.1 to 9178.7) | 52.39 (42.77 to 63.52) | 7007.87 (5700.86 to 8531.26) | 8.87 (5.87 to 12.72) | 1193.29 (788.61 to 1716.28) |
| North Africa and Middle East | 65.86 (47.04 to 91.17) | 8629.46 (6216.24 to 11853.62) | 55.64 (42.31 to 72.88) | 7378.92 (5652.37 to 9576.45) | 10.06 (6.27 to 15.02) | 1323.9 (828.06 to 1967.04) |
| Oceania | 0.62 (0.43 to 0.88) | 3968.59 (2766.94 to 5579.09) | 0.66 (0.49 to 0.87) | 4395.87 (3303.6 to 5788.5) | 0.11 (0.07 to 0.16) | 698.73 (437.04 to 1054.93) |
| South Asia | 173.47 (128.98 to 231.85) | 7116.76 (5321.38 to 9440.27) | 151.68 (119.54 to 189.84) | 6252.32 (4948 to 7775.32) | 26.46 (16.92 to 38.95) | 1088.15 (696.41 to 1593.93) |
| Southeast Asia | 38.02 (27.82 to 51.2) | 3196.08 (2353.81 to 4271.57) | 44.37 (34.77 to 56.19) | 3875.31 (3052.48 to 4886.27) | 6.92 (4.46 to 10.13) | 593.89 (383.57 to 865.93) |
| Southern Latin America | 8.69 (6.56 to 11.49) | 6983.96 (5277.99 to 9228.68) | 7.11 (5.55 to 9.09) | 5733.18 (4480.59 to 7315.73) | 1.32 (0.86 to 1.95) | 1063.1 (688.88 to 1563.55) |
| Southern Sub-Saharan Africa | 8.52 (6.39 to 11.26) | 6761.59 (5111.67 to 8866.45) | 7.98 (6.36 to 9.89) | 6388.08 (5117.99 to 7859.14) | 1.36 (0.88 to 1.97) | 1080.34 (703.43 to 1562.11) |
| Tropical Latin America | 32.44 (24.44 to 42.71) | 8304.3 (6295.63 to 10868.09) | 25.89 (20.4 to 32.61) | 6672.9 (5284.8 to 8352.26) | 4.77 (3.09 to 6.97) | 1225.46 (794.24 to 1783.06) |
| Western Europe | 77.77 (61.88 to 97.86) | 8121.24 (6445.97 to 10229.83) | 66.83 (55.19 to 80.62) | 6951.88 (5732.39 to 8402.04) | 12.08 (8.03 to 17.07) | 1258.72 (836.26 to 1781.26) |
| Western Sub-Saharan Africa | 25.84 (18.4 to 35.78) | 6321.17 (4550.59 to 8648.31) | 24.71 (19.08 to 31.78) | 6101.55 (4749.55 to 7767.06) | 4.14 (2.59 to 6.12) | 1017.39 (639.5 to 1494.49) |

**Supplementary Table S2** The percentage changes of depressive disorders cases among WCBA in global and regions from 1990 to 2021.

| Location | percentage change | | |
| --- | --- | --- | --- |
|  | incidence | prevalence | DALYs |
| Global | 71% | 68% | 69% |
| High-middle SDI | 19% | 21% | 20% |
| High SDI | 54% | 40% | 46% |
| Low-middle SDI | 105% | 104% | 105% |
| Low SDI | 162% | 158% | 162% |
| Middle SDI | 59% | 59% | 58% |
| Andean Latin America | 152% | 138% | 145% |
| Australasia | 51% | 48% | 50% |
| Caribbean | 37% | 36% | 36% |
| Central Asia | 79% | 72% | 76% |
| Central Europe | 0% | -4% | -2% |
| Central Latin America | 162% | 143% | 153% |
| Central Sub-Saharan Africa | 181% | 178% | 182% |
| East Asia | -29% | -10% | -19% |
| Eastern Europe | 16% | 9% | 12% |
| Eastern Sub-Saharan Africa | 174% | 165% | 171% |
| High-income Asia Pacific | 5% | 0% | 3% |
| High-income North America | 99% | 66% | 80% |
| North Africa and Middle East | 143% | 139% | 141% |
| Oceania | 131% | 134% | 133% |
| South Asia | 105% | 106% | 106% |
| Southeast Asia | 85% | 79% | 82% |
| Southern Latin America | 61% | 59% | 60% |
| Southern Sub-Saharan Africa | 122% | 108% | 113% |
| Tropical Latin America | 85% | 83% | 84% |
| Western Europe | 21% | 16% | 18% |
| Western Sub-Saharan Africa | 170% | 173% | 173% |

**Supplementary Table S3** The percentage changes of depressive disorders cases among WCBA in 204 countries and territories from 1990 to 2021.

| location | percentage change |  |  |
| --- | --- | --- | --- |
|  | incidence | prevalence | DALYs |
| Afghanistan | 297% | 285% | 293% |
| Albania | 9% | -2% | 3% |
| Algeria | 123% | 122% | 122% |
| American Samoa | 11% | 10% | 9% |
| Andorra | 66% | 59% | 62% |
| Angola | 270% | 262% | 267% |
| Antigua and Barbuda | 99% | 89% | 93% |
| Argentina | 80% | 75% | 78% |
| Armenia | 25% | 13% | 18% |
| Australia | 52% | 50% | 51% |
| Austria | 0% | 1% | 0% |
| Azerbaijan | 107% | 90% | 98% |
| Bahamas | 104% | 93% | 98% |
| Bahrain | 210% | 208% | 207% |
| Bangladesh | 117% | 114% | 115% |
| Barbados | 46% | 37% | 41% |
| Belarus | 35% | 22% | 28% |
| Belgium | 42% | 32% | 36% |
| Belize | 286% | 268% | 277% |
| Benin | 227% | 217% | 224% |
| Bermuda | -12% | -14% | -13% |
| Bhutan | 47% | 50% | 49% |
| Bolivia (Plurinational State of) | 180% | 167% | 173% |
| Bosnia and Herzegovina | -31% | -32% | -32% |
| Botswana | 207% | 184% | 192% |
| Brazil | 83% | 81% | 82% |
| Brunei Darussalam | 116% | 108% | 112% |
| Bulgaria | -7% | -14% | -11% |
| Burkina Faso | 167% | 165% | 168% |
| Burundi | 140% | 141% | 141% |
| Cabo Verde | 174% | 153% | 164% |
| Cambodia | 82% | 87% | 86% |
| Cameroon | 265% | 255% | 261% |
| Canada | 54% | 42% | 48% |
| Central African Republic | 128% | 126% | 129% |
| Chad | 202% | 197% | 200% |
| Chile | 39% | 39% | 38% |
| China | -30% | -11% | -20% |
| Colombia | 44% | 49% | 46% |
| Comoros | 120% | 111% | 116% |
| Congo | 183% | 181% | 183% |
| Cook Islands | 8% | 7% | 6% |
| Costa Rica | 136% | 124% | 129% |
| Coted'Ivoire | 160% | 157% | 161% |
| Croatia | -24% | -24% | -24% |
| Cuba | -35% | -32% | -34% |
| Cyprus | 117% | 109% | 113% |
| Czechia | -2% | -3% | -2% |
| Democratic People's Republic of Korea | 11% | 18% | 15% |
| Democratic Republic of the Congo | 158% | 157% | 160% |
| Denmark | -15% | -13% | -14% |
| Djibouti | 298% | 284% | 291% |
| Dominica | 31% | 25% | 29% |
| Dominican Republic | 89% | 85% | 87% |
| Ecuador | 153% | 142% | 147% |
| Egypt | 150% | 139% | 145% |
| El Salvador | 43% | 44% | 44% |
| Equatorial Guinea | 294% | 290% | 296% |
| Eritrea | 128% | 124% | 129% |
| Estonia | -28% | -28% | -28% |
| Eswatini | 176% | 143% | 155% |
| Ethiopia | 153% | 145% | 150% |
| Fiji | 42% | 35% | 39% |
| Finland | -5% | -7% | -6% |
| France | 5% | 3% | 4% |
| Gabon | 154% | 149% | 152% |
| Gambia | 191% | 187% | 188% |
| Georgia | -20% | -27% | -24% |
| Germany | 29% | 17% | 23% |
| Ghana | 191% | 184% | 189% |
| Greece | 11% | 7% | 9% |
| Greenland | -4% | -6% | -5% |
| Grenada | 76% | 68% | 72% |
| Guam | 28% | 21% | 24% |
| Guatemala | 213% | 202% | 209% |
| Guinea | 173% | 162% | 169% |
| Guinea-Bissau | 158% | 150% | 154% |
| Guyana | 52% | 45% | 49% |
| Haiti | 172% | 166% | 170% |
| Honduras | 291% | 266% | 278% |
| Hungary | -20% | -18% | -19% |
| Iceland | 19% | 20% | 19% |
| India | 91% | 94% | 94% |
| Indonesia | 111% | 96% | 103% |
| Iran (Islamic Republic of) | 122% | 122% | 122% |
| Iraq | 191% | 188% | 188% |
| Ireland | 87% | 77% | 82% |
| Israel | 108% | 103% | 106% |
| Italy | 9% | 4% | 7% |
| Jamaica | 80% | 71% | 76% |
| Japan | 5% | -1% | 2% |
| Jordan | 300% | 301% | 299% |
| Kazakhstan | 48% | 40% | 44% |
| Kenya | 174% | 172% | 173% |
| Kiribati | 70% | 74% | 72% |
| Kuwait | 265% | 273% | 266% |
| Kyrgyzstan | 110% | 100% | 105% |
| Lao People's Democratic Republic | 97% | 107% | 105% |
| Latvia | -27% | -30% | -29% |
| Lebanon | 229% | 202% | 215% |
| Lesotho | 79% | 68% | 70% |
| Liberia | 168% | 165% | 168% |
| Libya | 163% | 161% | 160% |
| Lithuania | -13% | -19% | -16% |
| Luxembourg | 48% | 50% | 48% |
| Madagascar | 208% | 196% | 203% |
| Malawi | 146% | 137% | 145% |
| Malaysia | 134% | 122% | 128% |
| Maldives | 100% | 128% | 114% |
| Mali | 187% | 183% | 186% |
| Malta | 17% | 13% | 15% |
| Marshall Islands | 65% | 67% | 65% |
| Mauritania | 144% | 138% | 143% |
| Mauritius | 14% | 14% | 13% |
| Mexico | 219% | 186% | 203% |
| Micronesia (Federated States of) | 17% | 19% | 18% |
| Monaco | 31% | 24% | 27% |
| Mongolia | 72% | 76% | 74% |
| Montenegro | 31% | 19% | 24% |
| Morocco | 86% | 82% | 83% |
| Mozambique | 194% | 175% | 182% |
| Myanmar | 105% | 81% | 92% |
| Namibia | 185% | 157% | 171% |
| Nauru | 36% | 30% | 29% |
| Nepal | 196% | 173% | 188% |
| Netherlands | 11% | 7% | 9% |
| New Zealand | 42% | 40% | 43% |
| Nicaragua | 158% | 151% | 155% |
| Niger | 196% | 196% | 198% |
| Nigeria | 144% | 159% | 154% |
| Niue | -10% | -14% | 0% |
| North Macedonia | 47% | 34% | 40% |
| Northern Mariana Islands | 11% | 4% | 5% |
| Norway | 45% | 38% | 42% |
| Oman | 276% | 265% | 270% |
| Pakistan | 191% | 186% | 189% |
| Palau | 6% | 7% | 3% |
| Palestine | 250% | 243% | 246% |
| Panama | 135% | 124% | 129% |
| Papua New Guinea | 168% | 176% | 173% |
| Paraguay | 192% | 175% | 183% |
| Peru | 137% | 123% | 130% |
| Philippines | 125% | 117% | 122% |
| Poland | 21% | 11% | 16% |
| Portugal | 5% | 3% | 4% |
| Puerto Rico | -1% | -6% | -4% |
| Qatar | 629% | 634% | 629% |
| Republic of Korea | 7% | 5% | 6% |
| Republic of Moldova | -10% | -12% | -11% |
| Romania | -1% | -9% | -5% |
| Russian Federation | 25% | 16% | 20% |
| Rwanda | 123% | 123% | 123% |
| Saint Kitts and Nevis | 107% | 101% | 106% |
| Saint Lucia | 97% | 87% | 92% |
| Saint Vincent and the Grenadines | 47% | 41% | 44% |
| Samoa | 39% | 41% | 40% |
| San Marino | 50% | 43% | 46% |
| Sao Tome and Principe | 147% | 141% | 144% |
| Saudi Arabia | 280% | 274% | 276% |
| Senegal | 181% | 164% | 173% |
| Serbia | -2% | -5% | -4% |
| Seychelles | 79% | 70% | 74% |
| Sierra Leone | 148% | 141% | 146% |
| Singapore | -8% | 1% | -4% |
| Slovakia | 27% | 18% | 22% |
| Slovenia | -17% | -16% | -17% |
| Solomon Islands | 142% | 148% | 145% |
| Somalia | 258% | 233% | 248% |
| South Africa | 120% | 107% | 111% |
| South Sudan | 101% | 94% | 98% |
| Spain | 58% | 46% | 51% |
| Sri Lanka | 14% | 20% | 17% |
| Sudan | 166% | 162% | 164% |
| Suriname | 123% | 113% | 118% |
| Sweden | 28% | 24% | 26% |
| Switzerland | 12% | 11% | 11% |
| Syrian Arab Republic | 74% | 69% | 71% |
| Taiwan (Province of China) | 33% | 28% | 30% |
| Tajikistan | 162% | 151% | 156% |
| Thailand | 13% | 16% | 15% |
| Timor-Leste | 85% | 82% | 84% |
| Togo | 177% | 173% | 176% |
| Tokelau | 7% | 0% | 50% |
| Tonga | 22% | 22% | 22% |
| Trinidad and Tobago | 43% | 39% | 41% |
| Tunisia | 109% | 101% | 103% |
| Turkey | 87% | 83% | 85% |
| Turkmenistan | 69% | 64% | 66% |
| Tuvalu | 37% | 28% | 35% |
| Uganda | 203% | 196% | 203% |
| Ukraine | 2% | -2% | 0% |
| United Arab Emirates | 492% | 489% | 487% |
| United Kingdom | 20% | 18% | 18% |
| United Republic of Tanzania | 177% | 169% | 178% |
| United States of America | 103% | 68% | 83% |
| United States Virgin Islands | -23% | -27% | -25% |
| Uruguay | 67% | 57% | 62% |
| Uzbekistan | 109% | 108% | 109% |
| Vanuatu | 135% | 136% | 134% |
| Venezuela (Bolivarian Republic of) | 69% | 66% | 67% |
| Viet Nam | 63% | 70% | 67% |
| Yemen | 204% | 205% | 205% |
| Zambia | 222% | 205% | 214% |
| Zimbabwe | 117% | 101% | 109% |

**Supplementary Table S4** The incidence of depressive disorders cases and rates among WCBA in 204 countries and territories in 1990 and 2021, and the trends from 1990 to 2021.

| Location | Incidence | | | | |
| --- | --- | --- | --- | --- | --- |
|  | No.,1990 (95% UI) | ASIR, 1990 per 100,000 (95% UI) | No.,2021, (95% UI) | ASIR, 2021 per 100,000 (95% UI) | EAPC, 1990-2021, (95% CI) |
| Afghanistan | 205540 (138294 to 296616) | 9789.34 (6680.56 to 14037.36) | 816358 (524875 to 1209087) | 11768.52 (7634.29 to 17317.93) | 0.12 (-0.04 to 0.29) |
| Albania | 29921 (20197 to 42831) | 3628.64 (2460.52 to 5160.27) | 32574 (21079 to 49306) | 5277.31 (3397.48 to 8023.34) | 0.41 (0.15 to 0.67) |
| Algeria | 452078 (293400 to 668457) | 8078.23 (5309.81 to 11859.45) | 1009296 (629591 to 1568687) | 8896.84 (5535.07 to 13853.61) | 0.03 (-0.14 to 0.21) |
| American Samoa | 359 (250 to 505) | 2984.33 (2082.83 to 4181.73) | 398 (258 to 593) | 3386.91 (2191.41 to 5058) | 0.06 (-0.07 to 0.18) |
| Andorra | 1125 (774 to 1608) | 7509.87 (5147.37 to 10737.52) | 1863 (1186 to 2817) | 9421.87 (5898.04 to 14408.85) | 0.17 (-0.11 to 0.44) |
| Angola | 251570 (170661 to 365443) | 11259.98 (7705.44 to 16274.44) | 930141 (593451 to 1400476) | 12452.72 (7995.46 to 18644.24) | -0.02 (-0.15 to 0.11) |
| Antigua and Barbuda | 832 (546 to 1235) | 5203.16 (3422.64 to 7679.03) | 1653 (1028 to 2522) | 6740.22 (4173.29 to 10343.99) | 0.27 (0 to 0.55) |
| Argentina | 439553 (305677 to 621728) | 5470.55 (3809.14 to 7735.45) | 792741 (562167 to 1093993) | 6744.09 (4777.34 to 9312.56) | 0 (-0.26 to 0.25) |
| Armenia | 32519 (21897 to 47016) | 3837.79 (2592.65 to 5513.56) | 40675 (25706 to 61860) | 5478.11 (3438.51 to 8360.33) | 0.43 (0.13 to 0.74) |
| Australia | 412157 (314848 to 528251) | 9273.68 (7075.4 to 11894.28) | 626911 (417154 to 892852) | 10655.35 (7063.86 to 15206.69) | 0.26 (0.06 to 0.45) |
| Austria | 142071 (101785 to 195200) | 7151.91 (5102.17 to 9834.51) | 142777 (94441 to 208072) | 7277.52 (4763.02 to 10693.65) | -0.66 (-0.97 to -0.35) |
| Azerbaijan | 69319 (46460 to 101461) | 3824.06 (2578.15 to 5542.45) | 143452 (90214 to 215745) | 5198.02 (3254.37 to 7833.49) | 0.32 (0.02 to 0.61) |
| Bahamas | 3725 (2446 to 5480) | 5246.74 (3457.88 to 7674.4) | 7604 (4748 to 11837) | 7015 (4370.79 to 10942.42) | 0.33 (0.04 to 0.62) |
| Bahrain | 11525 (7862 to 16352) | 10136.38 (6938.12 to 14275.73) | 35755 (22959 to 53654) | 10918.27 (6995.23 to 16393.05) | -0.44 (-0.64 to -0.23) |
| Bangladesh | 2156228 (1490823 to 3047094) | 9216.86 (6415.51 to 12894.38) | 4678226 (3064226 to 6879649) | 10285.19 (6747.07 to 15097.48) | -0.04 (-0.2 to 0.13) |
| Barbados | 3531 (2326 to 5194) | 5192.45 (3424.9 to 7610.32) | 5170 (3242 to 8000) | 7171.97 (4471.91 to 11152.53) | 0.37 (0.11 to 0.62) |
| Belarus | 156789 (109386 to 215473) | 6148.13 (4292.32 to 8445.64) | 210932 (138836 to 305985) | 9514.3 (6209.17 to 13916) | 0.26 (-0.04 to 0.56) |
| Belgium | 157631 (129973 to 190838) | 6460.3 (5320.32 to 7835.35) | 223080 (148818 to 326209) | 8949.29 (5908.1 to 13159.12) | 0.87 (0.65 to 1.09) |
| Belize | 2146 (1416 to 3207) | 5320.68 (3534.05 to 7855.26) | 8277 (5257 to 12649) | 6906.33 (4397.18 to 10526.6) | 0.4 (0.19 to 0.62) |
| Benin | 67630 (45961 to 99243) | 6513.85 (4473.65 to 9429.81) | 221364 (140688 to 338631) | 7187.46 (4625.16 to 10903.52) | 0.1 (0.02 to 0.18) |
| Bermuda | 1098 (764 to 1564) | 6369.58 (4427.23 to 9078.89) | 968 (597 to 1471) | 7062.48 (4310.56 to 10801.62) | -0.42 (-0.7 to -0.15) |
| Bhutan | 8669 (5920 to 12478) | 6797.43 (4688.33 to 9657.02) | 12702 (8013 to 19278) | 6236.3 (3945.5 to 9425.16) | -0.36 (-0.5 to -0.23) |
| Bolivia (Plurinational State of) | 98633 (67791 to 141852) | 6681.26 (4629.36 to 9516.46) | 276564 (178571 to 414435) | 8903.55 (5755.43 to 13307.36) | 0.03 (-0.37 to 0.42) |
| Bosnia and Herzegovina | 63751 (44599 to 89546) | 5496.67 (3850.86 to 7710.13) | 44036 (27249 to 66151) | 5889.63 (3630.26 to 8890.22) | -0.88 (-1.21 to -0.56) |
| Botswana | 19259 (13100 to 27760) | 6407.73 (4409.33 to 9157.88) | 59036 (38133 to 88930) | 8683.77 (5618.04 to 13054.18) | 0.18 (-0.02 to 0.37) |
| Brazil | 3184585 (2401981 to 4188220) | 8350.53 (6337.44 to 10915.98) | 5832146 (4365409 to 7625893) | 9772.37 (7286.12 to 12828.54) | -0.46 (-0.84 to -0.08) |
| Brunei Darussalam | 1939 (1294 to 2838) | 2817.26 (1889.74 to 4092.98) | 4191 (2650 to 6495) | 3383.89 (2135.05 to 5255.84) | 0.19 (-0.04 to 0.43) |
| Bulgaria | 95487 (68627 to 131994) | 4551.81 (3257.62 to 6321.82) | 89168 (58106 to 132137) | 6120.75 (3942.92 to 9174.25) | -0.4 (-0.78 to -0.02) |
| Burkina Faso | 124804 (85304 to 178432) | 6251.96 (4319.15 to 8845.19) | 333696 (214160 to 508386) | 6407.15 (4148.46 to 9659.26) | 0.38 (0.24 to 0.52) |
| Burundi | 100405 (67213 to 143560) | 8439.76 (5696.39 to 11941.38) | 240532 (150943 to 369178) | 8064.43 (5114.14 to 12279.66) | -0.79 (-0.99 to -0.58) |
| Cabo Verde | 4619 (3083 to 6774) | 6286.69 (4261.78 to 9110.39) | 12676 (8007 to 19436) | 8533.65 (5407.55 to 13037.59) | 0.45 (0.26 to 0.64) |
| Cambodia | 110682 (76167 to 158136) | 4413.87 (3060.6 to 6257.73) | 201738 (132139 to 297360) | 4485.28 (2941.86 to 6604.57) | -0.85 (-1.07 to -0.63) |
| Cameroon | 154105 (104175 to 224417) | 6859.32 (4690 to 9873.78) | 561766 (355092 to 856716) | 7532.16 (4803.66 to 11355.52) | 0.07 (0 to 0.14) |
| Canada | 475939 (363686 to 628402) | 6505.12 (4953.6 to 8609.49) | 734711 (493946 to 1067868) | 9096.42 (6066.77 to 13261.55) | 0.09 (-0.23 to 0.41) |
| Central African Republic | 69771 (47157 to 101553) | 11184.32 (7597.35 to 16123.72) | 159402 (102006 to 242847) | 11824.85 (7626.16 to 17910.14) | 0.03 (-0.08 to 0.15) |
| Chad | 102281 (68768 to 150314) | 8105.08 (5520.02 to 11813.21) | 309154 (194460 to 465500) | 8575.24 (5462.63 to 12744.3) | 0.11 (0.02 to 0.2) |
| Chile | 384726 (303942 to 485080) | 10548.91 (8335.82 to 13280.88) | 534248 (347236 to 788743) | 11524.79 (7463.88 to 17023.93) | -0.41 (-0.68 to -0.13) |
| China | 14240888 (10817208 to 18598630) | 4461.64 (3407.01 to 5789.15) | 9996508 (7608235 to 12842414) | 2947.48 (2221.51 to 3825.84) | -1.45 (-1.7 to -1.19) |
| Colombia | 311171 (210579 to 454628) | 3661.16 (2483.83 to 5326.32) | 447994 (290102 to 666689) | 3409.81 (2207.17 to 5071.72) | -1.07 (-1.48 to -0.65) |
| Comoros | 6013 (4066 to 8701) | 6036.27 (4136.44 to 8642.49) | 13247 (8345 to 20152) | 6884.57 (4351.6 to 10431.66) | -0.06 (-0.23 to 0.11) |
| Congo | 60330 (40740 to 87212) | 11166.76 (7617.65 to 16003.27) | 170622 (108778 to 260960) | 12064.56 (7724.91 to 18394.7) | -0.14 (-0.33 to 0.06) |
| Cook Islands | 184 (118 to 283) | 4014.99 (2590.55 to 6145.59) | 199 (121 to 317) | 4628.32 (2799.75 to 7367.31) | 0.12 (0.03 to 0.21) |
| Costa Rica | 40204 (26648 to 58797) | 5320.67 (3544.46 to 7734.65) | 94764 (60052 to 143631) | 7234.43 (4580.38 to 10979.93) | 0.48 (0.29 to 0.68) |
| Coted'Ivoire | 145557 (98988 to 209664) | 5652.57 (3902.16 to 8024.77) | 377793 (243107 to 567906) | 5891.69 (3820.1 to 8793.18) | -0.01 (-0.1 to 0.09) |
| Croatia | 65665 (46873 to 89125) | 5395.2 (3842.86 to 7337.58) | 50177 (33421 to 73903) | 5421.75 (3568.93 to 8063.19) | -0.83 (-1.13 to -0.53) |
| Cuba | 315640 (228163 to 425603) | 10320.08 (7465.85 to 13883.76) | 205902 (134507 to 306460) | 8038.92 (5199.22 to 12075.83) | -1.86 (-2.24 to -1.48) |
| Cyprus | 12210 (8012 to 17923) | 6183.27 (4051.53 to 9080) | 26508 (16461 to 40936) | 7549.47 (4646.92 to 11685.18) | 0.19 (-0.04 to 0.43) |
| Czechia | 138723 (99197 to 191034) | 5290.94 (3769.49 to 7317.59) | 136620 (88137 to 200104) | 5708.58 (3643.19 to 8498.6) | -0.68 (-0.97 to -0.39) |
| Democratic People's Republic of Korea | 197026 (140890 to 273427) | 3501.89 (2507.49 to 4848.56) | 218816 (145731 to 317136) | 3255.02 (2157.79 to 4737.78) | -0.52 (-0.62 to -0.43) |
| Democratic Republic of the Congo | 862486 (589967 to 1237664) | 10403.1 (7184.81 to 14830.23) | 2226408 (1422647 to 3297302) | 10731.63 (6910.47 to 15791.67) | -0.03 (-0.14 to 0.08) |
| Denmark | 127726 (92483 to 173817) | 9606.6 (6916.83 to 13124.53) | 108627 (70609 to 160314) | 8551.74 (5513.98 to 12668.25) | -1.04 (-1.37 to -0.72) |
| Djibouti | 5916 (3917 to 8740) | 6352.84 (4257.51 to 9306.99) | 23557 (15054 to 35368) | 7351.61 (4708.6 to 11019.99) | 0.14 (0 to 0.28) |
| Dominica | 861 (561 to 1285) | 5219.59 (3420.48 to 7749) | 1132 (702 to 1744) | 6889.17 (4269.82 to 10616.38) | 0.28 (0.02 to 0.55) |
| Dominican Republic | 125951 (83240 to 186321) | 6930.5 (4621.28 to 10139.31) | 238393 (147615 to 368409) | 8246.8 (5106.45 to 12731.05) | 0.17 (-0.04 to 0.37) |
| Ecuador | 144466 (98416 to 205341) | 5771.56 (3941.76 to 8155.76) | 366019 (236085 to 546924) | 7747.17 (4998.46 to 11570.55) | 0.27 (-0.04 to 0.59) |
| Egypt | 924352 (605183 to 1370868) | 7183.65 (4729.47 to 10597.53) | 2310619 (1436897 to 3527716) | 8949.98 (5581.89 to 13627.29) | 0.27 (0.05 to 0.49) |
| El Salvador | 100395 (69545 to 142841) | 7718.14 (5361.58 to 10947.37) | 143679 (93314 to 213141) | 8065.43 (5232.67 to 11962.43) | -0.42 (-0.66 to -0.18) |
| Equatorial Guinea | 10907 (7405 to 15816) | 11358.42 (7768.2 to 16336.75) | 43025 (27513 to 65455) | 12062.3 (7744.19 to 18264.46) | 0.03 (-0.09 to 0.16) |
| Eritrea | 54263 (36899 to 77495) | 7242.49 (4979.64 to 10269.15) | 123943 (79133 to 189561) | 7719.67 (4963.5 to 11714.45) | -0.09 (-0.2 to 0.02) |
| Estonia | 31091 (22167 to 42609) | 8014.39 (5701.91 to 11007.15) | 22408 (14535 to 33210) | 7753.19 (4983.9 to 11625.24) | -1.33 (-1.64 to -1.02) |
| Eswatini | 11556 (7835 to 16399) | 6330.13 (4348.34 to 8892.48) | 31937 (20459 to 47630) | 10352.94 (6655.63 to 15376.45) | 0.67 (0.4 to 0.94) |
| Ethiopia | 737811 (527007 to 1014343) | 6923.76 (4968.39 to 9447.47) | 1863646 (1310365 to 2611122) | 7061.36 (4998.41 to 9814.81) | -0.4 (-0.59 to -0.22) |
| Fiji | 6945 (4920 to 9832) | 3548.9 (2521.57 to 5002.91) | 9892 (6547 to 14588) | 4334.15 (2868.97 to 6392.57) | 0.02 (-0.14 to 0.18) |
| Finland | 132135 (95359 to 177150) | 10400.92 (7451.35 to 13989.86) | 125247 (83659 to 179521) | 11190.88 (7432.99 to 16114.85) | -0.54 (-0.79 to -0.28) |
| France | 1379767 (1141135 to 1650359) | 9517.04 (7864.16 to 11387.59) | 1453396 (968319 to 2113067) | 10135.1 (6704.6 to 14817.77) | -0.35 (-0.58 to -0.11) |
| Gabon | 21560 (14575 to 31419) | 10100.05 (6913.27 to 14598.17) | 54697 (35035 to 81048) | 11365.03 (7322.95 to 16744.55) | -0.08 (-0.23 to 0.07) |
| Gambia | 24440 (16498 to 35488) | 11604.87 (7945.32 to 16567.55) | 71058 (44791 to 107636) | 12222.79 (7783.47 to 18335.83) | -0.49 (-0.68 to -0.3) |
| Georgia | 59068 (39884 to 85483) | 4325.25 (2920.33 to 6246.26) | 47299 (29657 to 71306) | 5874.04 (3647.32 to 8910.94) | 0.24 (-0.02 to 0.5) |
| Germany | 1104428 (863021 to 1421011) | 5674.77 (4418.2 to 7317.31) | 1422779 (930261 to 2100302) | 8403.43 (5429.43 to 12482.42) | 1.2 (1 to 1.39) |
| Ghana | 218565 (145176 to 315672) | 6510.17 (4359.81 to 9314.59) | 636423 (395009 to 975494) | 7137.41 (4447.38 to 10872.54) | -0.03 (-0.15 to 0.09) |
| Greece | 268542 (176079 to 394870) | 10634.79 (6957.54 to 15659.78) | 298086 (185147 to 455198) | 13841.31 (8548.39 to 21193.92) | 0 (-0.4 to 0.4) |
| Greenland | 2666 (1933 to 3669) | 17571.9 (12718.38 to 24179.26) | 2564 (1735 to 3642) | 20221.09 (13654.57 to 28734.58) | -0.34 (-0.58 to -0.11) |
| Grenada | 1005 (656 to 1503) | 5330.07 (3503.38 to 7898.55) | 1764 (1102 to 2644) | 6865.95 (4278.57 to 10291.78) | 0.26 (0.05 to 0.46) |
| Guam | 1281 (900 to 1770) | 3661.74 (2585.44 to 5047.96) | 1638 (1091 to 2415) | 4543.96 (3017.32 to 6716.16) | 0.22 (0.01 to 0.42) |
| Guatemala | 118097 (79382 to 174702) | 6870.7 (4672.41 to 10034.64) | 369085 (233728 to 568482) | 8574.38 (5450.88 to 13128.48) | 0.04 (-0.22 to 0.29) |
| Guinea | 88185 (60189 to 127721) | 6690.14 (4604.89 to 9637.47) | 241094 (153609 to 368764) | 7618.44 (4912.78 to 11554.82) | 0.13 (0.02 to 0.25) |
| Guinea-Bissau | 15228 (10339 to 22056) | 6929.13 (4759.79 to 9953.43) | 39289 (25023 to 59575) | 7824.75 (5028.24 to 11786.72) | 0.17 (0.06 to 0.27) |
| Guyana | 17558 (12081 to 24516) | 8716.48 (6036.83 to 12097.62) | 26717 (17297 to 39264) | 13100.09 (8472.65 to 19238.32) | 0.64 (0.43 to 0.84) |
| Haiti | 104959 (70512 to 154756) | 6954.94 (4699.64 to 10190.86) | 285993 (181237 to 428596) | 8115.46 (5156.15 to 12135.23) | -0.02 (-0.2 to 0.16) |
| Honduras | 51598 (33854 to 75567) | 5198.19 (3443.86 to 7546.01) | 201614 (125603 to 308391) | 7256.81 (4544.9 to 11043.44) | 0.4 (0.14 to 0.66) |
| Hungary | 144737 (104666 to 196509) | 5556.22 (4005.17 to 7576.77) | 115533 (76589 to 165910) | 5083.24 (3306.8 to 7396.93) | -1.18 (-1.46 to -0.9) |
| Iceland | 4253 (2995 to 5892) | 6547.03 (4613.74 to 9058.2) | 5056 (3238 to 7591) | 6380.2 (4063.05 to 9601.82) | -0.49 (-0.68 to -0.29) |
| India | 13368733 (9999203 to 17730651) | 6893.72 (5182.5 to 9090.56) | 25586191 (19118508 to 33838603) | 6820.95 (5105.71 to 9002.1) | -1.31 (-1.7 to -0.92) |
| Indonesia | 1304095 (939848 to 1766427) | 2760.81 (2002.69 to 3712.98) | 2752422 (1968416 to 3768385) | 3651.76 (2605.14 to 5012.05) | 0.22 (-0.04 to 0.49) |
| Iran (Islamic Republic of) | 1293093 (903172 to 1824561) | 10405.37 (7334.13 to 14572.62) | 2868731 (2040860 to 3952850) | 12267.94 (8681.08 to 16991.17) | 0.59 (0.38 to 0.8) |
| Iraq | 305181 (208735 to 427977) | 7812.54 (5417.05 to 10859.07) | 888935 (569050 to 1314467) | 8548.05 (5492.56 to 12589.97) | 0.2 (-0.32 to 0.72) |
| Ireland | 70918 (52018 to 94585) | 8048.58 (5916.86 to 10727.34) | 132689 (87244 to 192659) | 11604.98 (7585.28 to 16917.48) | 0.48 (0.23 to 0.74) |
| Israel | 105998 (72519 to 151296) | 8700.12 (5974.04 to 12387.57) | 220954 (138677 to 333526) | 9945.83 (6227.13 to 15043.32) | -0.37 (-0.6 to -0.13) |
| Italy | 1098547 (786342 to 1500642) | 7670.64 (5484.23 to 10487.13) | 1193615 (849122 to 1626463) | 9911.36 (6986.7 to 13625.15) | -0.28 (-0.65 to 0.08) |
| Jamaica | 30345 (19726 to 45386) | 5283.87 (3458.7 to 7839.95) | 54657 (33382 to 85626) | 7060.54 (4306.67 to 11057.66) | 0.27 (-0.01 to 0.55) |
| Japan | 1206511 (936723 to 1551970) | 3813.61 (2946.08 to 4923.21) | 1264770 (973398 to 1631918) | 5301.71 (4040.35 to 6907.97) | 0.64 (0.36 to 0.92) |
| Jordan | 73567 (48643 to 108706) | 9330.6 (6291.26 to 13609.56) | 294119 (184943 to 448371) | 9579.58 (6047 to 14577.74) | -0.4 (-0.57 to -0.23) |
| Kazakhstan | 198449 (141004 to 274990) | 4914.52 (3505.51 to 6784.91) | 293860 (194678 to 425915) | 6190.95 (4086.88 to 9019.6) | 0.48 (0.29 to 0.66) |
| Kenya | 346463 (255242 to 463857) | 7277.69 (5431.17 to 9613.68) | 947907 (695877 to 1273412) | 7469.66 (5523.54 to 9951.2) | -0.35 (-0.52 to -0.17) |
| Kiribati | 701 (495 to 984) | 3709.89 (2633.98 to 5178.69) | 1194 (785 to 1778) | 3727.57 (2460.32 to 5539.51) | -0.33 (-0.46 to -0.2) |
| Kuwait | 32930 (21738 to 48621) | 8037.4 (5333.48 to 11776.6) | 120171 (74947 to 183864) | 7980.21 (4940.59 to 12300.8) | -0.16 (-0.28 to -0.05) |
| Kyrgyzstan | 51999 (36088 to 71923) | 5176.8 (3625.47 to 7112.21) | 109173 (71744 to 160613) | 6360.5 (4180.36 to 9348.04) | 0.04 (-0.2 to 0.27) |
| Lao People's Democratic Republic | 33219 (22867 to 47588) | 3406.51 (2362.09 to 4849.49) | 65575 (42866 to 98171) | 3293.3 (2158.67 to 4914.31) | -0.63 (-0.94 to -0.32) |
| Latvia | 45194 (32148 to 61605) | 6769.86 (4788.94 to 9267.69) | 32992 (21153 to 49277) | 8041.18 (5092.84 to 12129.28) | -0.46 (-0.74 to -0.18) |
| Lebanon | 59091 (41514 to 83416) | 7984.27 (5623.06 to 11231.11) | 194118 (121907 to 295349) | 12984.07 (8110.04 to 19808.95) | 0.49 (0.22 to 0.76) |
| Lesotho | 38287 (26220 to 54997) | 10431.48 (7174.22 to 14915.14) | 68354 (44361 to 100849) | 13845.53 (9036.27 to 20293.29) | 0.42 (0.16 to 0.68) |
| Liberia | 45202 (30515 to 65363) | 8638.94 (5917.77 to 12363.72) | 121344 (77082 to 184161) | 9029.02 (5790.19 to 13612.88) | 0.48 (0.21 to 0.75) |
| Libya | 72684 (47495 to 107271) | 8321.52 (5514.64 to 12123.78) | 191420 (121440 to 286645) | 9530.68 (6032.16 to 14295.7) | 0.12 (-0.03 to 0.28) |
| Lithuania | 65503 (46569 to 89938) | 7019.96 (4977.25 to 9660.25) | 56861 (37913 to 82602) | 9518.64 (6285.07 to 13931.49) | -0.24 (-0.55 to 0.08) |
| Luxembourg | 7842 (5520 to 10681) | 8021 (5623.06 to 10956.24) | 11586 (7981 to 16328) | 7499.55 (5088.38 to 10686.36) | -0.86 (-1.13 to -0.58) |
| Madagascar | 177312 (120243 to 256189) | 6979.22 (4796.06 to 9984.12) | 545313 (341016 to 826310) | 7814.61 (4930.72 to 11745.92) | -0.05 (-0.21 to 0.11) |
| Malawi | 131683 (89275 to 188197) | 6179.38 (4247.09 to 8743.22) | 323814 (206350 to 496852) | 6750.94 (4362.82 to 10269.48) | -0.35 (-0.53 to -0.17) |
| Malaysia | 170182 (116969 to 242787) | 3868.68 (2687.28 to 5472.37) | 397970 (252389 to 601935) | 4749.72 (3011.71 to 7175.43) | 0.98 (0.72 to 1.25) |
| Maldives | 2791 (1950 to 3928) | 5867.65 (4135.11 to 8166.81) | 5591 (3680 to 8164) | 4893.22 (3202.39 to 7172.02) | -1.34 (-1.57 to -1.11) |
| Mali | 98492 (67250 to 141441) | 5366.71 (3699.57 to 7640.57) | 283032 (182276 to 435968) | 5498.16 (3586.76 to 8394.82) | -0.13 (-0.28 to 0.02) |
| Malta | 5856 (3842 to 8504) | 6190.68 (4044.57 to 9018.07) | 6875 (4269 to 10530) | 7393 (4558.34 to 11357.36) | 0.22 (-0.03 to 0.46) |
| Marshall Islands | 343 (238 to 485) | 3483.05 (2442.79 to 4889.65) | 565 (366 to 838) | 3802.65 (2472.15 to 5630.04) | -0.12 (-0.22 to -0.02) |
| Mauritania | 22000 (15026 to 31438) | 4938.37 (3414.09 to 6975.67) | 53751 (34100 to 82209) | 5227.11 (3351.12 to 7927.7) | -0.11 (-0.27 to 0.04) |
| Mauritius | 22296 (15603 to 30747) | 7459.72 (5226.81 to 10244.85) | 25346 (16425 to 37521) | 8044.69 (5198.57 to 11932.65) | -0.55 (-0.79 to -0.31) |
| Mexico | 1101545 (801866 to 1481723) | 5309.93 (3887.06 to 7090.62) | 3509492 (2596250 to 4667560) | 9961.29 (7358.15 to 13268.44) | 1.86 (1.56 to 2.17) |
| Micronesia (Federated States of) | 841 (586 to 1198) | 3611.13 (2537.51 to 5100.37) | 987 (644 to 1457) | 3756.49 (2456.97 to 5529.23) | -0.21 (-0.32 to -0.1) |
| Monaco | 589 (377 to 895) | 8343.53 (5297.83 to 12744.18) | 773 (471 to 1213) | 10882.45 (6591.3 to 17163.88) | 0.26 (0.06 to 0.45) |
| Mongolia | 32723 (22771 to 46487) | 6603.4 (4636.14 to 9251.83) | 56124 (37535 to 82381) | 6569.04 (4380.85 to 9668.5) | -0.29 (-0.35 to -0.23) |
| Montenegro | 6934 (4861 to 9555) | 4457.71 (3129.26 to 6135.65) | 9051 (5952 to 13373) | 6178.86 (4032.62 to 9178.51) | 0.12 (-0.16 to 0.4) |
| Morocco | 610013 (414836 to 876884) | 9975.05 (6841.02 to 14254.01) | 1136466 (724193 to 1689051) | 11687.82 (7437.82 to 17385.89) | 0.02 (-0.2 to 0.25) |
| Mozambique | 205804 (138818 to 299481) | 6790.43 (4628.44 to 9801.32) | 605866 (381523 to 936611) | 8365.67 (5330.74 to 12793.46) | 0.19 (0.05 to 0.33) |
| Myanmar | 200268 (134100 to 287902) | 1950.68 (1317.23 to 2785.01) | 411430 (258645 to 633353) | 2717.48 (1709.34 to 4182.68) | 0.27 (-0.15 to 0.68) |
| Namibia | 16734 (11563 to 23953) | 5240.43 (3653.09 to 7433.1) | 47764 (30419 to 72938) | 7330.27 (4689.58 to 11169.36) | 0 (-0.35 to 0.36) |
| Nauru | 96 (62 to 147) | 3978.8 (2557.41 to 6015.18) | 131 (80 to 210) | 4611.02 (2812.46 to 7356.6) | 0.14 (0.05 to 0.23) |
| Nepal | 308758 (214114 to 432293) | 7201.36 (5030.5 to 10032.1) | 915446 (587598 to 1373095) | 10351.69 (6669.64 to 15459.54) | 0.65 (0.38 to 0.91) |
| Netherlands | 303643 (250303 to 368849) | 7614.49 (6269.91 to 9258.52) | 337063 (218361 to 494679) | 9183.95 (5916.85 to 13526.73) | -0.12 (-0.41 to 0.17) |
| New Zealand | 67845 (47484 to 94157) | 7508.81 (5254.85 to 10418.74) | 96249 (67969 to 135539) | 8307.46 (5853.54 to 11713.79) | 0.14 (0.08 to 0.2) |
| Nicaragua | 52121 (35404 to 75405) | 5945.15 (4051.73 to 8539.81) | 134359 (86504 to 201116) | 7424.67 (4783.63 to 11102.99) | 0.1 (-0.12 to 0.32) |
| Niger | 107832 (73281 to 156754) | 6569.2 (4518.36 to 9432.87) | 319053 (202583 to 480386) | 6471.06 (4176.29 to 9624.15) | -0.06 (-0.12 to -0.01) |
| Nigeria | 1161085 (826176 to 1607067) | 6209.15 (4451.97 to 8515.75) | 2837020 (2031893 to 3878621) | 5211.87 (3755.48 to 7077.52) | -0.85 (-1.05 to -0.66) |
| Niue | 20 (13 to 30) | 3987.89 (2568.47 to 6023.01) | 18 (11 to 28) | 4604.14 (2832.58 to 7206.48) | 0.12 (0.02 to 0.21) |
| North Macedonia | 20746 (14641 to 29180) | 4078.19 (2879.48 to 5734.23) | 30480 (19714 to 45223) | 5686.53 (3650.17 to 8496.58) | -0.03 (-0.41 to 0.36) |
| Northern Mariana Islands | 390 (264 to 572) | 2837.51 (1926.52 to 4122.79) | 432 (282 to 646) | 3773.04 (2445.55 to 5680.18) | 0.32 (0.1 to 0.53) |
| Norway | 74998 (54241 to 101689) | 7096.98 (5126.21 to 9628.44) | 108815 (76588 to 149120) | 9022.03 (6330.77 to 12417.36) | 0.31 (0.12 to 0.49) |
| Oman | 25355 (16617 to 37297) | 7675.64 (5065.46 to 11239.05) | 95445 (58693 to 147853) | 9332.76 (5734.78 to 14444.06) | 0.21 (0 to 0.42) |
| Pakistan | 1504176 (1060839 to 2069926) | 6849.92 (4856.6 to 9368.96) | 4383429 (3015678 to 6135290) | 7492.72 (5179.46 to 10439.18) | 0.01 (-0.19 to 0.21) |
| Palau | 163 (106 to 250) | 3996.71 (2590.4 to 6079.69) | 173 (106 to 272) | 4626.5 (2824.34 to 7360.24) | 0.12 (0.03 to 0.22) |
| Palestine | 47429 (31145 to 70101) | 11149.71 (7414.35 to 16287.39) | 165980 (103743 to 255284) | 12928.12 (8113.95 to 19762.78) | 0.03 (-0.17 to 0.23) |
| Panama | 29845 (19890 to 44153) | 5044.94 (3372.39 to 7421.16) | 70234 (43887 to 108810) | 6560.25 (4096.23 to 10164.68) | 0.31 (0.03 to 0.58) |
| Papua New Guinea | 40460 (27636 to 58314) | 4149.45 (2849.66 to 5923.22) | 108321 (69910 to 162876) | 4124.73 (2666.96 to 6188.47) | -0.22 (-0.28 to -0.17) |
| Paraguay | 58987 (39924 to 86147) | 6337.03 (4306.93 to 9189.43) | 172091 (109768 to 257094) | 9064.1 (5787.36 to 13516.77) | 0.48 (0.27 to 0.7) |
| Peru | 195569 (131915 to 280703) | 3703.19 (2510.32 to 5289.96) | 462755 (294176 to 697367) | 4791.79 (3044.95 to 7222.38) | -0.21 (-0.66 to 0.25) |
| Philippines | 583328 (415933 to 802958) | 3851.5 (2769.33 to 5258.1) | 1310788 (925260 to 1794751) | 4480.85 (3170.63 to 6115.64) | -0.16 (-0.44 to 0.13) |
| Poland | 258564 (186853 to 348341) | 2716.88 (1965.12 to 3662.61) | 311864 (222152 to 420482) | 3416.86 (2419.65 to 4657.49) | -0.35 (-0.75 to 0.05) |
| Portugal | 288592 (199566 to 397385) | 11409.26 (7885.53 to 15717.8) | 304202 (197707 to 456385) | 13053.77 (8371.18 to 19763.49) | -0.48 (-0.83 to -0.14) |
| Puerto Rico | 40050 (27253 to 56848) | 4185.4 (2850.09 to 5938.85) | 39468 (25059 to 59748) | 5188.72 (3271.74 to 7898.84) | 0.17 (-0.09 to 0.42) |
| Qatar | 7168 (4831 to 10191) | 9037.63 (6098.97 to 12807.31) | 52235 (32474 to 79659) | 9152.24 (5647.75 to 14060.42) | -0.34 (-0.49 to -0.18) |
| Republic of Korea | 472254 (359947 to 620642) | 3683.99 (2810.92 to 4832.65) | 507502 (347213 to 711025) | 4521.32 (3067.71 to 6360.41) | 0.41 (0.26 to 0.56) |
| Republic of Moldova | 64623 (45668 to 88347) | 5732.93 (4052.84 to 7835.31) | 58334 (38131 to 87375) | 6324.9 (4077.36 to 9560.63) | -0.56 (-0.86 to -0.25) |
| Romania | 222272 (153256 to 311011) | 3947.8 (2726.37 to 5528.55) | 221116 (144871 to 326534) | 5298.94 (3426.71 to 7924.2) | 0.06 (-0.25 to 0.38) |
| Russian Federation | 1774637 (1271924 to 2421810) | 4727.57 (3387.74 to 6445.91) | 2212682 (1576615 to 2998865) | 6361.07 (4493.17 to 8707.37) | 0.1 (-0.24 to 0.44) |
| Rwanda | 143936 (96641 to 208155) | 9457.59 (6421.01 to 13497.28) | 321520 (200706 to 484540) | 9386.64 (5892.59 to 14061.05) | -0.85 (-1.07 to -0.63) |
| Saint Kitts and Nevis | 666 (420 to 1033) | 6955.74 (4418.5 to 10699.38) | 1379 (822 to 2202) | 8676.35 (5159.86 to 13903.22) | 0.24 (0.07 to 0.41) |
| Saint Lucia | 1765 (1167 to 2618) | 5354.36 (3564.16 to 7881.92) | 3472 (2179 to 5287) | 7524.26 (4697.34 to 11492.27) | 0.34 (0.06 to 0.62) |
| Saint Vincent and the Grenadines | 1346 (884 to 1996) | 5325.84 (3529.55 to 7832.03) | 1983 (1248 to 3042) | 7082.98 (4448.9 to 10887) | 0.33 (0.09 to 0.57) |
| Samoa | 1274 (882 to 1801) | 3425.13 (2400.27 to 4799.25) | 1775 (1155 to 2658) | 3630.01 (2377.9 to 5408.67) | -0.22 (-0.34 to -0.09) |
| San Marino | 523 (333 to 806) | 8390.97 (5344.07 to 12913.41) | 784 (472 to 1244) | 11099.34 (6632.41 to 17766.93) | 0.34 (0.1 to 0.59) |
| Sao Tome and Principe | 1384 (919 to 2008) | 5710.88 (3842.38 to 8204.18) | 3419 (2149 to 5211) | 6264.1 (3964.62 to 9505.49) | 0.13 (0 to 0.25) |
| Saudi Arabia | 244937 (158767 to 364731) | 7763.08 (5080.87 to 11454.47) | 930536 (582951 to 1419741) | 9053.13 (5688.05 to 13810.61) | 0.34 (0.17 to 0.51) |
| Senegal | 87361 (59297 to 127558) | 5386.08 (3702.25 to 7792.83) | 245286 (157596 to 373258) | 6525.31 (4229.93 to 9856.89) | 0.19 (-0.02 to 0.39) |
| Serbia | 113688 (81568 to 154984) | 4818.57 (3448.93 to 6586.16) | 111759 (72675 to 167919) | 5346.2 (3447.84 to 8088.42) | -0.49 (-0.75 to -0.22) |
| Seychelles | 515 (349 to 735) | 2884.06 (1979.3 to 4071.33) | 920 (575 to 1399) | 3800.17 (2360.31 to 5814.33) | 0.1 (-0.17 to 0.37) |
| Sierra Leone | 61356 (41095 to 88491) | 6419.11 (4353.51 to 9153.53) | 152450 (97301 to 233480) | 7101.63 (4585.67 to 10802.79) | 0.39 (0.31 to 0.46) |
| Singapore | 61252 (48033 to 79199) | 6469.95 (5068.27 to 8369.84) | 56366 (38517 to 80565) | 4201.22 (2848.65 to 6054.3) | -2.2 (-2.57 to -1.83) |
| Slovakia | 59168 (41494 to 82498) | 4431.95 (3104.06 to 6192.74) | 74994 (48097 to 113785) | 5749.76 (3655.23 to 8819.82) | -0.17 (-0.53 to 0.2) |
| Slovenia | 32955 (23993 to 45107) | 6561.32 (4769.27 to 8999.98) | 27420 (18274 to 40308) | 6308.26 (4157.48 to 9356.59) | -0.96 (-1.23 to -0.68) |
| Solomon Islands | 2978 (2040 to 4282) | 3980.18 (2756.94 to 5673.71) | 7205 (4677 to 10830) | 4193.92 (2732.19 to 6280.51) | -0.22 (-0.32 to -0.11) |
| Somalia | 120315 (81221 to 172132) | 7410.09 (5042.15 to 10535.81) | 430854 (270558 to 653330) | 9310.93 (5904.35 to 14002.06) | 0.11 (-0.07 to 0.28) |
| South Africa | 672596 (512761 to 876301) | 7295.43 (5599.36 to 9443.19) | 1479857 (1111593 to 1949798) | 9495.58 (7127.29 to 12514.1) | 0.43 (0.12 to 0.74) |
| South Sudan | 85317 (58104 to 123423) | 7054.65 (4875.64 to 10077.4) | 171157 (108700 to 262954) | 7674.61 (4909.01 to 11744.75) | 0.09 (-0.02 to 0.19) |
| Spain | 784653 (642198 to 958788) | 8145.56 (6669.62 to 9948.59) | 1242554 (827795 to 1774338) | 12605.74 (8310.49 to 18246.4) | 1.52 (1.09 to 1.95) |
| Sri Lanka | 229062 (166689 to 306537) | 4957.52 (3618.11 to 6622.27) | 260591 (174277 to 375517) | 4659.68 (3111.22 to 6723.81) | -1.18 (-1.56 to -0.79) |
| Sudan | 380969 (254091 to 555564) | 8271.09 (5572.58 to 11964.18) | 1014634 (641738 to 1548617) | 9078.98 (5777.34 to 13814.97) | -0.19 (-0.37 to 0) |
| Suriname | 8204 (5662 to 11444) | 8501.93 (5895.97 to 11805.7) | 18315 (12083 to 26938) | 12614.94 (8313.4 to 18575.15) | 0.41 (0.17 to 0.65) |
| Sweden | 206569 (155185 to 267013) | 9978.83 (7452.53 to 12938.8) | 264211 (188275 to 360841) | 12110.76 (8562.56 to 16591.38) | 0.1 (-0.09 to 0.29) |
| Switzerland | 164221 (120376 to 220109) | 9290.65 (6759.2 to 12489.64) | 183468 (120463 to 271077) | 9228.46 (6015.91 to 13664.3) | -0.87 (-1.36 to -0.37) |
| Syrian Arab Republic | 202577 (131521 to 302647) | 7631.03 (5002 to 11298.28) | 353314 (218986 to 542690) | 9123.93 (5670.47 to 14052.13) | 0.16 (-0.05 to 0.36) |
| Taiwan (Province of China) | 135788 (96842 to 189107) | 2504.37 (1791.38 to 3473.82) | 180125 (122940 to 257490) | 3058.11 (2055.78 to 4421.2) | 0.57 (0.52 to 0.63) |
| Tajikistan | 50365 (34266 to 72653) | 4360.19 (3000.41 to 6207) | 131816 (83785 to 198765) | 5232.75 (3328.68 to 7858.75) | -0.11 (-0.37 to 0.14) |
| Thailand | 569854 (401745 to 784420) | 3562.37 (2525.34 to 4880.04) | 646214 (431570 to 955466) | 3877.74 (2561.76 to 5761.46) | -0.27 (-0.41 to -0.14) |
| Timor-Leste | 7405 (5119 to 10655) | 3964.39 (2757.97 to 5664.41) | 13704 (8816 to 20537) | 3924.64 (2548.53 to 5841.05) | -0.73 (-0.97 to -0.5) |
| Togo | 53389 (36269 to 76635) | 6636.3 (4567.39 to 9372.69) | 147941 (93817 to 220412) | 7031.19 (4487.14 to 10429.25) | 0.02 (-0.07 to 0.1) |
| Tokelau | 14 (9 to 21) | 3996.78 (2578.95 to 6027.39) | 15 (9 to 24) | 4603.13 (2804.37 to 7457.53) | 0.12 (0.02 to 0.21) |
| Tonga | 739 (516 to 1030) | 3271.03 (2299.93 to 4530.76) | 903 (593 to 1355) | 3547.69 (2334.96 to 5312.05) | -0.11 (-0.23 to 0.01) |
| Trinidad and Tobago | 22798 (15712 to 32127) | 7448.38 (5149.74 to 10461.98) | 32707 (21037 to 49547) | 9624.7 (6190.97 to 14576.45) | -0.14 (-0.43 to 0.16) |
| Tunisia | 188815 (124201 to 275346) | 9488.41 (6296.39 to 13749) | 394079 (246800 to 599833) | 12741.92 (7963.54 to 19504.28) | 0.15 (-0.13 to 0.44) |
| Turkey | 1144120 (896719 to 1447779) | 8160.31 (6424.61 to 10285.35) | 2144474 (1340093 to 3310450) | 9883.9 (6161.24 to 15273.07) | 0.2 (0.01 to 0.39) |
| Turkmenistan | 39831 (27601 to 56422) | 4653.35 (3256.96 to 6530) | 67451 (42867 to 102586) | 5372.26 (3414.06 to 8168.81) | -0.1 (-0.31 to 0.1) |
| Tuvalu | 97 (62 to 146) | 3967.59 (2565.93 to 5996.1) | 133 (81 to 207) | 4556.94 (2778.74 to 7096.99) | 0.12 (0.03 to 0.21) |
| Uganda | 382948 (261178 to 547848) | 10712.63 (7403.84 to 15122.87) | 1161660 (734253 to 1751486) | 11746.52 (7523.46 to 17565.04) | -0.52 (-0.83 to -0.21) |
| Ukraine | 884897 (628766 to 1219316) | 6846.73 (4854.54 to 9453.38) | 902059 (606586 to 1294976) | 8414.91 (5606.07 to 12112.71) | -0.5 (-0.77 to -0.22) |
| United Arab Emirates | 26332 (17862 to 38376) | 7854.95 (5369.6 to 11338.88) | 156004 (99376 to 236151) | 8526.54 (5378.82 to 13047.89) | -0.33 (-0.55 to -0.1) |
| United Kingdom | 1327501 (966462 to 1797543) | 9315.39 (6767.09 to 12626.22) | 1592591 (1152943 to 2150551) | 10342.25 (7445.83 to 14054.11) | -0.13 (-0.49 to 0.23) |
| United Republic of Tanzania | 394745 (268438 to 574645) | 7010.35 (4829.99 to 10099.83) | 1093172 (688853 to 1649976) | 7641.62 (4866.57 to 11461.41) | -0.13 (-0.26 to 0) |
| United States of America | 4796588 (3729261 to 6154793) | 7213.07 (5590.43 to 9278.62) | 9742035 (7582373 to 12323151) | 13072.89 (10148.83 to 16567.53) | 0.87 (0.51 to 1.23) |
| United States Virgin Islands | 1565 (1041 to 2304) | 5403.05 (3589.21 to 7989.07) | 1207 (769 to 1793) | 6931.32 (4399.08 to 10377.58) | 0.32 (0.11 to 0.53) |
| Uruguay | 44469 (30584 to 63351) | 5923.73 (4073.14 to 8441.73) | 74278 (48950 to 109064) | 8980.01 (5900.93 to 13210.89) | 0.95 (0.72 to 1.19) |
| Uzbekistan | 228256 (158663 to 320754) | 4829.59 (3391.91 to 6726.56) | 477721 (310172 to 713490) | 5359.03 (3477.34 to 8010.52) | -0.15 (-0.32 to 0.03) |
| Vanuatu | 1379 (958 to 1969) | 3906.05 (2730.67 to 5552.27) | 3247 (2101 to 4896) | 4111.52 (2671 to 6173.34) | -0.23 (-0.35 to -0.12) |
| Venezuela (Bolivarian Republic of) | 251487 (168051 to 369348) | 5389.74 (3626.29 to 7838.34) | 424740 (270549 to 646885) | 6080.45 (3869.75 to 9279.43) | 0.07 (-0.06 to 0.19) |
| Viet Nam | 562912 (393565 to 781857) | 3323.51 (2345.88 to 4579.71) | 915667 (604468 to 1349456) | 3611.83 (2367.49 to 5348.51) | -0.42 (-0.6 to -0.23) |
| Yemen | 274296 (182878 to 397464) | 10228.77 (6884.55 to 14719.87) | 833589 (519303 to 1257355) | 10178.37 (6386.74 to 15268.16) | -0.14 (-0.2 to -0.07) |
| Zambia | 97805 (66640 to 140300) | 5724.12 (3958.31 to 8132.4) | 314535 (201694 to 473940) | 6663.73 (4313.99 to 9954.08) | -0.12 (-0.29 to 0.05) |
| Zimbabwe | 93309 (62653 to 135389) | 4168.63 (2827.54 to 5980.68) | 202752 (129581 to 308792) | 5148.42 (3311.47 to 7791.5) | 0.19 (0 to 0.38) |

**Supplementary Table S5** The prevalence of depressive disorders cases and rates among WCBA in 204 countries and territories in 1990 and 2021, and the trends from 1990 to 2021.

| Location | Prevalence | | | | |
| --- | --- | --- | --- | --- | --- |
|  | No.,1990 (95% UI) | ASPR, 1990 per 100,000 (95% UI) | No.,2021, (95% UI) | ASPR, 2021 per 100,000 (95% UI) | EAPC, 1990-2021, (95% CI) |
| Afghanistan | 168978 (121704 to 231442) | 8161.04 (5956.71 to 11094.95) | 650095 (446660 to 913967) | 9473.15 (6571.78 to 13211.84) | 0.1 (-0.03 to 0.23) |
| Albania | 31762 (23908 to 41934) | 3918.81 (2962.84 to 5149.96) | 31188 (22620 to 42841) | 5007.92 (3617.29 to 6901.25) | 0.26 (0.09 to 0.44) |
| Algeria | 386859 (275388 to 531927) | 7005.5 (5041.97 to 9552.43) | 860104 (600477 to 1229925) | 7546.09 (5247.49 to 10831.37) | 0.03 (-0.11 to 0.16) |
| American Samoa | 428 (321 to 565) | 3731.67 (2814.92 to 4927.84) | 469 (345 to 630) | 3996.43 (2949.59 to 5364.64) | 0.03 (-0.04 to 0.1) |
| Andorra | 986 (733 to 1326) | 6544.43 (4837.52 to 8820.05) | 1566 (1095 to 2200) | 7814.89 (5386.18 to 11110.14) | 0.13 (-0.09 to 0.35) |
| Angola | 207226 (149873 to 281648) | 9394.68 (6839.44 to 12691.61) | 750600 (525849 to 1063561) | 10169.56 (7161.37 to 14313.7) | -0.02 (-0.12 to 0.08) |
| Antigua and Barbuda | 736 (527 to 1007) | 4617.43 (3310.74 to 6290.62) | 1391 (967 to 1977) | 5631.91 (3898.74 to 8051.56) | 0.21 (0 to 0.41) |
| Argentina | 375202 (274466 to 500915) | 4681.76 (3429.54 to 6247.08) | 656162 (486414 to 863654) | 5550.91 (4107.02 to 7319.81) | -0.14 (-0.37 to 0.1) |
| Armenia | 34406 (25728 to 45435) | 4046.77 (3028.82 to 5329.02) | 38840 (27858 to 52828) | 5121.94 (3639.68 to 7025.7) | 0.28 (0.08 to 0.48) |
| Australia | 347655 (276660 to 430056) | 7791.5 (6192.07 to 9651.28) | 519833 (376985 to 706463) | 8716.12 (6286.32 to 11906.4) | 0.21 (0.05 to 0.37) |
| Austria | 125498 (95499 to 162639) | 6285.7 (4767.85 to 8148.48) | 126193 (90740 to 173557) | 6358.81 (4527.09 to 8813.61) | -0.49 (-0.72 to -0.27) |
| Azerbaijan | 72645 (54289 to 96516) | 4036.1 (3029.66 to 5331.07) | 138362 (98958 to 186913) | 4936.89 (3508.75 to 6702.87) | 0.21 (0.02 to 0.4) |
| Bahamas | 3281 (2371 to 4497) | 4648.31 (3365.79 to 6328.94) | 6317 (4330 to 9099) | 5811.59 (3979.1 to 8392.37) | 0.25 (0.03 to 0.48) |
| Bahrain | 9523 (6899 to 12916) | 8410.9 (6115.19 to 11345.09) | 29291 (20348 to 41547) | 8908.9 (6171.2 to 12654.85) | -0.36 (-0.52 to -0.19) |
| Bangladesh | 1779731 (1316718 to 2394184) | 7697.68 (5733.9 to 10271.28) | 3801695 (2668852 to 5274271) | 8358.88 (5873.79 to 11584.21) | -0.04 (-0.17 to 0.09) |
| Barbados | 3136 (2263 to 4247) | 4613.06 (3325.78 to 6230.84) | 4304 (2993 to 6140) | 5921.62 (4096.1 to 8496.49) | 0.28 (0.09 to 0.48) |
| Belarus | 142909 (108148 to 187529) | 5577.87 (4215.05 to 7324.49) | 174829 (124476 to 239270) | 7778.96 (5494.84 to 10748.15) | 0.19 (-0.03 to 0.42) |
| Belgium | 138855 (116941 to 165152) | 5663.05 (4763.32 to 6750.3) | 183585 (131770 to 252488) | 7323.57 (5213.02 to 10119.69) | 0.71 (0.52 to 0.9) |
| Belize | 1861 (1349 to 2572) | 4699.72 (3428.77 to 6406.75) | 6852 (4751 to 9699) | 5750.78 (4000.53 to 8113.72) | 0.31 (0.15 to 0.48) |
| Benin | 64513 (47995 to 86762) | 6242.35 (4681.58 to 8315.2) | 204198 (143276 to 282881) | 6679.72 (4723.81 to 9185.65) | 0.07 (0.01 to 0.12) |
| Bermuda | 944 (699 to 1275) | 5419.36 (4001.2 to 7334.36) | 815 (559 to 1143) | 5853.94 (3977.25 to 8303.55) | -0.34 (-0.55 to -0.12) |
| Bhutan | 7722 (5725 to 10331) | 6043.72 (4506.91 to 7999.12) | 11586 (8242 to 15837) | 5671.33 (4036.64 to 7737.32) | -0.26 (-0.36 to -0.17) |
| Bolivia (Plurinational State of) | 82362 (60619 to 111747) | 5634.17 (4172.42 to 7586.39) | 219701 (152125 to 311322) | 7081.79 (4907.28 to 10012.75) | 0.02 (-0.29 to 0.34) |
| Bosnia and Herzegovina | 60177 (45367 to 79406) | 5181.06 (3906.61 to 6834.62) | 41040 (29010 to 56913) | 5407.21 (3804.32 to 7547.03) | -0.62 (-0.84 to -0.4) |
| Botswana | 18383 (13535 to 24349) | 6172.09 (4572.13 to 8123.19) | 52180 (36909 to 72318) | 7665.49 (5420.01 to 10613.12) | 0.12 (-0.01 to 0.26) |
| Brazil | 2539487 (2003337 to 3192922) | 6702.03 (5315.13 to 8376.01) | 4589446 (3587264 to 5764568) | 7641.53 (5955.81 to 9640.5) | -0.38 (-0.7 to -0.06) |
| Brunei Darussalam | 1896 (1397 to 2550) | 2776.01 (2047.61 to 3716.01) | 3949 (2795 to 5539) | 3155.59 (2225.67 to 4444.72) | 0.13 (-0.03 to 0.3) |
| Bulgaria | 95902 (73275 to 124423) | 4534.24 (3455.69 to 5897.07) | 82529 (59771 to 112213) | 5548.22 (3972.58 to 7631.19) | -0.26 (-0.52 to -0.01) |
| Burkina Faso | 120087 (89852 to 159836) | 6067.23 (4568.33 to 8017.89) | 318506 (229250 to 443236) | 6163.96 (4461.83 to 8494.81) | 0.26 (0.16 to 0.35) |
| Burundi | 92414 (68226 to 122651) | 7765.25 (5749.57 to 10234.09) | 222613 (158010 to 308677) | 7498.07 (5367.72 to 10333.36) | -0.55 (-0.7 to -0.41) |
| Cabo Verde | 4444 (3280 to 5945) | 6087.41 (4541.52 to 8070.1) | 11249 (7818 to 15767) | 7559.99 (5257.1 to 10573.62) | 0.31 (0.18 to 0.45) |
| Cambodia | 113723 (84698 to 151369) | 4714.04 (3537.5 to 6241.97) | 212654 (154269 to 283845) | 4749.02 (3444.96 to 6340.18) | -0.52 (-0.65 to -0.39) |
| Cameroon | 144374 (107234 to 193506) | 6474.24 (4837.29 to 8612.99) | 512696 (364388 to 712535) | 6911.45 (4951.74 to 9536.96) | 0.05 (-0.01 to 0.1) |
| Canada | 459018 (364332 to 570259) | 6193.06 (4895.3 to 7724.02) | 653782 (471031 to 883069) | 7941.77 (5673.3 to 10818.18) | 0.08 (-0.16 to 0.31) |
| Central African Republic | 57570 (41853 to 78750) | 9340.04 (6828.01 to 12650.73) | 130048 (89402 to 184200) | 9760.41 (6770.57 to 13773.02) | 0.02 (-0.07 to 0.11) |
| Chad | 91621 (67282 to 122338) | 7317.49 (5421.07 to 9701.62) | 272088 (189312 to 382497) | 7618.46 (5360.17 to 10588.78) | 0.08 (0.01 to 0.14) |
| Chile | 298422 (243298 to 365948) | 8208.27 (6700.05 to 10044.34) | 413411 (282369 to 586505) | 8851.05 (6015.54 to 12584.37) | -0.36 (-0.6 to -0.12) |
| China | 14618427 (11803208 to 17897663) | 4724.65 (3835.87 to 5756.45) | 12975936 (10596507 to 15717771) | 3695.55 (2997.95 to 4502.63) | -0.96 (-1.12 to -0.81) |
| Colombia | 285920 (209301 to 383689) | 3396 (2489.58 to 4533.77) | 424889 (305754 to 573530) | 3221.5 (2316.72 to 4351.35) | -0.79 (-1.1 to -0.48) |
| Comoros | 6101 (4544 to 8055) | 6166.41 (4633.37 to 8092.71) | 12887 (9240 to 17749) | 6712.16 (4820.32 to 9217.44) | -0.04 (-0.15 to 0.07) |
| Congo | 49611 (35893 to 67417) | 9332.96 (6819.95 to 12565.17) | 139328 (96858 to 198209) | 9901.89 (6909.19 to 14048.29) | -0.12 (-0.27 to 0.04) |
| Cook Islands | 196 (141 to 271) | 4421.24 (3213.12 to 6079.56) | 209 (146 to 297) | 4829.23 (3382.31 to 6852.3) | 0.07 (0.02 to 0.13) |
| Costa Rica | 34441 (24894 to 47388) | 4580.57 (3320.17 to 6272.15) | 77015 (53024 to 110764) | 5853.77 (4024.79 to 8431.54) | 0.38 (0.23 to 0.54) |
| Coted'Ivoire | 144681 (107792 to 192058) | 5666.46 (4259.8 to 7468.69) | 371740 (267731 to 505027) | 5818.12 (4207.91 to 7870.6) | 0 (-0.07 to 0.06) |
| Croatia | 62538 (47591 to 80617) | 5097.65 (3871.23 to 6589.63) | 47753 (34620 to 64381) | 5091.08 (3663.41 to 6921.08) | -0.57 (-0.77 to -0.37) |
| Cuba | 246679 (186059 to 317816) | 8104.51 (6125.94 to 10408.93) | 167300 (118310 to 235051) | 6489.07 (4551.95 to 9194.29) | -1.56 (-1.87 to -1.25) |
| Cyprus | 11196 (8152 to 15132) | 5651.6 (4108.64 to 7645.1) | 23450 (16450 to 32687) | 6564.33 (4557.58 to 9240.27) | 0.15 (-0.03 to 0.32) |
| Czechia | 132450 (101941 to 171366) | 5025.83 (3856.61 to 6527.53) | 128658 (92182 to 173598) | 5283.08 (3746.89 to 7221.84) | -0.47 (-0.66 to -0.27) |
| Democratic People's Republic of Korea | 223317 (168249 to 293159) | 4032.36 (3045.06 to 5281.03) | 264156 (195154 to 352489) | 3860.68 (2843.12 to 5159.24) | -0.3 (-0.35 to -0.25) |
| Democratic Republic of the Congo | 720614 (527988 to 967386) | 8817.25 (6517.12 to 11732.38) | 1849042 (1293953 to 2565884) | 9030.64 (6365.71 to 12452.1) | -0.03 (-0.11 to 0.06) |
| Denmark | 105814 (80291 to 137954) | 7940.24 (6004.47 to 10378.62) | 92011 (65278 to 127371) | 7208.71 (5079.54 to 10014.49) | -0.82 (-1.07 to -0.57) |
| Djibouti | 5899 (4339 to 7943) | 6380.83 (4737.52 to 8529.92) | 22626 (16338 to 30862) | 7035.72 (5080.09 to 9588.88) | 0.09 (0 to 0.18) |
| Dominica | 754 (539 to 1044) | 4628.86 (3324.74 to 6368.69) | 942 (647 to 1350) | 5728.76 (3931.79 to 8219.2) | 0.22 (0.02 to 0.42) |
| Dominican Republic | 104026 (74217 to 144232) | 5789.03 (4162.6 to 7952.77) | 192312 (129503 to 277975) | 6654.06 (4482.49 to 9610.36) | 0.13 (-0.03 to 0.3) |
| Ecuador | 124090 (90751 to 165720) | 5042.96 (3709.29 to 6701.42) | 300053 (208391 to 416674) | 6352.78 (4417.6 to 8814.19) | 0.21 (-0.04 to 0.46) |
| Egypt | 816658 (586302 to 1126283) | 6400.57 (4615.66 to 8776.5) | 1949841 (1339613 to 2780066) | 7572.92 (5215.41 to 10767.38) | 0.21 (0.04 to 0.38) |
| El Salvador | 79822 (58255 to 107558) | 6240.84 (4582.15 to 8369.01) | 114767 (80515 to 160971) | 6439.69 (4518.43 to 9026.33) | -0.36 (-0.56 to -0.16) |
| Equatorial Guinea | 8971 (6567 to 12266) | 9461.27 (6958.47 to 12840.23) | 35022 (24282 to 49530) | 9912.19 (6898.55 to 13934.88) | 0.02 (-0.08 to 0.12) |
| Eritrea | 52036 (38718 to 69064) | 6970.63 (5218.42 to 9192) | 116599 (83675 to 160286) | 7272.31 (5240.24 to 9943.64) | -0.06 (-0.14 to 0.01) |
| Estonia | 26704 (20289 to 34463) | 6844.37 (5186.94 to 8852.13) | 19343 (13882 to 26830) | 6615.42 (4696.72 to 9277.4) | -1.02 (-1.25 to -0.79) |
| Eswatini | 11055 (8182 to 14689) | 6121.72 (4567.95 to 8071.72) | 26894 (18870 to 37614) | 8757.22 (6163.63 to 12204.74) | 0.48 (0.28 to 0.68) |
| Ethiopia | 728809 (574731 to 922583) | 6870.39 (5436.31 to 8641.01) | 1787212 (1390339 to 2302490) | 6820.36 (5345.69 to 8728.26) | -0.33 (-0.47 to -0.2) |
| Fiji | 7810 (5882 to 10212) | 4112.11 (3103.6 to 5366.98) | 10582 (7726 to 14292) | 4632.31 (3382.45 to 6256.51) | 0.01 (-0.08 to 0.11) |
| Finland | 111548 (84906 to 143173) | 8707.44 (6584.75 to 11219.88) | 104147 (74880 to 140771) | 9212.66 (6578.93 to 12522.56) | -0.4 (-0.59 to -0.2) |
| France | 1151966 (982126 to 1346311) | 7926.84 (6749.16 to 9273.48) | 1192021 (850437 to 1652953) | 8290.03 (5877.04 to 11549.82) | -0.3 (-0.49 to -0.11) |
| Gabon | 18107 (13180 to 24855) | 8616.27 (6333.64 to 11704.2) | 45038 (31251 to 62760) | 9434.54 (6589.3 to 13076.96) | -0.07 (-0.18 to 0.05) |
| Gambia | 20301 (14821 to 27917) | 9685.39 (7133.19 to 13140.04) | 58241 (39766 to 82885) | 10074.23 (6948.41 to 14192.82) | -0.4 (-0.56 to -0.25) |
| Georgia | 59941 (44941 to 79466) | 4366.77 (3269.12 to 5794.01) | 43984 (31177 to 60627) | 5382.96 (3780.63 to 7474.98) | 0.16 (-0.01 to 0.34) |
| Germany | 1019809 (827765 to 1258053) | 5195.82 (4205.49 to 6427.45) | 1192126 (842973 to 1656923) | 6976.31 (4886.2 to 9769.63) | 0.89 (0.76 to 1.03) |
| Ghana | 208165 (153316 to 276984) | 6241.52 (4625.72 to 8243) | 591397 (416683 to 826311) | 6650.32 (4702 to 9257.98) | -0.02 (-0.1 to 0.06) |
| Greece | 219757 (155377 to 305611) | 8687.44 (6132.18 to 12095.36) | 235558 (158129 to 340473) | 10839 (7231.31 to 15717.3) | 0 (-0.34 to 0.34) |
| Greenland | 2110 (1587 to 2780) | 13879.12 (10446.14 to 18267.87) | 1990 (1424 to 2713) | 15623.41 (11143.73 to 21322.5) | -0.31 (-0.51 to -0.11) |
| Grenada | 877 (633 to 1209) | 4705.98 (3413.9 to 6434.89) | 1472 (1017 to 2071) | 5719.57 (3944.64 to 8043.12) | 0.2 (0.04 to 0.35) |
| Guam | 1437 (1080 to 1877) | 4190.19 (3155.91 to 5473.5) | 1739 (1290 to 2354) | 4779.61 (3539.59 to 6475.18) | 0.13 (0.01 to 0.26) |
| Guatemala | 96002 (69369 to 133908) | 5624.88 (4093.61 to 7757.94) | 289719 (195707 to 425603) | 6760.51 (4587.59 to 9864.61) | 0.04 (-0.17 to 0.25) |
| Guinea | 83562 (62141 to 111447) | 6362.05 (4757.1 to 8436.84) | 219123 (155795 to 303250) | 6965.24 (4991.99 to 9575.81) | 0.09 (0.01 to 0.17) |
| Guinea-Bissau | 14224 (10580 to 18961) | 6516.84 (4885.46 to 8628.18) | 35513 (25251 to 49115) | 7102.2 (5076.76 to 9768.35) | 0.12 (0.04 to 0.19) |
| Guyana | 13947 (10158 to 18615) | 7016.57 (5142.12 to 9320.16) | 20281 (13822 to 28828) | 9955.92 (6789.44 to 14124.61) | 0.55 (0.38 to 0.72) |
| Haiti | 86694 (62663 to 119823) | 5809.79 (4222.6 to 7986.28) | 230963 (159035 to 326285) | 6571.49 (4534.8 to 9263.48) | -0.02 (-0.16 to 0.13) |
| Honduras | 44163 (31860 to 60545) | 4492.03 (3261.49 to 6099.22) | 161817 (108665 to 234280) | 5847.55 (3945.2 to 8426.18) | 0.31 (0.11 to 0.52) |
| Hungary | 136170 (105055 to 174560) | 5199.55 (3998.77 to 6687.35) | 111480 (82022 to 150030) | 4858.84 (3531.86 to 6601.04) | -0.8 (-0.99 to -0.62) |
| Iceland | 3830 (2879 to 5018) | 5892.28 (4427.92 to 7715.35) | 4610 (3297 to 6301) | 5775.34 (4108.17 to 7922.58) | -0.36 (-0.5 to -0.21) |
| India | 11776150 (9319416 to 14713282) | 6094.34 (4842.23 to 7573.36) | 22836700 (18112196 to 28489254) | 6089.46 (4834.93 to 7583.28) | -0.91 (-1.19 to -0.64) |
| Indonesia | 1624374 (1285651 to 2015013) | 3576.57 (2846.85 to 4412.35) | 3189281 (2533118 to 3974792) | 4171.85 (3305.93 to 5211.81) | 0.12 (-0.01 to 0.26) |
| Iran (Islamic Republic of) | 1060506 (800309 to 1407705) | 8687.9 (6609.36 to 11421.04) | 2353352 (1778512 to 3072732) | 9946.24 (7478.66 to 13076.52) | 0.51 (0.33 to 0.7) |
| Iraq | 262737 (193563 to 350796) | 6821.52 (5080.75 to 9021.33) | 755613 (531380 to 1043004) | 7296.48 (5148.34 to 10031.24) | 0.15 (-0.23 to 0.54) |
| Ireland | 60034 (46536 to 77191) | 6832.45 (5305.29 to 8776.64) | 106407 (74490 to 148239) | 9232.8 (6425.69 to 12915.92) | 0.4 (0.19 to 0.61) |
| Israel | 90676 (66902 to 122529) | 7464.54 (5521.41 to 10066.31) | 184413 (127137 to 259898) | 8291.44 (5709.1 to 11701.82) | -0.29 (-0.47 to -0.11) |
| Italy | 949551 (730045 to 1226237) | 6614.35 (5081.03 to 8546.8) | 989535 (758520 to 1283659) | 8119.67 (6171.39 to 10628.79) | -0.21 (-0.49 to 0.08) |
| Jamaica | 26515 (18936 to 36589) | 4671.98 (3348.89 to 6391.81) | 45314 (30695 to 65767) | 5842.13 (3952.44 to 8479.08) | 0.21 (0 to 0.42) |
| Japan | 1104898 (903192 to 1339119) | 3468.73 (2827.1 to 4217.42) | 1088945 (884682 to 1334521) | 4470.53 (3597.43 to 5528.13) | 0.37 (0.14 to 0.61) |
| Jordan | 61056 (43579 to 84369) | 7843.86 (5686.08 to 10697.4) | 244693 (167831 to 348910) | 8009.33 (5516.44 to 11383.63) | -0.31 (-0.44 to -0.18) |
| Kazakhstan | 192890 (146865 to 250462) | 4771.17 (3635.65 to 6190.99) | 270337 (194071 to 364814) | 5622.33 (4020.04 to 7619.8) | 0.34 (0.21 to 0.47) |
| Kenya | 328833 (260939 to 411614) | 6953.88 (5566.11 to 8609.85) | 893841 (705220 to 1119139) | 7078.98 (5616.55 to 8791.86) | -0.23 (-0.35 to -0.11) |
| Kiribati | 767 (576 to 1003) | 4222.57 (3184.24 to 5514.52) | 1333 (978 to 1789) | 4227.31 (3106.89 to 5661.27) | -0.19 (-0.27 to -0.12) |
| Kuwait | 28459 (20708 to 38881) | 6984 (5081.74 to 9490.11) | 106042 (74094 to 150056) | 6941.63 (4802.15 to 9923.38) | -0.13 (-0.21 to -0.04) |
| Kyrgyzstan | 49307 (37089 to 64467) | 4940.59 (3735.47 to 6425.39) | 98447 (71760 to 133308) | 5720.35 (4166.74 to 7747.17) | 0.03 (-0.13 to 0.2) |
| Lao People's Democratic Republic | 37254 (27909 to 49341) | 4025.18 (3029.25 to 5310.1) | 77282 (55373 to 104084) | 3949.06 (2833.5 to 5309.72) | -0.33 (-0.5 to -0.17) |
| Latvia | 40142 (30570 to 51960) | 5993 (4542.69 to 7784.78) | 28130 (19674 to 39074) | 6786.38 (4695.81 to 9508.96) | -0.35 (-0.55 to -0.14) |
| Lebanon | 51112 (38454 to 67679) | 6940.77 (5231.96 to 9161.73) | 154539 (105690 to 220250) | 10271.29 (6980.06 to 14684.74) | 0.39 (0.18 to 0.61) |
| Lesotho | 32491 (23825 to 43646) | 8911.22 (6561.44 to 11927.34) | 54736 (37902 to 76158) | 11151.88 (7761.43 to 15442.99) | 0.33 (0.12 to 0.54) |
| Liberia | 39961 (29318 to 53616) | 7674.22 (5678.95 to 10214.06) | 105930 (74270 to 147444) | 7922.18 (5596.75 to 10983.49) | 0.37 (0.15 to 0.58) |
| Libya | 61507 (43774 to 84700) | 7172.3 (5188.27 to 9769.09) | 160741 (110961 to 225208) | 7971.99 (5483.81 to 11190.4) | 0.09 (-0.03 to 0.22) |
| Lithuania | 57728 (43998 to 74632) | 6167.58 (4691.38 to 7988.77) | 46929 (33727 to 64042) | 7787.36 (5552.11 to 10697.02) | -0.18 (-0.42 to 0.06) |
| Luxembourg | 6785 (5153 to 8754) | 6885.99 (5199.92 to 8912.08) | 10198 (7467 to 13523) | 6519.65 (4705.45 to 8736.3) | -0.66 (-0.86 to -0.45) |
| Madagascar | 171955 (128046 to 226694) | 6794.29 (5108.18 to 8896.58) | 508943 (359888 to 699357) | 7337.4 (5206.42 to 10019.73) | -0.03 (-0.14 to 0.07) |
| Malawi | 132863 (99266 to 175712) | 6259.73 (4716.13 to 8219.04) | 314387 (225293 to 435408) | 6629.25 (4796.02 to 9092.15) | -0.22 (-0.33 to -0.11) |
| Malaysia | 186339 (139404 to 247338) | 4336.82 (3262.1 to 5732.85) | 413105 (295780 to 570061) | 4926.06 (3524.42 to 6801.28) | 0.68 (0.5 to 0.85) |
| Maldives | 2574 (1928 to 3405) | 5700.83 (4319.36 to 7448.84) | 5870 (4338 to 7882) | 5022.42 (3679.51 to 6787.41) | -0.89 (-1.04 to -0.74) |
| Mali | 99704 (74308 to 131420) | 5469.8 (4097.45 to 7160.73) | 282107 (203273 to 387031) | 5556.05 (4028.46 to 7549.73) | -0.08 (-0.18 to 0.02) |
| Malta | 5385 (3926 to 7178) | 5655 (4105.5 to 7569.99) | 6094 (4197 to 8532) | 6460.13 (4417.9 to 9093.35) | 0.16 (-0.02 to 0.35) |
| Marshall Islands | 375 (279 to 494) | 4068.15 (3052.7 to 5347.1) | 628 (462 to 852) | 4276.88 (3151.72 to 5790) | -0.07 (-0.13 to -0.01) |
| Mauritania | 22918 (17205 to 30038) | 5184.76 (3917.54 to 6753.6) | 54653 (38923 to 74638) | 5369.97 (3848.09 to 7274.17) | -0.07 (-0.17 to 0.03) |
| Mauritius | 20253 (15119 to 26715) | 6844.27 (5119.4 to 9000.83) | 23085 (16228 to 31937) | 7219.25 (5050.8 to 10015.54) | -0.41 (-0.59 to -0.24) |
| Mexico | 951302 (745420 to 1205342) | 4637.34 (3646.08 to 5827.81) | 2723620 (2094153 to 3474130) | 7715.07 (5925.12 to 9853.58) | 1.49 (1.23 to 1.74) |
| Micronesia (Federated States of) | 911 (681 to 1204) | 4154.85 (3124.09 to 5481.64) | 1088 (787 to 1462) | 4247.19 (3079 to 5684.35) | -0.12 (-0.18 to -0.06) |
| Monaco | 507 (355 to 723) | 7105.33 (4948.62 to 10173.53) | 631 (417 to 928) | 8801.84 (5812.19 to 13023.26) | 0.21 (0.05 to 0.37) |
| Mongolia | 28741 (21339 to 38500) | 5909.26 (4418.33 to 7840.87) | 50683 (37179 to 68671) | 5876.91 (4292.91 to 7984.16) | -0.22 (-0.26 to -0.18) |
| Montenegro | 6959 (5284 to 9001) | 4468.7 (3395.44 to 5778.55) | 8287 (5979 to 11324) | 5594.84 (4009.04 to 7678.43) | 0.08 (-0.11 to 0.27) |
| Morocco | 502449 (362447 to 679615) | 8280.93 (6022.98 to 11130.09) | 916598 (634812 to 1300965) | 9413.66 (6509.44 to 13375.75) | 0.02 (-0.16 to 0.2) |
| Mozambique | 201122 (149828 to 268689) | 6672.59 (4998.43 to 8870.43) | 552989 (388292 to 777537) | 7694.96 (5457.42 to 10741.32) | 0.12 (0.03 to 0.22) |
| Myanmar | 295869 (215871 to 396519) | 3038.99 (2227.21 to 4071.1) | 535792 (387690 to 735637) | 3547.4 (2569.11 to 4866.6) | 0.12 (-0.06 to 0.3) |
| Namibia | 17007 (12747 to 22228) | 5392.35 (4072.58 to 6989.77) | 43789 (31372 to 60496) | 6751.54 (4851.62 to 9290.51) | 0.01 (-0.22 to 0.24) |
| Nauru | 103 (74 to 142) | 4396.55 (3180.91 to 5999.79) | 134 (94 to 190) | 4816.41 (3388.03 to 6775.79) | 0.08 (0.03 to 0.14) |
| Nepal | 270990 (202186 to 357148) | 6319.78 (4740.51 to 8288.59) | 740819 (523102 to 1042992) | 8382.29 (5941.69 to 11747.11) | 0.5 (0.3 to 0.7) |
| Netherlands | 260278 (220274 to 308333) | 6497.93 (5494.51 to 7707.65) | 279300 (196844 to 387447) | 7570.26 (5307 to 10543.85) | -0.05 (-0.28 to 0.17) |
| New Zealand | 60171 (46012 to 78449) | 6649.54 (5082.32 to 8670.44) | 84432 (64532 to 110902) | 7170.46 (5454.64 to 9445.48) | 0.12 (0.08 to 0.16) |
| Nicaragua | 43254 (31409 to 58877) | 5020.86 (3664.99 to 6768.68) | 108542 (75706 to 152722) | 6002.92 (4189.99 to 8436.37) | 0.07 (-0.1 to 0.25) |
| Niger | 102166 (75335 to 137209) | 6280.96 (4669.63 to 8357.49) | 301918 (213941 to 411667) | 6210.13 (4455.68 to 8373.37) | -0.05 (-0.08 to -0.01) |
| Nigeria | 1111837 (867729 to 1416073) | 6013.29 (4723.45 to 7601.28) | 2881908 (2262608 to 3639433) | 5370.11 (4240.97 to 6735.03) | -0.55 (-0.68 to -0.43) |
| Niue | 22 (16 to 29) | 4402.8 (3205.54 to 6006.57) | 19 (13 to 26) | 4811.17 (3409.1 to 6742.24) | 0.07 (0.01 to 0.13) |
| North Macedonia | 21455 (16166 to 27994) | 4212.1 (3174.62 to 5495.47) | 28812 (20896 to 39619) | 5268.12 (3788.47 to 7298.18) | 0 (-0.25 to 0.25) |
| Northern Mariana Islands | 484 (358 to 645) | 3630.19 (2692.02 to 4829.83) | 501 (365 to 680) | 4251.56 (3083.98 to 5793.78) | 0.17 (0.06 to 0.29) |
| Norway | 67046 (52598 to 85377) | 6318.72 (4950.06 to 8053.16) | 92638 (70376 to 120275) | 7608.04 (5756.43 to 9921.45) | 0.23 (0.08 to 0.37) |
| Oman | 22042 (15789 to 30305) | 6735.03 (4845.95 to 9216.07) | 80509 (55001 to 115066) | 7833.77 (5325.34 to 11218.86) | 0.16 (0 to 0.32) |
| Pakistan | 1333356 (1024282 to 1717287) | 6100.59 (4704.38 to 7811.43) | 3807809 (2877984 to 4967594) | 6517.31 (4935.7 to 8469.33) | 0.01 (-0.13 to 0.16) |
| Palau | 176 (128 to 242) | 4407.96 (3204.87 to 6030.75) | 188 (134 to 267) | 4826.81 (3409.88 to 6899.34) | 0.08 (0.02 to 0.13) |
| Palestine | 38202 (26861 to 53465) | 9100.7 (6491.1 to 12584.52) | 131091 (87827 to 191836) | 10277.83 (6930.18 to 14929.98) | 0.02 (-0.15 to 0.19) |
| Panama | 25811 (18662 to 35338) | 4392.73 (3183.57 to 5981.72) | 57787 (39414 to 83845) | 5395.97 (3681.27 to 7822.94) | 0.24 (0.03 to 0.45) |
| Papua New Guinea | 42030 (31257 to 55845) | 4518.51 (3378.97 to 5973.14) | 116035 (84754 to 156441) | 4495.91 (3290.56 to 6042.97) | -0.14 (-0.17 to -0.11) |
| Paraguay | 49884 (36362 to 68059) | 5429.79 (3976.46 to 7357.19) | 137310 (94526 to 194880) | 7246.51 (4996.63 to 10266.51) | 0.39 (0.22 to 0.56) |
| Peru | 188440 (141224 to 249703) | 3623.59 (2726.33 to 4777.3) | 419427 (299626 to 578665) | 4326.45 (3086.66 to 5972.09) | -0.13 (-0.43 to 0.18) |
| Philippines | 627336 (493785 to 787061) | 4292.3 (3402.25 to 5336.24) | 1362928 (1071524 to 1717879) | 4717.28 (3719.95 to 5923.79) | -0.08 (-0.24 to 0.09) |
| Poland | 318480 (254139 to 395598) | 3309.44 (2637.74 to 4118.42) | 354240 (282134 to 441942) | 3768.22 (2979.24 to 4743.7) | -0.17 (-0.39 to 0.04) |
| Portugal | 233565 (170448 to 309603) | 9233.97 (6735.82 to 12244.32) | 241643 (167651 to 346050) | 10290.15 (7057.91 to 14864.56) | -0.42 (-0.71 to -0.13) |
| Puerto Rico | 37644 (28182 to 49487) | 3937.23 (2949.59 to 5173.6) | 35251 (24930 to 49396) | 4597.24 (3237.36 to 6479.5) | 0.12 (-0.07 to 0.3) |
| Qatar | 6060 (4430 to 8109) | 7654.96 (5588.14 to 10243.05) | 44504 (30826 to 63043) | 7725.64 (5302.87 to 11057.09) | -0.26 (-0.39 to -0.14) |
| Republic of Korea | 441218 (353351 to 552329) | 3461.74 (2772.86 to 4331.01) | 462215 (342720 to 611461) | 4026.54 (2964.06 to 5347.61) | 0.29 (0.18 to 0.41) |
| Republic of Moldova | 60047 (45663 to 77838) | 5299.15 (4023.06 to 6882.72) | 53003 (38202 to 73365) | 5658.67 (4028.6 to 7918.53) | -0.4 (-0.61 to -0.18) |
| Romania | 232101 (174840 to 302074) | 4122.32 (3108.37 to 5365.33) | 212222 (152802 to 289550) | 5007.59 (3576.2 to 6893.41) | 0.05 (-0.16 to 0.25) |
| Russian Federation | 1749450 (1374097 to 2202616) | 4627.58 (3629.68 to 5837.66) | 2031949 (1588368 to 2567896) | 5707.72 (4430.71 to 7299.01) | 0.08 (-0.15 to 0.31) |
| Rwanda | 128438 (94420 to 173053) | 8455.27 (6258.64 to 11311.39) | 286238 (201809 to 395335) | 8388.95 (5935.54 to 11523.39) | -0.63 (-0.79 to -0.47) |
| Saint Kitts and Nevis | 550 (383 to 794) | 5793.83 (4053.69 to 8305.12) | 1107 (723 to 1670) | 6928.16 (4511.5 to 10486.86) | 0.19 (0.06 to 0.33) |
| Saint Lucia | 1535 (1107 to 2116) | 4722.17 (3429.43 to 6454.14) | 2864 (1966 to 4068) | 6155.71 (4205 to 8787.37) | 0.26 (0.05 to 0.48) |
| Saint Vincent and the Grenadines | 1170 (842 to 1608) | 4702.28 (3403.75 to 6408.06) | 1647 (1140 to 2343) | 5864.13 (4051.94 to 8360.45) | 0.25 (0.07 to 0.43) |
| Samoa | 1399 (1045 to 1846) | 4029.77 (3031.11 to 5291.7) | 1970 (1428 to 2681) | 4162.41 (3033 to 5632.38) | -0.12 (-0.19 to -0.05) |
| San Marino | 446 (311 to 641) | 7139.52 (4988.51 to 10259.24) | 637 (420 to 954) | 8946.73 (5864.98 to 13464.18) | 0.28 (0.08 to 0.48) |
| Sao Tome and Principe | 1363 (1006 to 1817) | 5714.05 (4262.85 to 7553.91) | 3289 (2336 to 4556) | 6075.64 (4346.15 to 8380.46) | 0.08 (0 to 0.16) |
| Saudi Arabia | 212127 (150089 to 294392) | 6810.69 (4864.3 to 9359.5) | 792606 (555504 to 1117189) | 7648.16 (5349.08 to 10796.64) | 0.27 (0.14 to 0.4) |
| Senegal | 88034 (65719 to 117103) | 5486.23 (4128.3 to 7245.08) | 232408 (168007 to 320830) | 6228.02 (4523.47 to 8537.94) | 0.13 (-0.01 to 0.26) |
| Serbia | 111485 (85397 to 144646) | 4705.65 (3594.91 to 6122.06) | 106437 (78014 to 143788) | 5038.12 (3671.39 to 6845.51) | -0.33 (-0.5 to -0.15) |
| Seychelles | 625 (461 to 832) | 3674.58 (2725.73 to 4878) | 1065 (766 to 1465) | 4287.93 (3067.42 to 5926.56) | 0.06 (-0.09 to 0.21) |
| Sierra Leone | 58853 (43588 to 78297) | 6179.68 (4614.61 to 8149.14) | 141592 (100931 to 195743) | 6629.06 (4764.6 to 9098.54) | 0.27 (0.21 to 0.32) |
| Singapore | 49477 (40288 to 61240) | 5203.52 (4232.63 to 6438.46) | 50159 (36763 to 67821) | 3606.56 (2616.5 to 4926.4) | -1.85 (-2.16 to -1.54) |
| Slovakia | 59569 (45337 to 77217) | 4441.6 (3374.08 to 5771.1) | 70449 (50419 to 97251) | 5302.25 (3755.64 to 7385.86) | -0.1 (-0.34 to 0.14) |
| Slovenia | 29779 (22969 to 38283) | 5893.26 (4535.72 to 7593.75) | 25146 (18346 to 34465) | 5689.24 (4103.92 to 7863.84) | -0.71 (-0.9 to -0.51) |
| Solomon Islands | 3079 (2288 to 4107) | 4401.39 (3297.39 to 5818.95) | 7622 (5502 to 10396) | 4537.54 (3288.4 to 6163.01) | -0.13 (-0.19 to -0.07) |
| Somalia | 114703 (85490 to 151619) | 7080.69 (5296.96 to 9316.49) | 381749 (270900 to 534728) | 8315.82 (5955.29 to 11565.86) | 0.08 (-0.05 to 0.2) |
| South Africa | 615822 (494669 to 756492) | 6732.72 (5435.78 to 8220.37) | 1277528 (1014307 to 1591319) | 8172.99 (6482.16 to 10191.87) | 0.31 (0.09 to 0.53) |
| South Sudan | 82512 (61457 to 108973) | 6845.59 (5136.28 to 8961.27) | 160249 (114320 to 220935) | 7251.93 (5204.28 to 9963.97) | 0.06 (-0.01 to 0.13) |
| Spain | 687668 (580228 to 814429) | 7141.16 (6027.33 to 8454.34) | 1003410 (722984 to 1363599) | 10080.65 (7191.22 to 13906.82) | 1.18 (0.83 to 1.53) |
| Sri Lanka | 230435 (176539 to 294011) | 5086.27 (3904.56 to 6484.13) | 277400 (206039 to 369440) | 4880.87 (3620.89 to 6510.98) | -0.72 (-0.96 to -0.49) |
| Sudan | 324199 (234603 to 443349) | 7146.75 (5220.06 to 9665.18) | 849266 (588990 to 1215212) | 7666.66 (5352.25 to 10923.16) | -0.15 (-0.29 to -0.01) |
| Suriname | 6576 (4823 to 8855) | 6880.8 (5081.41 to 9217.2) | 14023 (9734 to 19738) | 9634.94 (6677.4 to 13576.8) | 0.35 (0.14 to 0.55) |
| Sweden | 173242 (138315 to 214236) | 8325.68 (6611.75 to 10327.75) | 215577 (164371 to 280630) | 9788.27 (7402.5 to 12801.83) | 0.1 (-0.06 to 0.25) |
| Switzerland | 140269 (108076 to 179296) | 7868.4 (6025.62 to 10087.87) | 155966 (111624 to 213742) | 7790.18 (5538.6 to 10719.25) | -0.67 (-1.04 to -0.29) |
| Syrian Arab Republic | 175256 (124543 to 246077) | 6707.95 (4813.56 to 9332.52) | 296112 (201546 to 423764) | 7698.29 (5258.78 to 11019.25) | 0.12 (-0.04 to 0.28) |
| Taiwan (Province of China) | 180045 (134984 to 237722) | 3367.15 (2521.06 to 4454.95) | 230937 (170809 to 306097) | 3733.87 (2745.59 to 4964.46) | 0.28 (0.26 to 0.31) |
| Tajikistan | 49721 (36893 to 66121) | 4396.39 (3297.78 to 5779.88) | 124852 (89396 to 171622) | 4963.11 (3555.74 to 6810.47) | -0.07 (-0.24 to 0.09) |
| Thailand | 645292 (486549 to 841057) | 4140.21 (3133.53 to 5398.56) | 748426 (547181 to 997639) | 4331.09 (3136.61 to 5814.41) | -0.17 (-0.25 to -0.1) |
| Timor-Leste | 7970 (5922 to 10582) | 4409.52 (3291.9 to 5828.59) | 14481 (10312 to 19828) | 4372.59 (3153.07 to 5934.71) | -0.43 (-0.56 to -0.29) |
| Togo | 50495 (37451 to 66804) | 6322.23 (4733.42 to 8291.83) | 138058 (98265 to 187938) | 6578.96 (4703.07 to 8915.26) | 0.01 (-0.05 to 0.07) |
| Tokelau | 15 (11 to 21) | 4407.47 (3205.68 to 6053.07) | 15 (11 to 22) | 4810.25 (3374.56 to 6783.44) | 0.07 (0.02 to 0.13) |
| Tonga | 835 (623 to 1095) | 3925.36 (2949.2 to 5133.14) | 1020 (746 to 1381) | 4107.9 (3016.77 to 5543.87) | -0.06 (-0.13 to 0.01) |
| Trinidad and Tobago | 18759 (13899 to 25049) | 6156.31 (4570.54 to 8206.78) | 26019 (17844 to 37358) | 7588.87 (5193.26 to 10928.96) | -0.11 (-0.35 to 0.13) |
| Tunisia | 157262 (111771 to 215636) | 7966.21 (5710.86 to 10839.64) | 315462 (213087 to 456466) | 10137.08 (6805.74 to 14762.34) | 0.13 (-0.1 to 0.36) |
| Turkey | 968521 (785906 to 1187681) | 6970.57 (5676.27 to 8509.34) | 1774244 (1210680 to 2559045) | 8134.31 (5530.83 to 11763.83) | 0.17 (0.03 to 0.32) |
| Turkmenistan | 38735 (29048 to 50777) | 4595.53 (3477.08 to 5991.68) | 63513 (45826 to 87603) | 5060.38 (3651.09 to 6976.25) | -0.07 (-0.21 to 0.07) |
| Tuvalu | 106 (78 to 146) | 4388.53 (3205.45 to 6035) | 136 (95 to 191) | 4779.75 (3357.58 to 6663.9) | 0.07 (0.02 to 0.13) |
| Uganda | 331351 (243090 to 444480) | 9321.19 (6889.08 to 12408.22) | 980319 (682031 to 1384021) | 9981.02 (7023.93 to 14031.7) | -0.43 (-0.68 to -0.18) |
| Ukraine | 779753 (600535 to 1007671) | 6006.83 (4615.79 to 7780.39) | 764474 (560534 to 1026821) | 7034.41 (5116.13 to 9520.52) | -0.36 (-0.57 to -0.16) |
| United Arab Emirates | 22847 (16859 to 30943) | 6837.54 (5048.56 to 9208.2) | 134552 (94342 to 188061) | 7275.73 (5046.09 to 10312.98) | -0.25 (-0.42 to -0.08) |
| United Kingdom | 1112440 (860491 to 1436649) | 7770.81 (6002.83 to 10046.36) | 1309064 (1004976 to 1689200) | 8440.8 (6445.34 to 10953.38) | -0.1 (-0.39 to 0.18) |
| United Republic of Tanzania | 381726 (284892 to 506869) | 6814.34 (5131.83 to 8989.1) | 1027560 (735757 to 1404619) | 7220.83 (5204.08 to 9811.39) | -0.09 (-0.18 to 0) |
| United States of America | 4777646 (3902270 to 5794757) | 7095.39 (5775.34 to 8638.87) | 8040512 (6533674 to 9806444) | 10709.46 (8679.46 to 13101.25) | 0.52 (0.28 to 0.75) |
| United States Virgin Islands | 1381 (1013 to 1867) | 4758.19 (3487.77 to 6458.62) | 1014 (715 to 1420) | 5768.45 (4052.16 to 8138.47) | 0.25 (0.09 to 0.41) |
| Uruguay | 37461 (27541 to 50499) | 4990.91 (3668.8 to 6729.79) | 58628 (40815 to 82098) | 7051.84 (4892.92 to 9902.71) | 0.78 (0.58 to 0.97) |
| Uzbekistan | 219042 (165335 to 286472) | 4709.36 (3575.42 to 6127.57) | 454720 (326072 to 619660) | 5055.67 (3614.66 to 6908.7) | -0.1 (-0.22 to 0.02) |
| Vanuatu | 1464 (1094 to 1943) | 4352.89 (3269.17 to 5749.01) | 3451 (2491 to 4720) | 4485.32 (3255.22 to 6103.53) | -0.14 (-0.21 to -0.07) |
| Venezuela (Bolivarian Republic of) | 214570 (156506 to 293115) | 4625.77 (3388.4 to 6277.05) | 356237 (246214 to 505997) | 5081.86 (3503.33 to 7236.73) | 0.05 (-0.04 to 0.15) |
| Viet Nam | 638709 (479693 to 839330) | 3966.03 (2997.7 to 5212.13) | 1086527 (798291 to 1445648) | 4154.23 (3032.14 to 5565.92) | -0.23 (-0.33 to -0.13) |
| Yemen | 224325 (161457 to 306310) | 8471.75 (6141.85 to 11477.68) | 684565 (466384 to 974319) | 8425.26 (5765.38 to 11941.14) | -0.12 (-0.17 to -0.06) |
| Zambia | 100907 (75518 to 133431) | 5958.26 (4503.02 to 7831.82) | 307913 (221585 to 419556) | 6563.27 (4744.54 to 8880.47) | -0.07 (-0.18 to 0.03) |
| Zimbabwe | 103192 (75966 to 136410) | 4675.85 (3472.13 to 6133.84) | 207357 (149301 to 283382) | 5310.4 (3841.37 to 7203.78) | 0.11 (0 to 0.23) |

**Supplementary Table S6** The DALYs of depressive disorders cases and rates among WCBA in 204 countries and territories in 1990 and 2021, and the trends from 1990 to 2021.

| Location | DALYs | | | | |
| --- | --- | --- | --- | --- | --- |
|  | No.,1990 (95% UI) | ASDALYR, 1990 per 100,000 (95% UI) | No.,2021, (95% UI) | ASDALYR, 2021 per 100,000 (95% UI) | EAPC, 1990-2021, (95% CI) |
| Afghanistan | 30636 (18080 to 47831) | 1467.19 (872 to 2288.33) | 120270 (69142 to 190420) | 1738.49 (1006.75 to 2735.55) | 0.13 (-0.01 to 0.28) |
| Albania | 5151 (3141 to 7834) | 629.52 (383.85 to 953.55) | 5320 (3167 to 8514) | 857.6 (508.91 to 1375.79) | 0.34 (0.13 to 0.56) |
| Algeria | 69753 (40599 to 109697) | 1251.56 (733.49 to 1957.49) | 154768 (86206 to 251372) | 1362.78 (756.91 to 2220.47) | 0.04 (-0.12 to 0.19) |
| American Samoa | 66 (40 to 100) | 563.6 (342.18 to 846.96) | 72 (43 to 111) | 615.2 (365.8 to 948.5) | 0.02 (-0.07 to 0.11) |
| Andorra | 177 (106 to 270) | 1177.68 (703.29 to 1798.15) | 286 (163 to 454) | 1437.56 (815.97 to 2304.14) | 0.14 (-0.11 to 0.39) |
| Angola | 37706 (22532 to 58808) | 1699.88 (1018.64 to 2642.69) | 138349 (77423 to 222285) | 1864.14 (1045.86 to 2993.97) | 0 (-0.12 to 0.11) |
| Antigua and Barbuda | 130 (75 to 203) | 811.61 (472.89 to 1268.19) | 251 (143 to 415) | 1021.56 (578.3 to 1691.03) | 0.24 (0 to 0.48) |
| Argentina | 67901 (41332 to 103875) | 846.26 (515.48 to 1294.36) | 120725 (72759 to 181282) | 1024.7 (616.35 to 1540.41) | -0.06 (-0.31 to 0.19) |
| Armenia | 5596 (3364 to 8546) | 657.37 (395.32 to 1000.41) | 6631 (3831 to 10580) | 883.79 (507.39 to 1416.46) | 0.37 (0.12 to 0.62) |
| Australia | 63364 (40878 to 92083) | 1422.72 (917.32 to 2070.04) | 95816 (57740 to 147046) | 1616.63 (973.34 to 2487.3) | 0.24 (0.07 to 0.42) |
| Austria | 22345 (13801 to 33209) | 1121.09 (690.69 to 1667.13) | 22376 (13178 to 34832) | 1134.93 (662.58 to 1779.43) | -0.59 (-0.86 to -0.32) |
| Azerbaijan | 11855 (7067 to 18228) | 654.65 (391.23 to 1001.4) | 23482 (13374 to 37485) | 843.98 (478.28 to 1354.82) | 0.27 (0.03 to 0.51) |
| Bahamas | 579 (338 to 894) | 817.06 (478.2 to 1258.82) | 1147 (647 to 1862) | 1057.73 (596.04 to 1720.37) | 0.29 (0.04 to 0.55) |
| Bahrain | 1752 (1062 to 2705) | 1539.52 (934.76 to 2361.57) | 5376 (3113 to 8549) | 1639.21 (947.15 to 2608.56) | -0.4 (-0.59 to -0.22) |
| Bangladesh | 323985 (195562 to 490277) | 1391.32 (839.25 to 2093.55) | 697249 (395144 to 1109374) | 1532.24 (868.79 to 2434.64) | -0.04 (-0.18 to 0.11) |
| Barbados | 553 (325 to 865) | 812.42 (476.49 to 1270.26) | 781 (439 to 1267) | 1081.02 (605.98 to 1761.89) | 0.33 (0.1 to 0.55) |
| Belarus | 24945 (15276 to 37597) | 974.97 (595.94 to 1469.08) | 31919 (18765 to 49247) | 1431.07 (835.54 to 2214.49) | 0.23 (-0.03 to 0.49) |
| Belgium | 24700 (16754 to 34354) | 1009.85 (684.18 to 1407.79) | 33687 (19583 to 51837) | 1348.66 (779.7 to 2084.89) | 0.78 (0.58 to 0.98) |
| Belize | 331 (196 to 520) | 828.47 (492.22 to 1295.77) | 1248 (716 to 1980) | 1044.23 (600.22 to 1655.07) | 0.36 (0.17 to 0.55) |
| Benin | 10839 (6499 to 16459) | 1045.08 (633.76 to 1571.76) | 35108 (20557 to 56189) | 1142.83 (675.38 to 1814.83) | 0.1 (0.04 to 0.17) |
| Bermuda | 171 (103 to 263) | 983.4 (589.81 to 1515.3) | 148 (83 to 240) | 1074.18 (595.5 to 1751.46) | -0.39 (-0.64 to -0.15) |
| Bhutan | 1342 (804 to 2051) | 1050.61 (631.11 to 1595.83) | 1999 (1150 to 3215) | 979.32 (563.36 to 1572.56) | -0.3 (-0.41 to -0.18) |
| Bolivia (Plurinational State of) | 14969 (8958 to 23271) | 1019.53 (612.55 to 1579.54) | 40939 (23242 to 65163) | 1318.08 (748.8 to 2095.14) | 0.03 (-0.32 to 0.38) |
| Bosnia and Herzegovina | 10406 (6340 to 15660) | 895.74 (545.55 to 1346.58) | 7072 (4063 to 11380) | 940.57 (538.15 to 1517.89) | -0.77 (-1.04 to -0.5) |
| Botswana | 3108 (1890 to 4725) | 1039.27 (635.89 to 1567.97) | 9075 (5256 to 13903) | 1333.69 (773.21 to 2042.27) | 0.13 (-0.03 to 0.29) |
| Brazil | 468480 (303647 to 683448) | 1231.4 (799.38 to 1789.95) | 851092 (538828 to 1238806) | 1420.96 (899.62 to 2073.59) | -0.42 (-0.77 to -0.07) |
| Brunei Darussalam | 324 (194 to 506) | 470.92 (281.98 to 733.24) | 686 (385 to 1104) | 550.2 (307.92 to 886.51) | 0.16 (-0.04 to 0.36) |
| Bulgaria | 15966 (9877 to 23798) | 758.91 (467.95 to 1135.85) | 14226 (8318 to 22069) | 969.02 (564.4 to 1524.96) | -0.34 (-0.66 to -0.02) |
| Burkina Faso | 20151 (12258 to 30519) | 1013.42 (619.8 to 1525.61) | 53990 (31543 to 85258) | 1039.72 (612.19 to 1631.59) | 0.36 (0.24 to 0.48) |
| Burundi | 15949 (9688 to 24172) | 1339.96 (814.25 to 2019.6) | 38413 (21917 to 61129) | 1287.92 (741.1 to 2039.59) | -0.66 (-0.83 to -0.49) |
| Cabo Verde | 754 (445 to 1158) | 1028.45 (612.48 to 1568.92) | 1989 (1153 to 3221) | 1336.91 (775.81 to 2161.69) | 0.39 (0.23 to 0.55) |
| Cambodia | 18754 (11376 to 28729) | 763.07 (464.57 to 1162.5) | 34859 (20952 to 54454) | 776.41 (466.92 to 1211.89) | -0.64 (-0.82 to -0.47) |
| Cameroon | 24533 (14819 to 37752) | 1095.17 (667.7 to 1674) | 88669 (51718 to 139159) | 1190.77 (698.59 to 1857.28) | 0.08 (0.02 to 0.14) |
| Canada | 78535 (51018 to 113879) | 1065.83 (691.49 to 1550.91) | 116418 (70007 to 179903) | 1428.52 (854.37 to 2219.08) | 0.08 (-0.19 to 0.36) |
| Central African Republic | 10382 (6253 to 16163) | 1674.02 (1007.82 to 2593.02) | 23726 (13884 to 37363) | 1769.99 (1038.09 to 2776.21) | 0.06 (-0.04 to 0.16) |
| Chad | 15995 (9571 to 24566) | 1272.41 (766.24 to 1940.55) | 48029 (27979 to 77029) | 1337.68 (782.91 to 2132.62) | 0.1 (0.02 to 0.17) |
| Chile | 57348 (38019 to 82074) | 1572.71 (1043.02 to 2249.21) | 79172 (44975 to 122927) | 1701.85 (965.04 to 2644.47) | -0.39 (-0.64 to -0.13) |
| China | 2428799 (1584422 to 3523074) | 773.43 (505.65 to 1117.2) | 1934533 (1273866 to 2742775) | 559.4 (365.96 to 798.15) | -1.18 (-1.38 to -0.98) |
| Colombia | 49360 (29680 to 76475) | 582.78 (350.21 to 901.87) | 72147 (41757 to 113507) | 547.77 (316.78 to 861.78) | -0.94 (-1.3 to -0.57) |
| Comoros | 1004 (611 to 1526) | 1011.33 (619.12 to 1526.9) | 2171 (1240 to 3495) | 1129.22 (646.41 to 1814.21) | -0.03 (-0.16 to 0.11) |
| Congo | 9002 (5430 to 13971) | 1681.48 (1018.17 to 2598.25) | 25460 (14575 to 40909) | 1805.21 (1035.47 to 2895.4) | -0.12 (-0.29 to 0.06) |
| Cook Islands | 32 (19 to 51) | 709.18 (424.01 to 1121.37) | 34 (20 to 56) | 793.46 (455.68 to 1295.49) | 0.09 (0.02 to 0.16) |
| Costa Rica | 6212 (3705 to 9699) | 823.22 (491.17 to 1284.25) | 14247 (8096 to 23126) | 1084.8 (615.86 to 1761.8) | 0.44 (0.26 to 0.61) |
| Coted'Ivoire | 23658 (14346 to 35967) | 921.39 (561.26 to 1388.74) | 61786 (36196 to 95651) | 964.1 (567.59 to 1484.87) | 0.04 (-0.04 to 0.12) |
| Croatia | 10737 (6600 to 15924) | 878.31 (539.66 to 1306.08) | 8151 (4899 to 12647) | 876.05 (522.28 to 1367.84) | -0.71 (-0.95 to -0.46) |
| Cuba | 46863 (29226 to 71004) | 1534.42 (957.34 to 2325.6) | 30798 (18492 to 49234) | 1199.67 (714.01 to 1926.6) | -1.75 (-2.09 to -1.4) |
| Cyprus | 1962 (1152 to 3042) | 991.89 (581.75 to 1537.79) | 4174 (2349 to 6624) | 1180.53 (661.48 to 1886.54) | 0.17 (-0.03 to 0.37) |
| Czechia | 22578 (13859 to 33332) | 859.96 (527.06 to 1273.36) | 22038 (12850 to 34056) | 915.75 (530.33 to 1426.95) | -0.58 (-0.82 to -0.33) |
| Democratic People's Republic of Korea | 35445 (21874 to 53337) | 635.08 (391.56 to 954.41) | 40627 (24706 to 62119) | 598.54 (362.73 to 917.84) | -0.41 (-0.48 to -0.34) |
| Democratic Republic of the Congo | 128256 (78714 to 197574) | 1558.58 (960.4 to 2387.84) | 333560 (193637 to 522281) | 1619 (943.67 to 2520.76) | 0.02 (-0.08 to 0.12) |
| Denmark | 19489 (12092 to 28966) | 1463.71 (906.25 to 2179.38) | 16664 (9746 to 25899) | 1309.14 (762.47 to 2039.56) | -0.94 (-1.23 to -0.65) |
| Djibouti | 983 (580 to 1513) | 1059.39 (629.87 to 1622.07) | 3844 (2192 to 6041) | 1196.74 (682.66 to 1878.92) | 0.12 (0.01 to 0.24) |
| Dominica | 133 (78 to 208) | 812.35 (477.43 to 1269.04) | 171 (95 to 283) | 1039.42 (579.63 to 1722.24) | 0.25 (0.02 to 0.49) |
| Dominican Republic | 19088 (11179 to 30002) | 1055.59 (618.97 to 1652.7) | 35652 (19590 to 58637) | 1232.75 (677.03 to 2026.13) | 0.15 (-0.04 to 0.33) |
| Ecuador | 22431 (13372 to 34546) | 902.99 (537.95 to 1388.48) | 55490 (30914 to 88215) | 1173.73 (653.94 to 1865.42) | 0.24 (-0.04 to 0.53) |
| Egypt | 144321 (85319 to 226513) | 1125 (667.63 to 1763.04) | 353478 (199589 to 569430) | 1369.8 (774.82 to 2203.67) | 0.26 (0.06 to 0.45) |
| El Salvador | 15008 (9172 to 23405) | 1164.36 (709.18 to 1813.91) | 21551 (12340 to 34927) | 1208.36 (691.37 to 1956.69) | -0.39 (-0.61 to -0.17) |
| Equatorial Guinea | 1619 (969 to 2506) | 1698.5 (1018.79 to 2617.19) | 6412 (3733 to 10364) | 1805.21 (1052.66 to 2908.53) | 0.06 (-0.05 to 0.17) |
| Eritrea | 8760 (5280 to 13278) | 1171.87 (710.22 to 1765.77) | 20021 (11267 to 31160) | 1246.45 (704.25 to 1933.11) | -0.04 (-0.14 to 0.05) |
| Estonia | 4822 (2974 to 7245) | 1239.55 (764.46 to 1867.28) | 3459 (2029 to 5442) | 1191.72 (697.84 to 1885.33) | -1.19 (-1.46 to -0.92) |
| Eswatini | 1877 (1127 to 2840) | 1033.43 (627.29 to 1554.84) | 4791 (2701 to 7666) | 1553.33 (875.69 to 2481.72) | 0.5 (0.26 to 0.75) |
| Ethiopia | 121019 (77583 to 177722) | 1137.4 (728.61 to 1664.3) | 302261 (192306 to 452507) | 1148.5 (732.79 to 1712.3) | -0.34 (-0.49 to -0.18) |
| Fiji | 1236 (763 to 1879) | 641.77 (396.82 to 971.49) | 1715 (1026 to 2719) | 750.91 (449.61 to 1191.12) | 0.02 (-0.11 to 0.14) |
| Finland | 20507 (12777 to 30482) | 1606.86 (1001.22 to 2396.11) | 19215 (11546 to 29476) | 1708.73 (1024.58 to 2629.22) | -0.48 (-0.7 to -0.25) |
| France | 211808 (141725 to 291544) | 1459.28 (976.07 to 2010.51) | 219867 (130382 to 342895) | 1531.95 (905.21 to 2395.1) | -0.35 (-0.56 to -0.13) |
| Gabon | 3249 (1903 to 5002) | 1533.72 (904.24 to 2347.78) | 8176 (4670 to 13059) | 1704.85 (977.61 to 2716.02) | -0.07 (-0.2 to 0.06) |
| Gambia | 3708 (2219 to 5786) | 1762.72 (1063.05 to 2718.53) | 10691 (5956 to 17409) | 1841.43 (1031.13 to 2981.19) | -0.45 (-0.62 to -0.28) |
| Georgia | 9962 (6050 to 15275) | 726.46 (440.78 to 1112.51) | 7585 (4340 to 11886) | 936.64 (532.88 to 1474.95) | 0.2 (-0.02 to 0.41) |
| Germany | 176486 (116330 to 251208) | 902.45 (592.66 to 1288.35) | 216611 (127783 to 337967) | 1274.94 (747.14 to 1997.45) | 1.05 (0.89 to 1.22) |
| Ghana | 35147 (20979 to 54421) | 1049.66 (628.56 to 1620.04) | 101595 (59619 to 166232) | 1139.9 (670.8 to 1859.62) | -0.01 (-0.1 to 0.09) |
| Greece | 41026 (23960 to 64480) | 1623.44 (947.09 to 2553.56) | 44584 (25033 to 73163) | 2062.84 (1154.22 to 3385.78) | -0.01 (-0.38 to 0.36) |
| Greenland | 404 (254 to 610) | 2649.15 (1661.73 to 3999.62) | 384 (230 to 602) | 3015.73 (1803.63 to 4729.04) | -0.32 (-0.54 to -0.1) |
| Grenada | 155 (92 to 242) | 828.37 (490 to 1288.19) | 267 (149 to 435) | 1037.84 (578.58 to 1689.75) | 0.24 (0.05 to 0.42) |
| Guam | 230 (142 to 346) | 663.24 (410.03 to 996.9) | 285 (170 to 445) | 785.89 (467.25 to 1229.99) | 0.17 (0.01 to 0.33) |
| Guatemala | 17494 (10184 to 27556) | 1021.94 (599.12 to 1598.76) | 54107 (30429 to 89845) | 1258.66 (708.61 to 2081.29) | 0.06 (-0.17 to 0.29) |
| Guinea | 14143 (8467 to 21518) | 1074.62 (644.84 to 1631.12) | 38017 (22111 to 60547) | 1203.7 (703.87 to 1906.31) | 0.13 (0.03 to 0.22) |
| Guinea-Bissau | 2424 (1438 to 3747) | 1106.37 (658.64 to 1703.15) | 6160 (3562 to 9771) | 1228.17 (714.31 to 1941.24) | 0.15 (0.07 to 0.24) |
| Guyana | 2603 (1577 to 3976) | 1298.86 (788.41 to 1973.55) | 3870 (2263 to 6228) | 1895.95 (1108.9 to 3047.55) | 0.6 (0.42 to 0.79) |
| Haiti | 15636 (9289 to 24394) | 1041.64 (616.97 to 1618.25) | 42184 (23888 to 66699) | 1198.04 (679.13 to 1892.76) | -0.01 (-0.17 to 0.15) |
| Honduras | 7904 (4647 to 12399) | 800.06 (471.89 to 1247.01) | 29902 (16445 to 48731) | 1077.42 (594.07 to 1748.12) | 0.36 (0.13 to 0.59) |
| Hungary | 23351 (14603 to 34712) | 894.94 (558.36 to 1335.05) | 18837 (11074 to 29525) | 827.54 (481.44 to 1304.46) | -0.99 (-1.22 to -0.76) |
| Iceland | 677 (415 to 1021) | 1041.24 (639.11 to 1568.62) | 808 (466 to 1265) | 1015.97 (584.09 to 1595.77) | -0.42 (-0.6 to -0.25) |
| India | 2041894 (1308570 to 3009491) | 1054.95 (676.64 to 1547.89) | 3959613 (2541582 to 5762161) | 1055.26 (677.78 to 1533.98) | -1.08 (-1.41 to -0.75) |
| Indonesia | 245895 (158599 to 357383) | 532.25 (344.5 to 770.85) | 500179 (322304 to 732808) | 658.51 (423.78 to 966.01) | 0.19 (-0.01 to 0.38) |
| Iran (Islamic Republic of) | 194455 (120358 to 292990) | 1577.37 (978.55 to 2367.72) | 430877 (268064 to 647016) | 1834.46 (1140.72 to 2763.83) | 0.56 (0.36 to 0.76) |
| Iraq | 46849 (28410 to 71655) | 1205.45 (737.55 to 1828.13) | 134953 (79149 to 215094) | 1299.1 (763.76 to 2065.02) | 0.18 (-0.27 to 0.63) |
| Ireland | 10919 (6942 to 16014) | 1241.29 (789.85 to 1817.8) | 19870 (11695 to 31148) | 1732.99 (1018.68 to 2725.72) | 0.44 (0.2 to 0.68) |
| Israel | 16543 (9990 to 25077) | 1359.92 (822.57 to 2058.08) | 34037 (19322 to 55410) | 1531.79 (869.04 to 2497.5) | -0.33 (-0.54 to -0.13) |
| Italy | 170632 (107771 to 252948) | 1190.02 (751.5 to 1764.83) | 182076 (114082 to 271110) | 1504.31 (939.71 to 2244.91) | -0.23 (-0.55 to 0.09) |
| Jamaica | 4709 (2735 to 7444) | 824.1 (479.6 to 1295.03) | 8268 (4711 to 13736) | 1066.16 (607.18 to 1771.42) | 0.24 (0 to 0.49) |
| Japan | 194256 (127043 to 279452) | 612.69 (400.09 to 882.52) | 198475 (129720 to 286168) | 823.3 (534.89 to 1193.15) | 0.52 (0.26 to 0.78) |
| Jordan | 11180 (6591 to 17505) | 1422.61 (847.59 to 2214.94) | 44560 (25188 to 72624) | 1453.17 (823.97 to 2363.84) | -0.35 (-0.5 to -0.21) |
| Kazakhstan | 32676 (20366 to 48695) | 806.73 (503.8 to 1201.82) | 47116 (28763 to 73486) | 986.53 (600.61 to 1546.09) | 0.43 (0.27 to 0.59) |
| Kenya | 55830 (35857 to 81289) | 1176.36 (756.14 to 1700.31) | 152288 (97413 to 222695) | 1201.6 (768.1 to 1750.34) | -0.27 (-0.4 to -0.13) |
| Kiribati | 122 (76 to 185) | 660.78 (409.32 to 997.41) | 210 (127 to 325) | 662.21 (401.28 to 1019.98) | -0.25 (-0.35 to -0.15) |
| Kuwait | 5114 (3039 to 8039) | 1249.42 (743.55 to 1963.29) | 18728 (10761 to 30674) | 1238.26 (707.21 to 2039.42) | -0.15 (-0.25 to -0.05) |
| Kyrgyzstan | 8455 (5229 to 12746) | 842.06 (522.24 to 1261.41) | 17331 (10054 to 27436) | 1007.32 (584.26 to 1592.94) | 0.05 (-0.15 to 0.25) |
| Lao People's Democratic Republic | 5893 (3661 to 8939) | 622.23 (386.6 to 939.84) | 12061 (7167 to 18871) | 611.16 (364.08 to 954.39) | -0.44 (-0.67 to -0.21) |
| Latvia | 7094 (4322 to 10572) | 1061.16 (645.2 to 1586.76) | 5053 (2935 to 7946) | 1226.57 (706.52 to 1942.91) | -0.4 (-0.64 to -0.16) |
| Lebanon | 9092 (5576 to 13899) | 1230.55 (755.33 to 1879.96) | 28652 (16003 to 46338) | 1912.82 (1065.68 to 3096.8) | 0.45 (0.21 to 0.7) |
| Lesotho | 5887 (3544 to 9153) | 1609.8 (971.82 to 2494.39) | 10022 (5845 to 15374) | 2030.26 (1190.98 to 3097.24) | 0.31 (0.07 to 0.55) |
| Liberia | 6874 (4113 to 10536) | 1315.2 (793.79 to 2000.77) | 18439 (10674 to 30028) | 1374.76 (799.69 to 2231.4) | 0.46 (0.2 to 0.71) |
| Libya | 11163 (6536 to 17419) | 1286.4 (761.52 to 1992.69) | 28986 (16452 to 46545) | 1441.79 (817.77 to 2316.73) | 0.1 (-0.04 to 0.23) |
| Lithuania | 10262 (6299 to 15309) | 1097.91 (673.43 to 1640.1) | 8591 (5045 to 13425) | 1433.12 (837.56 to 2256.8) | -0.21 (-0.48 to 0.07) |
| Luxembourg | 1224 (751 to 1826) | 1246.01 (762.66 to 1860.18) | 1815 (1111 to 2733) | 1168.23 (707.73 to 1772.61) | -0.77 (-1.01 to -0.52) |
| Madagascar | 28842 (17359 to 44283) | 1137.18 (687.78 to 1732.3) | 87434 (49239 to 139162) | 1256 (710.34 to 1984.8) | -0.01 (-0.14 to 0.12) |
| Malawi | 21505 (13189 to 32597) | 1010.89 (623.6 to 1520.44) | 52635 (30967 to 82078) | 1101.29 (650.84 to 1702.3) | -0.21 (-0.34 to -0.07) |
| Malaysia | 30046 (17961 to 46142) | 691.13 (414.66 to 1057.23) | 68434 (39734 to 107820) | 816.09 (473.13 to 1285.97) | 0.89 (0.66 to 1.12) |
| Maldives | 450 (275 to 676) | 970.42 (594.3 to 1448.5) | 961 (575 to 1493) | 831.27 (496.82 to 1295.22) | -1.1 (-1.29 to -0.92) |
| Mali | 16243 (9836 to 24666) | 887.5 (539.34 to 1342.8) | 46497 (27335 to 73354) | 908.43 (536.91 to 1423.78) | -0.08 (-0.21 to 0.04) |
| Malta | 941 (560 to 1454) | 991.75 (589.19 to 1538.78) | 1081 (623 to 1725) | 1156.06 (663.49 to 1851.95) | 0.19 (-0.03 to 0.4) |
| Marshall Islands | 60 (37 to 92) | 632.55 (391.48 to 966.19) | 99 (60 to 155) | 673.24 (405.18 to 1050.55) | -0.11 (-0.19 to -0.03) |
| Mauritania | 3704 (2272 to 5604) | 834.26 (515.6 to 1253.9) | 8994 (5155 to 14340) | 878.72 (507.13 to 1389.44) | -0.07 (-0.2 to 0.05) |
| Mauritius | 3621 (2210 to 5437) | 1215.71 (741.38 to 1821.32) | 4107 (2367 to 6607) | 1294.63 (744.65 to 2084.36) | -0.49 (-0.69 to -0.29) |
| Mexico | 169311 (107851 to 248369) | 820.2 (523.33 to 1199.61) | 513075 (323387 to 760983) | 1454.88 (916.9 to 2159.53) | 1.69 (1.41 to 1.96) |
| Micronesia (Federated States of) | 147 (90 to 222) | 650.15 (400.23 to 979.88) | 174 (101 to 270) | 670.81 (390.51 to 1038.46) | -0.17 (-0.25 to -0.08) |
| Monaco | 92 (53 to 148) | 1293.02 (751.98 to 2093.35) | 117 (64 to 195) | 1639.59 (897.03 to 2738.86) | 0.23 (0.05 to 0.4) |
| Mongolia | 5128 (3107 to 7855) | 1043.44 (633.03 to 1586.33) | 8928 (5325 to 14028) | 1039.75 (618.61 to 1636.19) | -0.25 (-0.3 to -0.2) |
| Montenegro | 1164 (713 to 1740) | 747.27 (458.22 to 1117.09) | 1439 (853 to 2245) | 978.59 (576.16 to 1530.96) | 0.09 (-0.14 to 0.33) |
| Morocco | 92224 (55072 to 142143) | 1511.08 (907.1 to 2320.25) | 169166 (97719 to 267986) | 1739.34 (1003.43 to 2759.08) | 0.01 (-0.19 to 0.22) |
| Mozambique | 33183 (20006 to 51083) | 1097.73 (665.55 to 1680.78) | 93503 (55101 to 149775) | 1293.72 (768.37 to 2060.99) | 0.14 (0.02 to 0.25) |
| Myanmar | 41354 (24890 to 63550) | 416.13 (251.59 to 638.2) | 79427 (46890 to 124308) | 525.32 (310.25 to 821.76) | 0.2 (-0.07 to 0.48) |
| Namibia | 2784 (1685 to 4218) | 877.49 (533.86 to 1318.29) | 7539 (4363 to 12070) | 1157.85 (669.71 to 1851.8) | 0 (-0.29 to 0.3) |
| Nauru | 17 (10 to 26) | 701.29 (422.39 to 1095.26) | 22 (12 to 37) | 788.37 (443.93 to 1305.02) | 0.11 (0.03 to 0.18) |
| Nepal | 47147 (28799 to 71899) | 1098.82 (672.85 to 1673.78) | 135811 (80015 to 216048) | 1534.59 (906.14 to 2434.23) | 0.61 (0.38 to 0.85) |
| Netherlands | 47178 (31387 to 65959) | 1179.2 (784.28 to 1650.97) | 51453 (29781 to 81630) | 1398.57 (807.42 to 2229.3) | -0.08 (-0.33 to 0.18) |
| New Zealand | 10578 (6586 to 15982) | 1169.55 (728.07 to 1767.46) | 15085 (9429 to 22974) | 1290.62 (805.58 to 1966.62) | 0.17 (0.13 to 0.22) |
| Nicaragua | 7904 (4702 to 12277) | 909.41 (539.7 to 1404.09) | 20161 (11839 to 32590) | 1113.63 (653.62 to 1800.36) | 0.09 (-0.11 to 0.29) |
| Niger | 17292 (10457 to 26572) | 1058.33 (645.02 to 1611.95) | 51470 (30078 to 81355) | 1051.01 (625.44 to 1651.39) | -0.03 (-0.07 to 0.02) |
| Nigeria | 185471 (117900 to 274197) | 997.89 (634.6 to 1471.88) | 470963 (299887 to 694351) | 871.35 (555.81 to 1280.77) | -0.66 (-0.82 to -0.5) |
| Niue | 3 (2 to 5) | 706.18 (422 to 1107.19) | 3 (2 to 5) | 788.15 (455.45 to 1262.85) | 0.08 (0.01 to 0.16) |
| North Macedonia | 3514 (2143 to 5311) | 690.13 (421.05 to 1042.75) | 4934 (2873 to 7617) | 911.98 (528.85 to 1417.11) | -0.01 (-0.32 to 0.31) |
| Northern Mariana Islands | 74 (44 to 113) | 543.14 (328.16 to 834.07) | 78 (47 to 122) | 674.72 (401.05 to 1060.01) | 0.24 (0.08 to 0.4) |
| Norway | 11915 (7597 to 17578) | 1125.07 (717.53 to 1660.69) | 16887 (10584 to 25093) | 1393 (871.14 to 2074.45) | 0.27 (0.11 to 0.44) |
| Oman | 3930 (2308 to 6161) | 1193.29 (701.01 to 1866.32) | 14560 (8143 to 23623) | 1421.74 (791.39 to 2311.35) | 0.19 (0.01 to 0.38) |
| Pakistan | 231455 (145756 to 347798) | 1057.58 (665.51 to 1584.7) | 668287 (410119 to 1017822) | 1142.53 (701.64 to 1737.27) | 0.02 (-0.15 to 0.19) |
| Palau | 29 (17 to 45) | 705.29 (419.82 to 1112.42) | 30 (17 to 50) | 791.69 (454.18 to 1308.2) | 0.09 (0.02 to 0.17) |
| Palestine | 7121 (4110 to 11216) | 1681.85 (978.89 to 2632.86) | 24665 (13798 to 40447) | 1923.44 (1079.19 to 3141.52) | 0.03 (-0.15 to 0.21) |
| Panama | 4618 (2748 to 7259) | 782.89 (465.81 to 1229.65) | 10593 (5964 to 17450) | 989.08 (556.7 to 1628.88) | 0.27 (0.03 to 0.52) |
| Papua New Guinea | 6875 (4222 to 10595) | 722.75 (443.96 to 1107.48) | 18753 (10842 to 29251) | 720.32 (417.67 to 1122.6) | -0.17 (-0.21 to -0.13) |
| Paraguay | 8998 (5360 to 13945) | 972.39 (579.48 to 1503.74) | 25509 (14620 to 40748) | 1343.83 (769.79 to 2143.43) | 0.44 (0.25 to 0.63) |
| Peru | 31725 (18974 to 48573) | 605.36 (363.55 to 924) | 72861 (42877 to 117311) | 752.59 (442.61 to 1212.13) | -0.16 (-0.54 to 0.22) |
| Philippines | 100986 (64462 to 148374) | 679.43 (435.85 to 993.71) | 223734 (142652 to 329556) | 769.43 (491.31 to 1131.03) | -0.1 (-0.32 to 0.12) |
| Poland | 47858 (30775 to 69566) | 499.46 (321.65 to 726.06) | 55420 (35494 to 81282) | 597.77 (382.01 to 880.95) | -0.23 (-0.52 to 0.07) |
| Portugal | 43769 (26908 to 66399) | 1730.88 (1063.47 to 2626.8) | 45480 (25764 to 71290) | 1946.39 (1095.03 to 3084.17) | -0.46 (-0.78 to -0.14) |
| Puerto Rico | 6432 (3869 to 9845) | 672.53 (404.73 to 1029.11) | 6170 (3462 to 9673) | 808.74 (451.79 to 1271.11) | 0.14 (-0.08 to 0.36) |
| Qatar | 1097 (653 to 1702) | 1383.85 (822.49 to 2144.33) | 8000 (4635 to 12964) | 1399.2 (802.71 to 2286.54) | -0.3 (-0.44 to -0.16) |
| Republic of Korea | 76944 (49003 to 111437) | 601.41 (383.34 to 870.88) | 81842 (50392 to 124532) | 720.17 (440.65 to 1098.91) | 0.36 (0.23 to 0.49) |
| Republic of Moldova | 10360 (6374 to 15579) | 916.18 (562.71 to 1379.99) | 9213 (5378 to 14575) | 992.76 (573.7 to 1587.68) | -0.48 (-0.74 to -0.22) |
| Romania | 37744 (22759 to 56774) | 670.54 (404.87 to 1007.41) | 35930 (21500 to 56089) | 856.96 (508.46 to 1351.34) | 0.07 (-0.19 to 0.32) |
| Russian Federation | 291520 (184791 to 429054) | 773.08 (490.02 to 1137.32) | 350940 (221013 to 519370) | 997.99 (627.67 to 1482.82) | 0.1 (-0.18 to 0.39) |
| Rwanda | 22628 (13542 to 35078) | 1487.51 (894.63 to 2289.63) | 50553 (28876 to 81349) | 1476.32 (846.76 to 2363.8) | -0.73 (-0.92 to -0.54) |
| Saint Kitts and Nevis | 100 (58 to 163) | 1050.86 (609 to 1696.13) | 206 (114 to 344) | 1291.13 (711.21 to 2163.94) | 0.24 (0.09 to 0.39) |
| Saint Lucia | 272 (161 to 422) | 829.52 (492.42 to 1279.8) | 521 (295 to 851) | 1126.35 (635.25 to 1846.24) | 0.31 (0.06 to 0.56) |
| Saint Vincent and the Grenadines | 208 (122 to 323) | 828.44 (485.17 to 1278.63) | 299 (168 to 483) | 1066.26 (601.21 to 1723.3) | 0.29 (0.08 to 0.5) |
| Samoa | 225 (138 to 339) | 626.76 (387.9 to 945.57) | 314 (183 to 496) | 653.55 (382.13 to 1025.33) | -0.18 (-0.28 to -0.08) |
| San Marino | 81 (47 to 131) | 1301.22 (750.49 to 2101.54) | 118 (65 to 199) | 1672.22 (907.89 to 2805.44) | 0.3 (0.08 to 0.53) |
| Sao Tome and Principe | 229 (135 to 347) | 950.92 (569.11 to 1440.35) | 559 (326 to 896) | 1027.46 (602.74 to 1647.28) | 0.11 (0.01 to 0.21) |
| Saudi Arabia | 37891 (22224 to 59601) | 1205.81 (714.05 to 1890.17) | 142574 (84042 to 226506) | 1383.82 (815.81 to 2199.79) | 0.32 (0.17 to 0.47) |
| Senegal | 14381 (8687 to 21831) | 891.09 (542.03 to 1342.26) | 39298 (22660 to 61613) | 1048.46 (607.3 to 1634.75) | 0.18 (0.01 to 0.35) |
| Serbia | 18827 (11603 to 27879) | 796.24 (490.11 to 1180.62) | 18163 (10697 to 28398) | 865.36 (505.35 to 1361.97) | -0.41 (-0.63 to -0.19) |
| Seychelles | 96 (58 to 147) | 552.99 (337.79 to 837.63) | 167 (97 to 266) | 682.31 (394.42 to 1089.03) | 0.08 (-0.12 to 0.28) |
| Sierra Leone | 9913 (5970 to 15121) | 1037.48 (627.24 to 1568.41) | 24353 (14025 to 39314) | 1136 (657.17 to 1822.36) | 0.35 (0.28 to 0.41) |
| Singapore | 9424 (6100 to 13531) | 990.39 (641.14 to 1422.4) | 9038 (5371 to 13866) | 660.4 (391.27 to 1022.37) | -2.05 (-2.4 to -1.71) |
| Slovakia | 9893 (6112 to 14911) | 739.37 (455.71 to 1115.25) | 12097 (6989 to 19272) | 920.91 (529.73 to 1478.06) | -0.13 (-0.44 to 0.18) |
| Slovenia | 5265 (3268 to 7913) | 1044.23 (647.99 to 1571.23) | 4370 (2636 to 6888) | 999.97 (598.32 to 1596.62) | -0.85 (-1.09 to -0.61) |
| Solomon Islands | 506 (306 to 778) | 702.28 (427.37 to 1070.82) | 1239 (737 to 1935) | 730.11 (435.28 to 1137.36) | -0.17 (-0.26 to -0.09) |
| Somalia | 19267 (11669 to 29173) | 1188.74 (721.15 to 1795.98) | 67030 (38341 to 106853) | 1453.96 (837.71 to 2312.22) | 0.12 (-0.02 to 0.27) |
| South Africa | 105766 (69477 to 153490) | 1151.3 (756.68 to 1661.71) | 223066 (143678 to 326685) | 1429.31 (920.11 to 2093.27) | 0.33 (0.06 to 0.6) |
| South Sudan | 13792 (8295 to 20910) | 1140.95 (690.5 to 1711.21) | 27328 (15537 to 43751) | 1231.81 (702.92 to 1969.91) | 0.09 (0 to 0.18) |
| Spain | 124075 (82680 to 174206) | 1288.11 (858.2 to 1808.1) | 187733 (113479 to 293365) | 1897.46 (1137.06 to 2989.99) | 1.34 (0.95 to 1.74) |
| Sri Lanka | 38769 (24326 to 57732) | 847.29 (531.37 to 1259.18) | 45451 (27839 to 70624) | 806.41 (493.23 to 1256.48) | -0.93 (-1.24 to -0.63) |
| Sudan | 58226 (34356 to 91354) | 1271.73 (752.63 to 1980.1) | 153710 (89992 to 246710) | 1379.35 (812.23 to 2211.9) | -0.16 (-0.32 to 0) |
| Suriname | 1226 (757 to 1867) | 1275.25 (789.56 to 1936.93) | 2674 (1550 to 4295) | 1839.78 (1065.99 to 2958.26) | 0.38 (0.16 to 0.6) |
| Sweden | 31850 (20563 to 46214) | 1534.52 (987.34 to 2233.39) | 40200 (25202 to 60159) | 1833.59 (1145.55 to 2752.3) | 0.1 (-0.07 to 0.27) |
| Switzerland | 25481 (16218 to 37940) | 1433.97 (908.95 to 2139.17) | 28233 (16703 to 43998) | 1416.07 (834.2 to 2208.67) | -0.79 (-1.23 to -0.35) |
| Syrian Arab Republic | 31304 (18089 to 49282) | 1185.23 (690.53 to 1857.45) | 53507 (30205 to 87385) | 1385.84 (785.25 to 2261.57) | 0.13 (-0.05 to 0.31) |
| Taiwan (Province of China) | 26616 (16414 to 39689) | 494.61 (305.73 to 736.8) | 34688 (20578 to 53035) | 573.25 (337.38 to 883.74) | 0.4 (0.36 to 0.45) |
| Tajikistan | 8341 (5027 to 12644) | 728.32 (441.75 to 1097.48) | 21380 (12645 to 34231) | 847.82 (501.44 to 1352.89) | -0.09 (-0.3 to 0.12) |
| Thailand | 102403 (63455 to 154129) | 648.6 (402.77 to 973.18) | 117595 (70385 to 182765) | 692.43 (410.25 to 1082.52) | -0.22 (-0.32 to -0.11) |
| Timor-Leste | 1293 (789 to 1964) | 703.42 (430.33 to 1064.68) | 2384 (1375 to 3783) | 701.19 (408.02 to 1103.33) | -0.54 (-0.72 to -0.35) |
| Togo | 8557 (5147 to 12953) | 1067.22 (648.25 to 1602.87) | 23621 (13740 to 36931) | 1123.64 (656.82 to 1751.85) | 0.03 (-0.04 to 0.1) |
| Tokelau | 2 (1 to 4) | 704.26 (422.09 to 1110.22) | 3 (1 to 4) | 789.77 (453.17 to 1281.81) | 0.1 (0.03 to 0.17) |
| Tonga | 132 (81 to 201) | 605.09 (374.02 to 914.25) | 161 (98 to 251) | 641.23 (391.8 to 995.49) | -0.09 (-0.18 to 0) |
| Trinidad and Tobago | 3450 (2100 to 5228) | 1128.15 (686.54 to 1706.63) | 4849 (2788 to 7946) | 1422.79 (819.24 to 2336.64) | -0.13 (-0.4 to 0.14) |
| Tunisia | 28844 (17008 to 45123) | 1452.19 (859.81 to 2261.87) | 58530 (33409 to 95286) | 1891.13 (1076.62 to 3086.41) | 0.14 (-0.12 to 0.39) |
| Turkey | 175649 (114729 to 250495) | 1256.23 (820.5 to 1786.11) | 324715 (180029 to 530476) | 1495.08 (826.51 to 2448.84) | 0.19 (0.03 to 0.36) |
| Turkmenistan | 6546 (3995 to 9900) | 768.44 (468.29 to 1159.77) | 10898 (6324 to 17340) | 867.85 (503.37 to 1379.95) | -0.08 (-0.25 to 0.09) |
| Tuvalu | 17 (10 to 27) | 701.36 (414.24 to 1103.03) | 23 (13 to 37) | 784.16 (455.18 to 1274.9) | 0.14 (0.06 to 0.21) |
| Uganda | 58827 (35493 to 90857) | 1649.28 (1002.22 to 2532.89) | 178092 (102182 to 285142) | 1803.69 (1039.96 to 2881.94) | -0.45 (-0.74 to -0.16) |
| Ukraine | 137384 (85892 to 204350) | 1060.67 (662.7 to 1580.92) | 137273 (82873 to 210866) | 1273.35 (768.55 to 1971.37) | -0.42 (-0.66 to -0.18) |
| United Arab Emirates | 4060 (2426 to 6324) | 1211.94 (727.69 to 1879.79) | 23848 (13668 to 38126) | 1303.06 (747.1 to 2114.92) | -0.29 (-0.49 to -0.09) |
| United Kingdom | 203071 (128927 to 299331) | 1421.17 (902.34 to 2095.25) | 240555 (151621 to 359483) | 1556.88 (980.24 to 2332.86) | -0.13 (-0.45 to 0.2) |
| United Republic of Tanzania | 62976 (38387 to 95387) | 1121.29 (687.62 to 1685.16) | 174814 (101458 to 273557) | 1224.06 (713.36 to 1901.39) | -0.04 (-0.14 to 0.06) |
| United States of America | 807796 (535196 to 1158571) | 1206.85 (798.34 to 1735.92) | 1480345 (990610 to 2119857) | 1982.29 (1326.01 to 2844.07) | 0.69 (0.4 to 0.98) |
| United States Virgin Islands | 244 (146 to 380) | 843.28 (504.73 to 1313.81) | 183 (102 to 289) | 1050.48 (587.73 to 1656.63) | 0.28 (0.1 to 0.47) |
| Uruguay | 6840 (4131 to 10492) | 911.39 (550.34 to 1398.16) | 11067 (6381 to 17861) | 1335.28 (769.95 to 2160.47) | 0.87 (0.66 to 1.09) |
| Uzbekistan | 37146 (23094 to 55738) | 790.4 (492.23 to 1178.43) | 77480 (46041 to 121500) | 864.57 (512.96 to 1357.1) | -0.11 (-0.25 to 0.03) |
| Vanuatu | 239 (146 to 365) | 694.02 (425.95 to 1055.65) | 559 (328 to 872) | 717.93 (422.89 to 1114.82) | -0.19 (-0.28 to -0.1) |
| Venezuela (Bolivarian Republic of) | 38563 (22686 to 59967) | 828.65 (486.8 to 1284.14) | 64542 (36354 to 103397) | 923.24 (519.4 to 1480.02) | 0.06 (-0.05 to 0.17) |
| Viet Nam | 101445 (62236 to 153175) | 615.34 (378.49 to 926.16) | 169475 (101028 to 261999) | 657.25 (388.72 to 1021.77) | -0.3 (-0.44 to -0.17) |
| Yemen | 40636 (23972 to 63904) | 1523.46 (901.25 to 2386.08) | 123888 (69060 to 199980) | 1517.75 (848.41 to 2442.57) | -0.12 (-0.17 to -0.06) |
| Zambia | 16273 (10031 to 24578) | 956.99 (592.65 to 1434.37) | 51080 (29812 to 80073) | 1082.81 (634.87 to 1684.12) | -0.07 (-0.21 to 0.06) |
| Zimbabwe | 16118 (9799 to 24588) | 725.87 (443.41 to 1095.82) | 33631 (20126 to 53160) | 856.27 (513.93 to 1349.79) | 0.16 (0 to 0.31) |

**Supplementary Figure S1**

The numbers and rates of incidence (A), prevalence (B), and DALYs (C) for depressive disorders among WCBA by SDI regions in 1990 and 2021. DALYs, disability-adjusted life years, WCBA, women of childbearing age, SDI, Socio-Demographic Index.


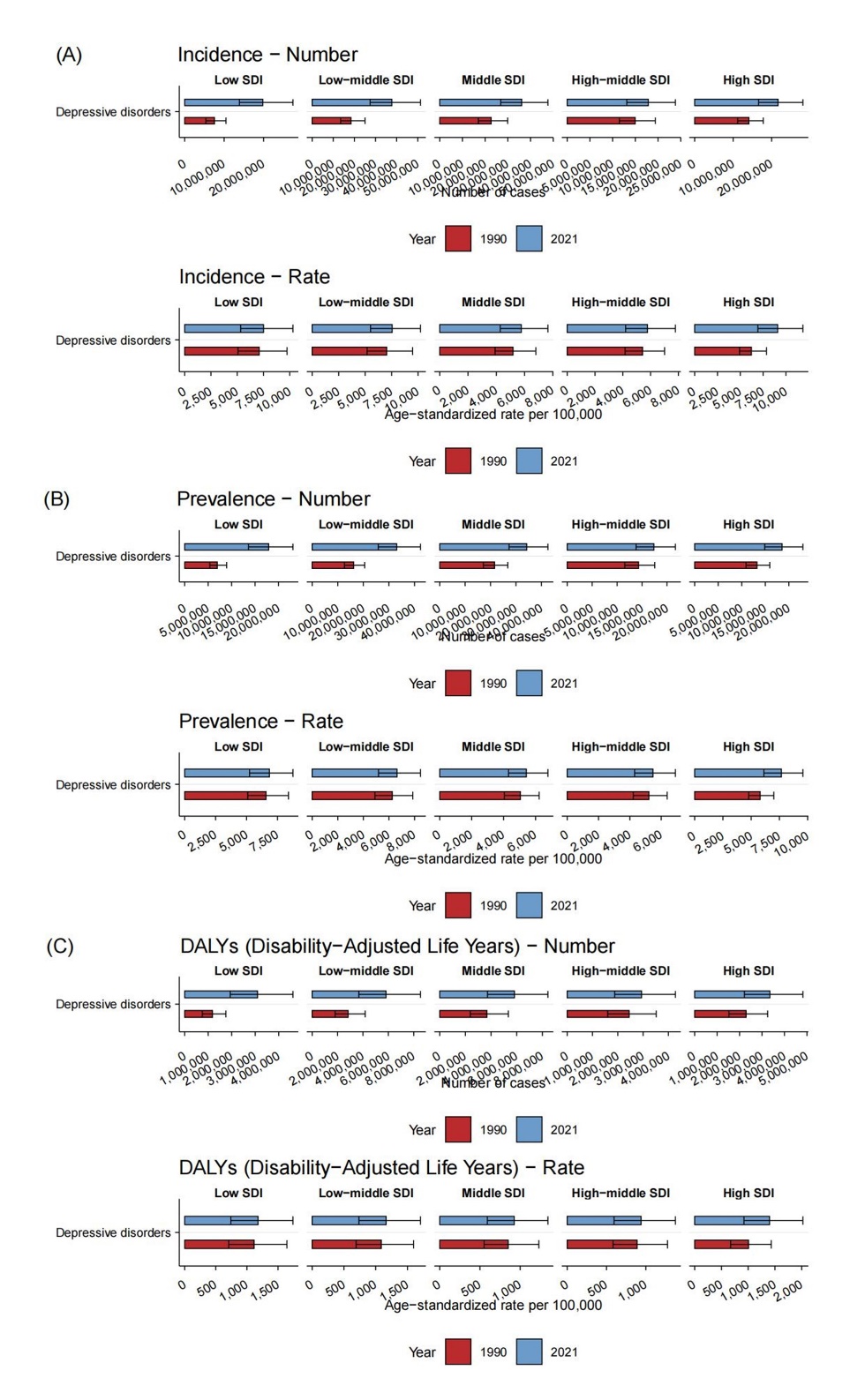


**Supplementary Figure S2**

The numbers and rates of incidence, prevalence, and DALYs for depressive disorders among WCBA by 21 regions in 1990 and 2021. DALYs, disability-adjusted life years, WCBA, women of childbearing age.


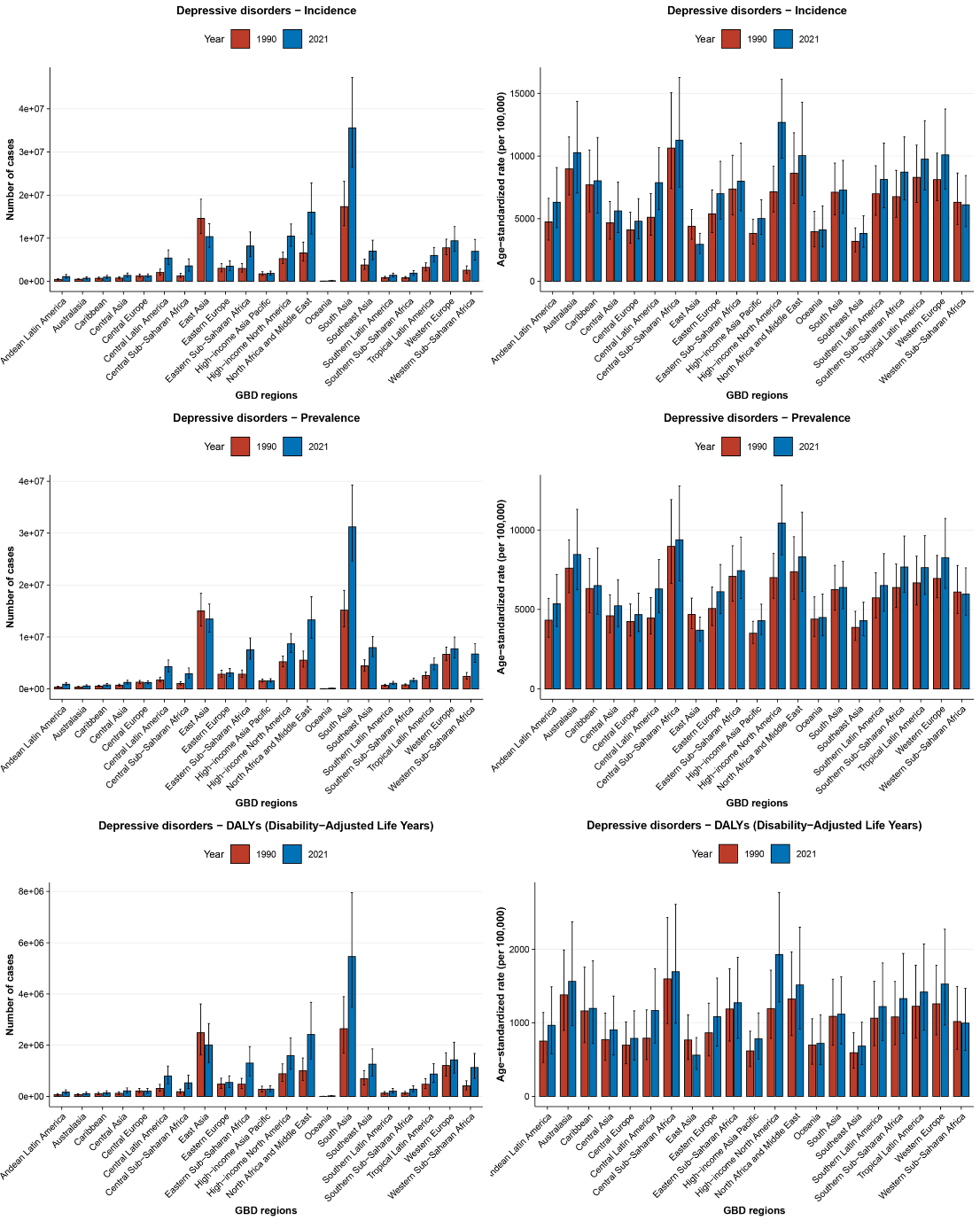


**Supplementary Figure S3**

The numbers and rates of incidence, prevalence, and DALYs for depressive disorders among WCBA in different age-groups; DALYs, disability-adjusted life years; WCBA, women of childbearing age.


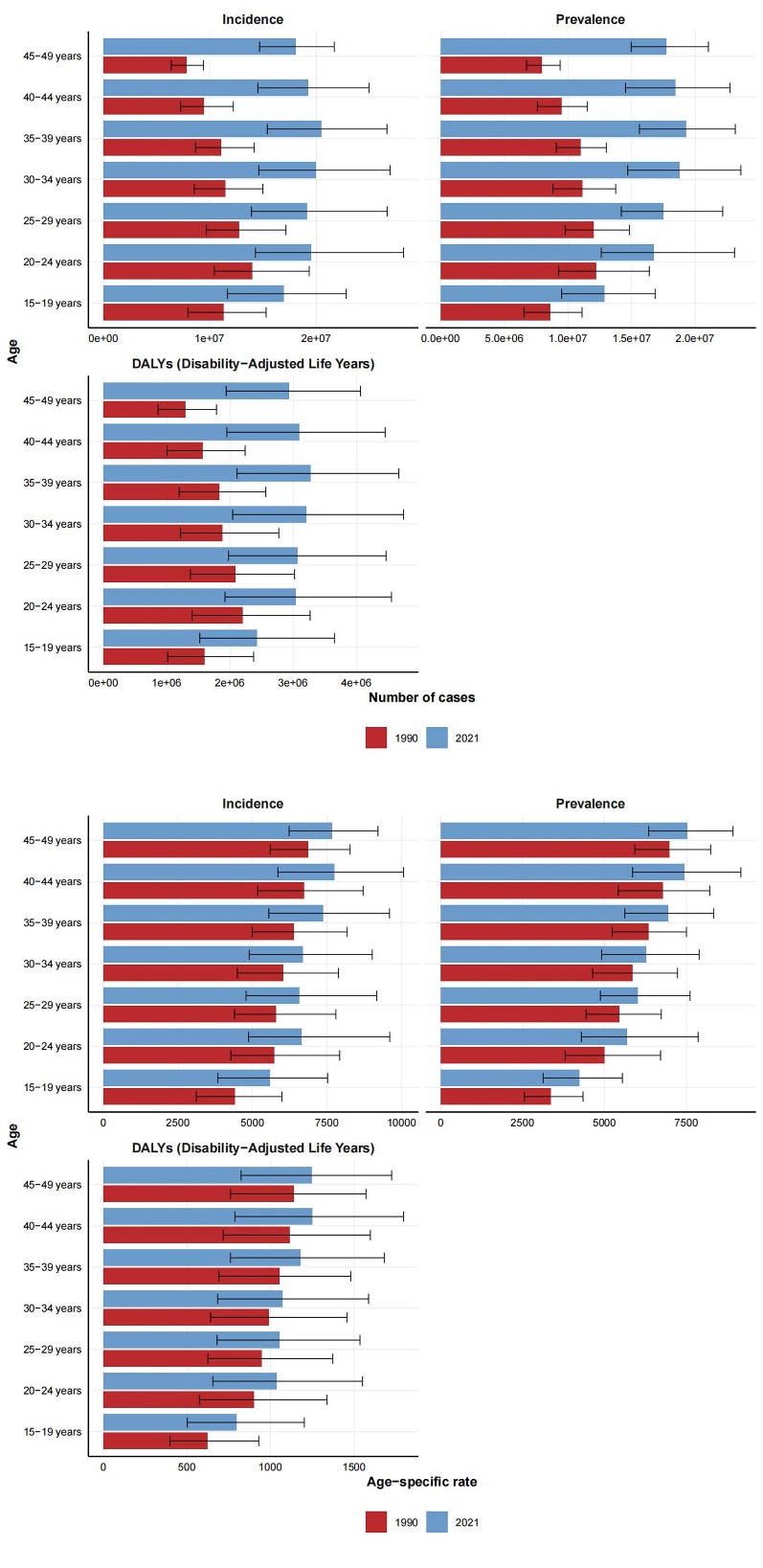


**Supplementary Figure S4**

The trends of incidence, prevalence, and DALYs for depressive disorders among WCBA in different age-groups from 1990 to 2021. DALYs, disability-adjusted life years; WCBA, women of childbearing age.


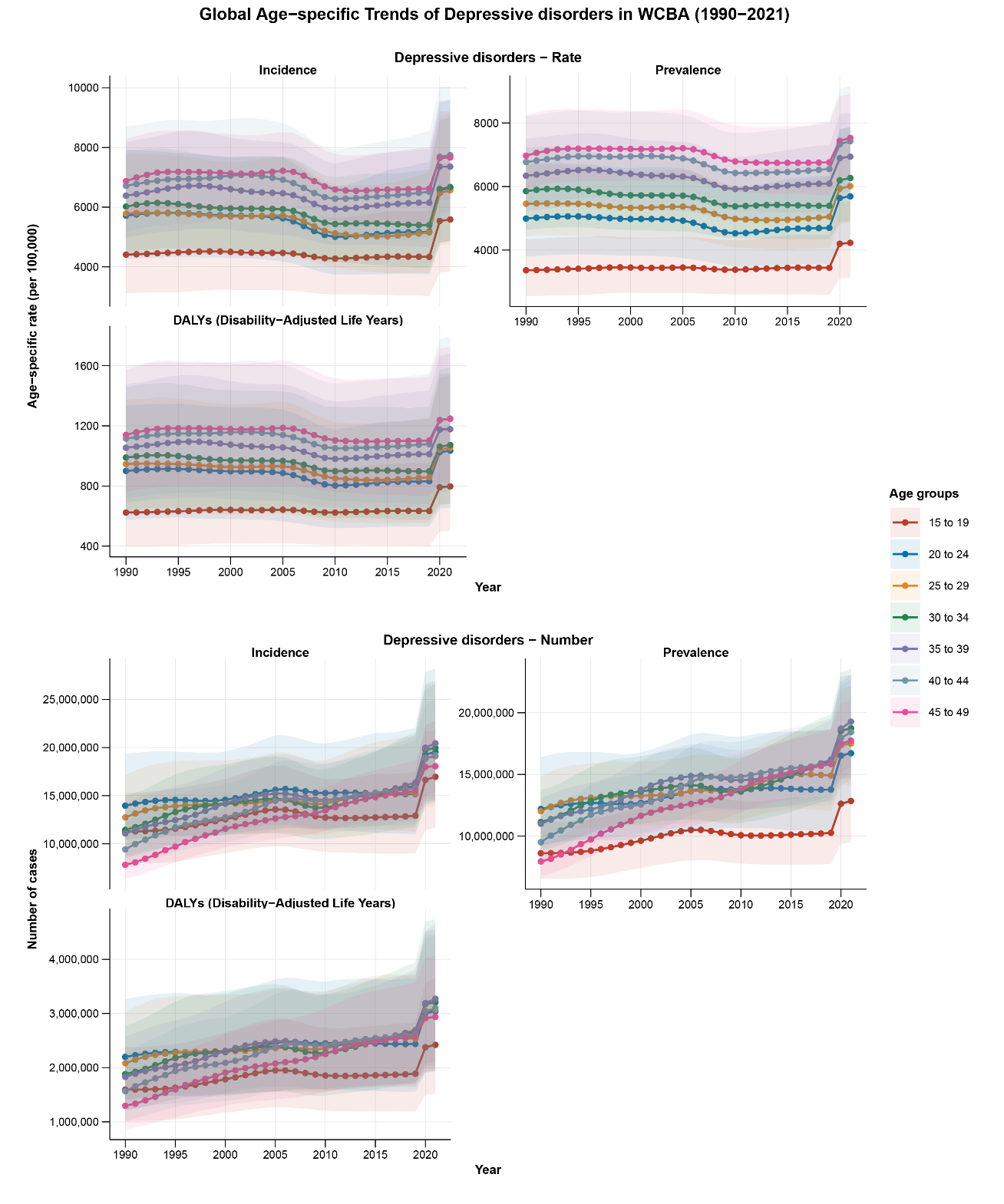

Supplement: Supplementary file 1 [file Supplementary_file_1.docx]
